# Iron-catalyzed tandem reaction of C–Se bond coupling/selenosulfonation of indols with benzeneselenols†

**DOI:** 10.1039/d0ra05922f

**Published:** 2020-07-20

**Authors:** Senling Guan, Yue Chen, Hongjie Wu, Runsheng Xu

**Affiliations:** Department of Biology and Environment, Jiyang College of Zhejiang A&F University Shaoxing 311800 Zhejiang China 20140041@zafu.edu.cn

## Abstract

An iron-catalyzed tandem reaction of C–Se bond coupling/selenosulfonation was developed. Starting from sample indols and benzeneselenols versatile biologically active 2-benzeneselenonyl-1*H*-indoles derivatives were efficiently synthesized. The reaction mechanism was studied by the deuterium isotope study and *in situ* ESI-MS experiments. This protocol features mild reaction conditions, wider substrate scope and provides an economical approach toward C(sp^2^)–Se bond formation.

## Abstract 2

Iron-catalyzed tandem reaction of C–Se bond coupling/selenosulfonation.

## Graphical abstract

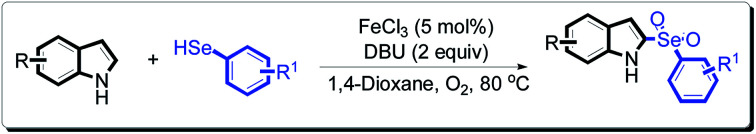

Due to the important applications in the preparation of synthetic materials,^1^ pharmaceutical agents,^2^ fluorescent probes,^3^ and functional organic materials,^4^ organoselenium compounds synthesis has attracted extensive attention from synthetic chemists. It is known that transition-metal catalyzed cross coupling reaction is the mostly used methodology for the incorporation of a Se atom into aromatic frameworks.^5^ However, prefunctionalization of the substrate is generally requested. Similar methods of C(sp^2^)–Se bonds formation have been scarcely described.^6–8^

Comparative to the C(sp)–H, the C(sp^2^)–H bond activation need more harsh conditions and activated reaction systems.^9^ Considering the significance of diversifying synthetic strategies, our group focuses on tradition-metal catalyzed C–H bond functionalizations.^10^ Herein, we report a novel iron-catalyzed direct C(sp^2^)–H bond activation/C–Se cross coupling reaction of indols with benzeneselenols. Versatile biologically active compounds 2-benzeneselenonyl-1*H*-indoles were efficiently synthesized in good to high yields. In this reaction, the inactive C(sp^2^)–H bonds were smoothly direct selenosulfonation under a moderate condition. At last, the reaction mechanism was studied by the deuterium isotope study and the *in situ* ESI-MS experiments.

At first, as shown in Table 1, the reaction conditions were screened based on the model reaction of indol 1a with benzeneselenol 2a (Table 1). The corresponding product structure of 3a was confirmed by NMR spectrums. The iron catalysts displayed a good catalytic activity (entries 1–5). In addition, FeCl_3_ exhibited superior catalytic efficiency over all of the examined iron catalysts (entry 5). These results indicated that DBU (1,8-diazabicyclo[5.4.0]undec-7-ene) and O_2_ were the optimal base and additive, which produced the product 3a with an 83% yield (entry 14). It was also noted that the product yield was decreased when the reaction temperature was less or greater than 80 °C (entries 15 and 16). Furthermore, the results also show that the reaction yield of 1,4-dioxane as a solvent is higher than that of other solvents (entries 17 and 18). In particular, those reactions had to be carried out under a strict anhydrous condition. The presence of water would reduce the Fe^3+^ concentration, and reduced the catalytic activity (entry 19). Thus, the optimum reaction condition was determined as the 1a and 2a ratio of 1 : 1.5 in the presence of FeCl_3_ (5 mol%), DBU (2 equiv.), at 80 °C for 10 hours (Table 1, entry 14).

**Table 1:** Optimization of the reaction conditions^a^

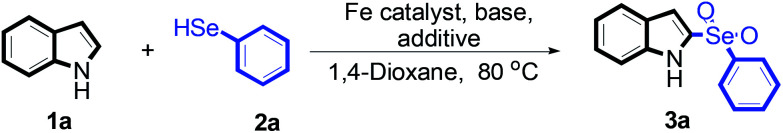					
Entry	Fe catalyst	Base	Additive	1a : 2a	Yield^b^ (%)
1	FeCl_2_	DBU	O_2_	1 : 1	0
2	FeBr_2_	DBU	O_2_	1 : 1	0
3	Fe(OAc)_2_	DBU	O_2_	1 : 1	19
4	Fe_2_(SO_4_)_3_	DBU	O_2_	1 : 1	23
5	FeCl_3_	DBU	O_2_	1 : 1	67
6	FeCl_3_	Imidazole	O_2_	1 : 1	36
7	FeCl_3_	Piperidine	O_2_	1 : 1	49
8	FeCl_3_	*N*, *N*-Dimethylaniline	O_2_	1 : 1	46
9	FeCl_3_	Tri-*n*-propylamine	O_2_	1 : 1	38
10	FeCl3	DABCO	O_2_	1 : 1	57
11	FeCl_3_	DBU	AgO	1 : 1	0
12	FeCl_3_	DBU	H_2_O_2_	1 : 1	38
13	FeCl_3_	DBU	CH_3_COOOH	1 : 1	42
14	FeCl_3_	DBU	O_2_	1 : 1.5	83
15	FeCl_3_	DBU	O_2_	1 : 1.5	65^c^
16	FeCl_3_	DBU	O_2_	1 : 1.5	82^d^
17	FeCl_3_	DBU	O_2_	1 : 1.5	64^e^
18	FeCl_3_	DBU	O_2_	1 : 1.5	77^f^
19	FeCl_3_	DBU	O_2_	1 : 1.5	23^g^

a Unless otherwise noted, reactions conditions were 1a (0.5 mmol), 2a (0.5 mmol), Fe catalyst (5 mol%), base (2 equiv.), additive (2 equiv or under atmosphere), 1,4-dioxane (4 mL), 80 °C for 10 h.

b Isolated yield.

c 70 °C.

d 90 °C.

e In CHCl_3_.

f In DMF.

g Solvents not been dried.

Next, the reaction scope was been screened, a wide array of indols 1 with benzeneselenols 2 were subjected to this reaction and given the products 3 in good to excellent yields (Table 2, 65–92% yield). It was found that both the electron-donating and electron-withdrawing indols derivatives 1 reacted smoothly with benzeneselenols 2. Furthermore, indols 1 bearing electron-withdrawing groups showed better activity than bearing electron-donating groups. Benzeneselenols 2 bearing electron-donating groups showed better activity than bearing electron-withdrawing groups. To our delight, despite the electron-withdrawing effect of –NO_2_ and –CF_3_ group is so strong, the corresponding products 3h and 3r were still obtained in 75% and 69% yield (entries 8 and 9).

**Table 2:** Iron-catalyzed tandem reaction of C–Se bond coupling/selenosulfonation of indols with benzeneselenols^a^

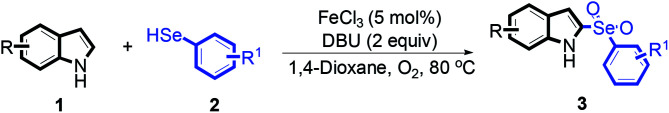				
Entry	R	R^1^	3	Yield^b^
1	H	H	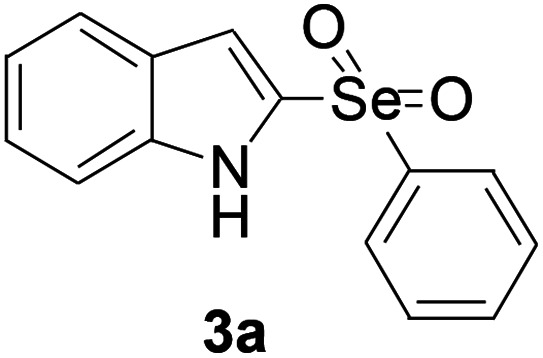	83
2	H	4-Me	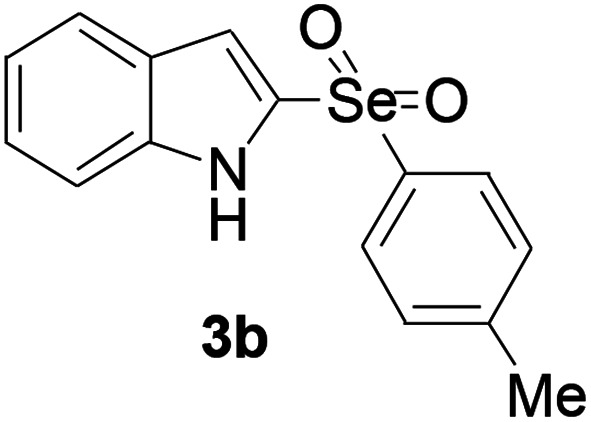	84
3	H	4-*t*Bu	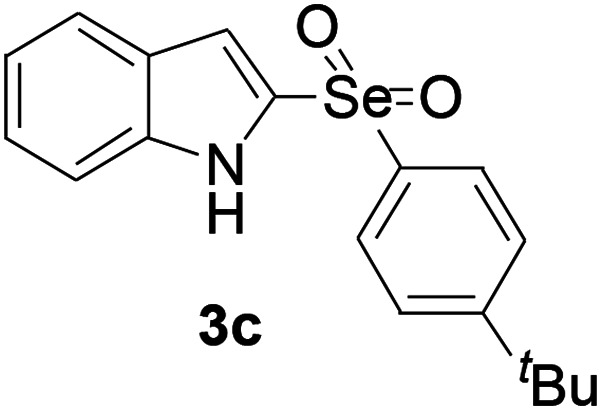	87
4	H	4-OMe	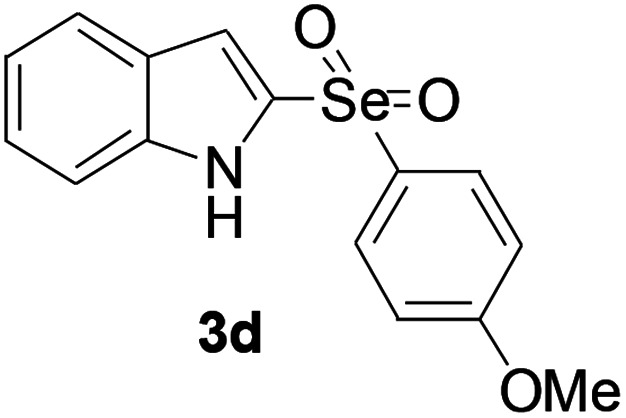	92
5	H	4-F	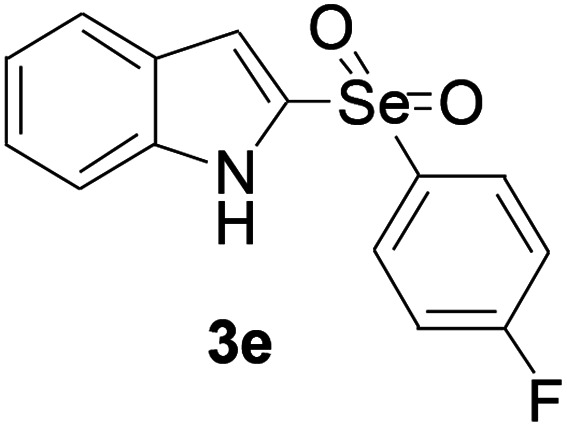	78
6	H	4-Cl	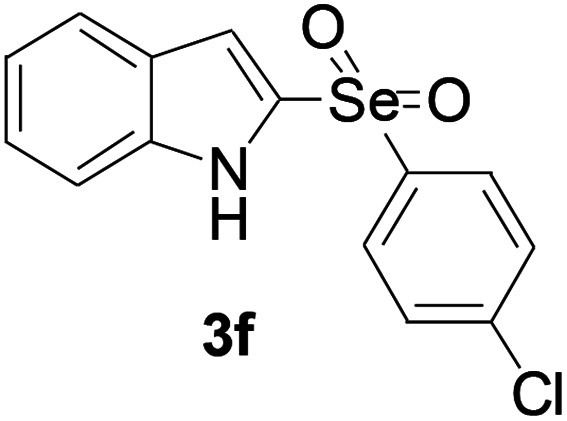	81
7	H	4-Br	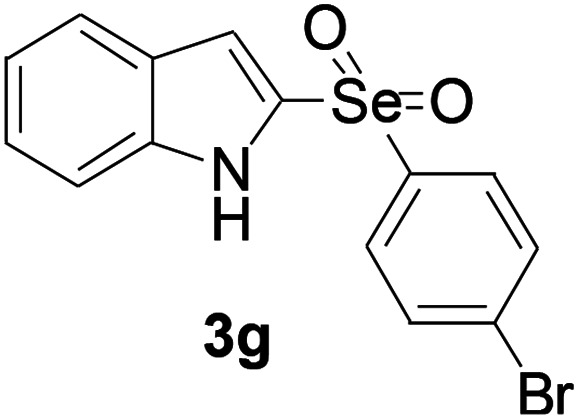	83
8	H	4-CF_3_	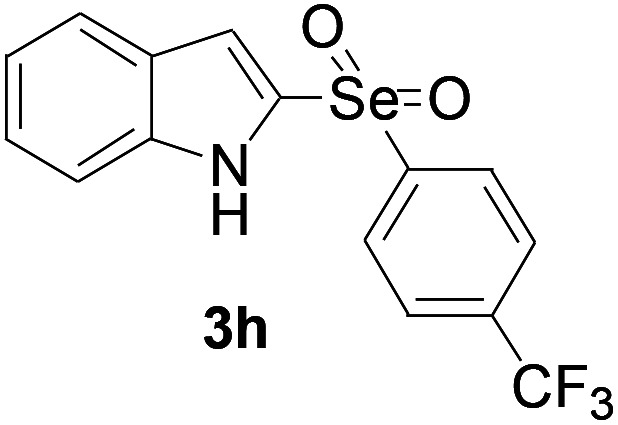	75
9	H	4-NO_2_	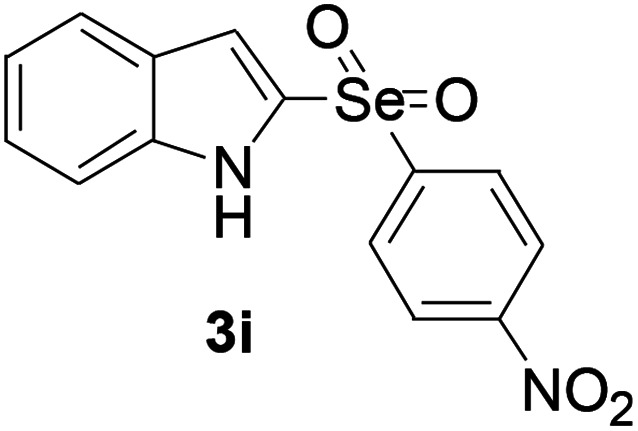	69
10	H	Naphthyl	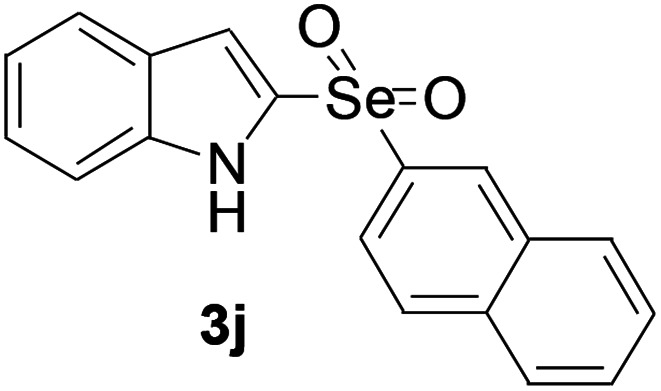	79
11	5-Me	4-Me	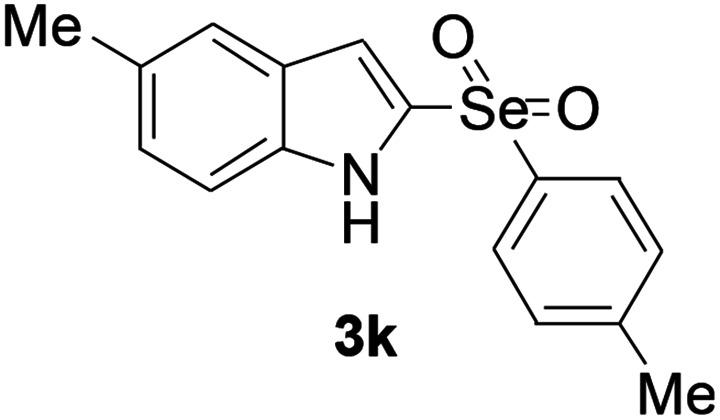	75
12	7-Me	4-Me	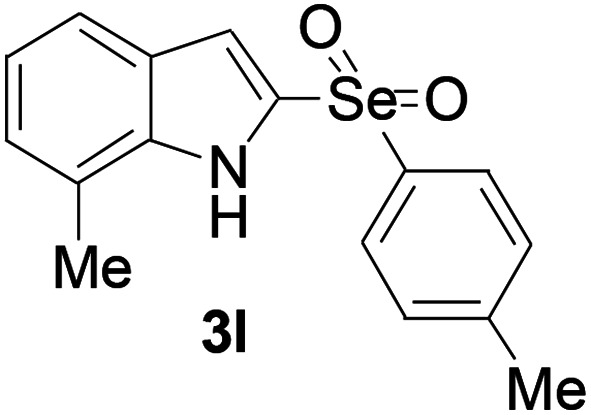	76
13	4-OMe	4-Me	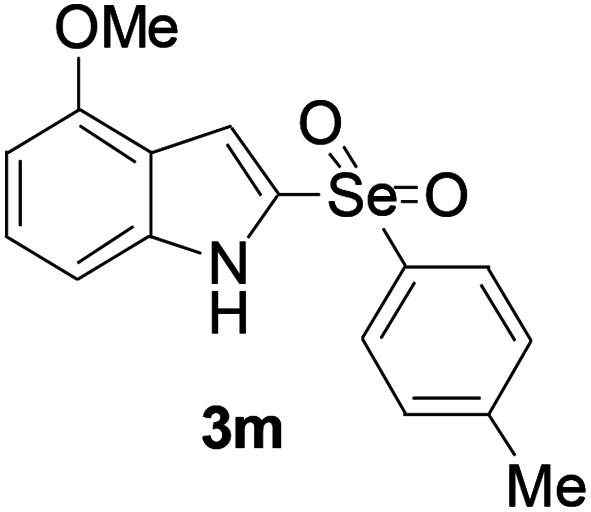	74
14	5-OMe	4-Me	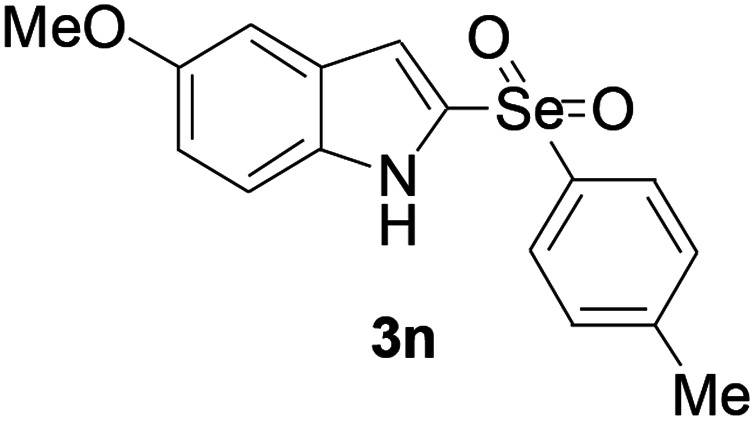	72
15	7-OMe	4-Me	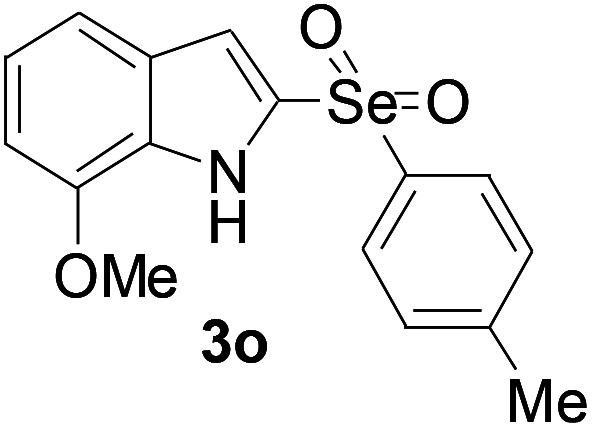	67
16	4-OCH_2_Ph	4-Me	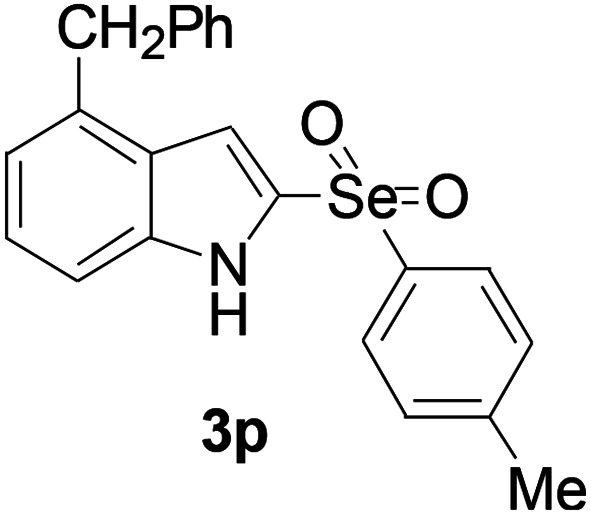	66
17	6-Cl	4-Me	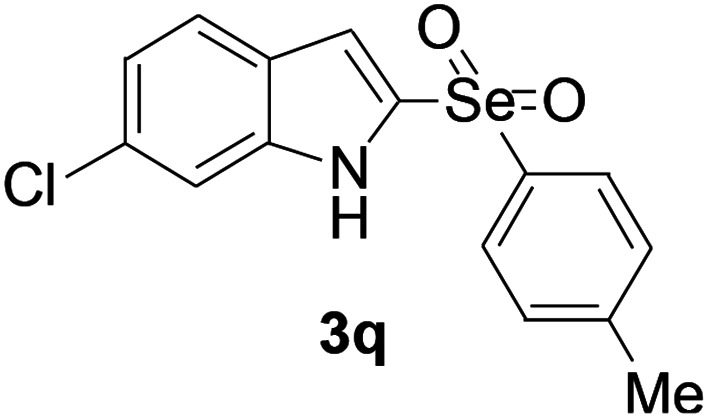	90
18	7-Cl	4-Me	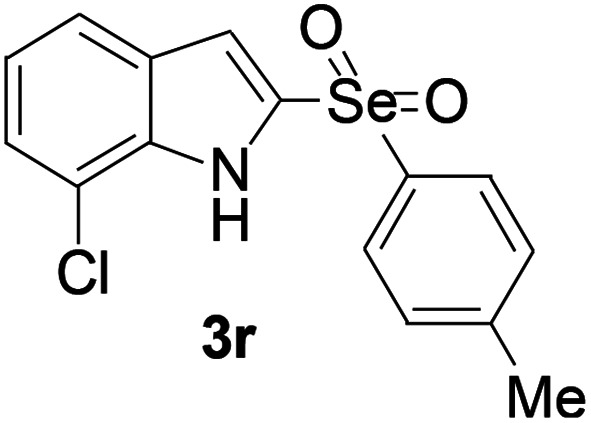	91
19	3-Me	4-Me	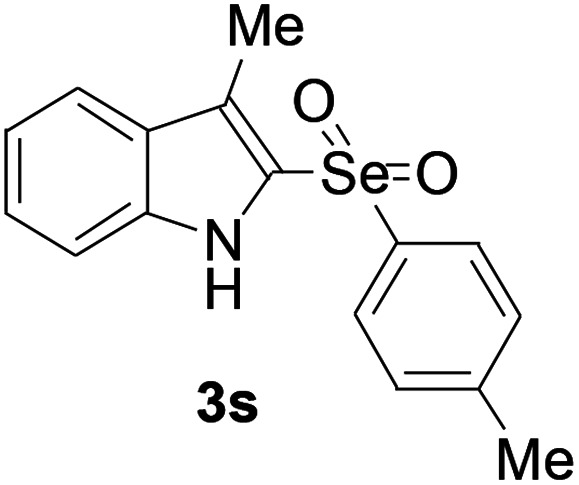	65

a Unless otherwise noted, reaction conditions were 1 (0.5 mmol), 2 (0.75 mmol), FeCl_3_ (5 mol%), DBU (2 equiv.), under a O_2_ atmosphere, 1,4-dioxane (5 mL), 80 °C for 10 h.

b Isolated yield.

Furthermore, we next focused on evaluating the generality of tandem reaction of C–Se bond coupling/selenosulfonation by using a series of pyrroles. To our delight, *N*-methylpyrrole 4 with benzeneselenols 2 successfully provided the corresponding products 5 (Table 3, 59–79% yield). For both substrates, this reaction was amenable when electroneutral group (entry 1), electron donating group (entries 2 and 3), electron-withdrawing group (entry 4–8), Moreover, the trifluoromethyl substituted delivered the product 5h exclusively in 59% yield which bearing of strong electron-withdrawing group. Furthermore, reactants with more complex substituents also perform smoothly (entry 9). Both the results demonstrated the good generality and high functional group tolerance of this method.

**Table 3:** Iron-catalyzed tandem reaction of C–Se bond coupling/selenosulfonation of *N*-methylpyrrole with benzeneselenols^a^

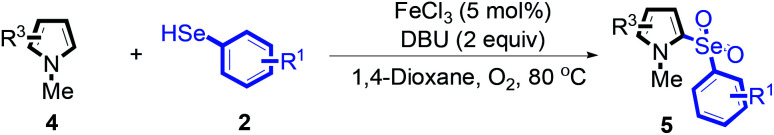				
Entry	R^3^	R^1^	5	Yield^b^
1	H	H	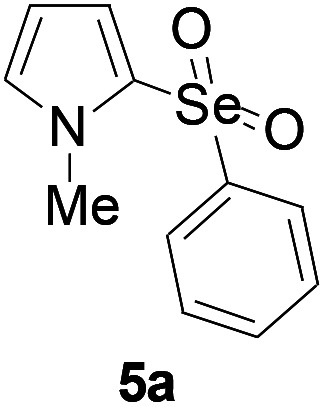	76
2	H	4-Me	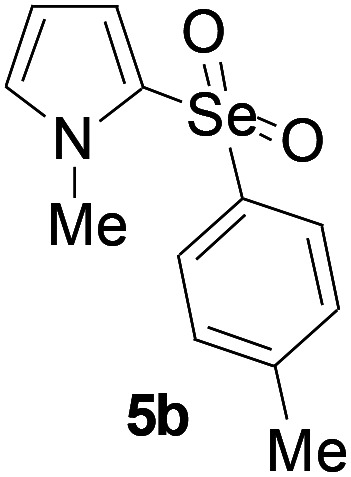	79
3	H	4-*t*Bu	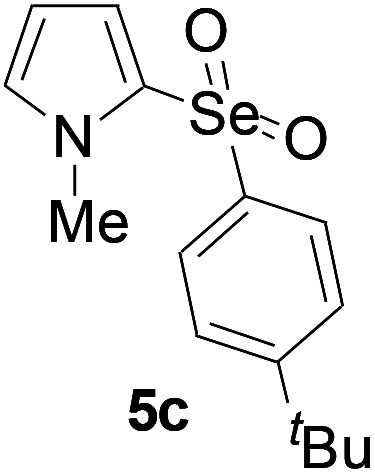	78
4	H	4-F	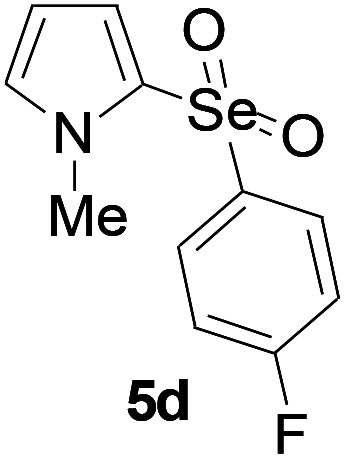	69
5	H	4-Cl	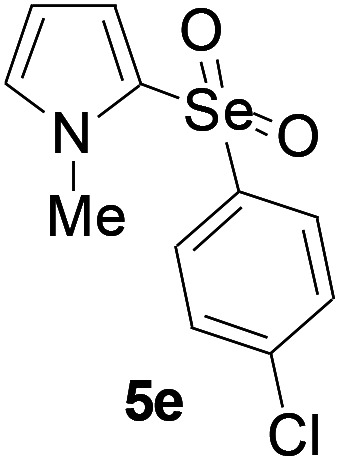	65
6	H	4-Br	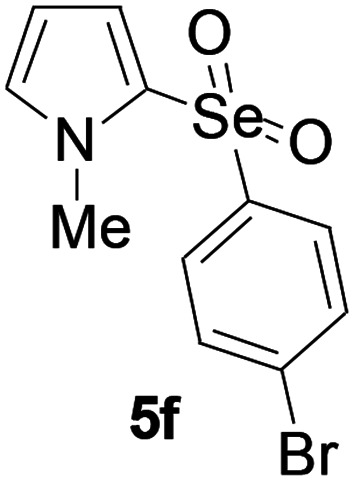	66
7	H	3-Br	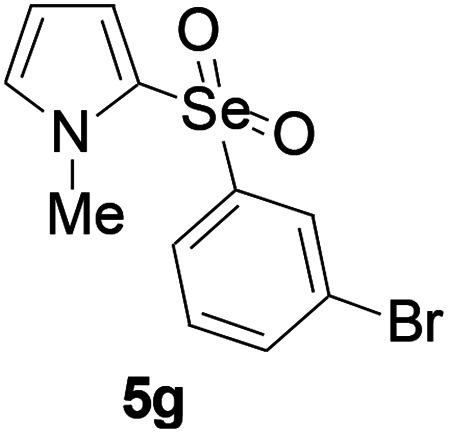	68
8	H	4-CF_3_	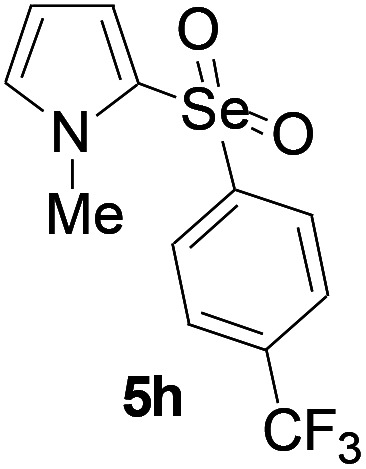	59
9	H	Naphthyl	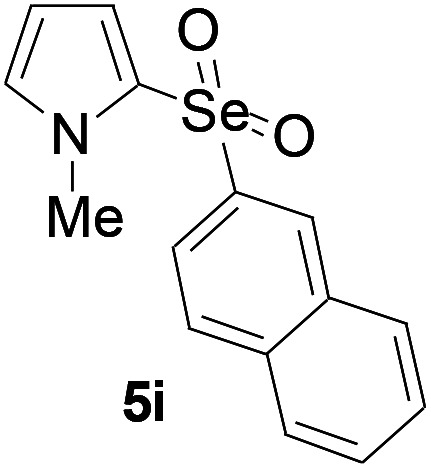	70

a Unless otherwise noted, reaction conditions were 4 (0.5 mmol), 2 (0.75 mmol), FeCl_3_ (5 mol%), DBU (2 equiv.), under a O_2_ atmosphere, 1,4-dioxane (5 mL), 80 °C for 10 h.

b Isolated yield.

To obtain the preliminary data of the mechanism, some addition reactions were been done (Scheme 1). At first, the model reaction (Scheme 1I) was conducted in two separate steps: the C–Se cross coupling reaction of 6 with 2a given a product 7 (Scheme 1II, 85% yield).^11^ Next, 7 was reacted under our standard conditions, the reaction successfully obtained the target product 3a (Scheme 1III 79% yield), indicating that the intermediate 7 was involved in the reaction mechanism.

**Scheme 1:**
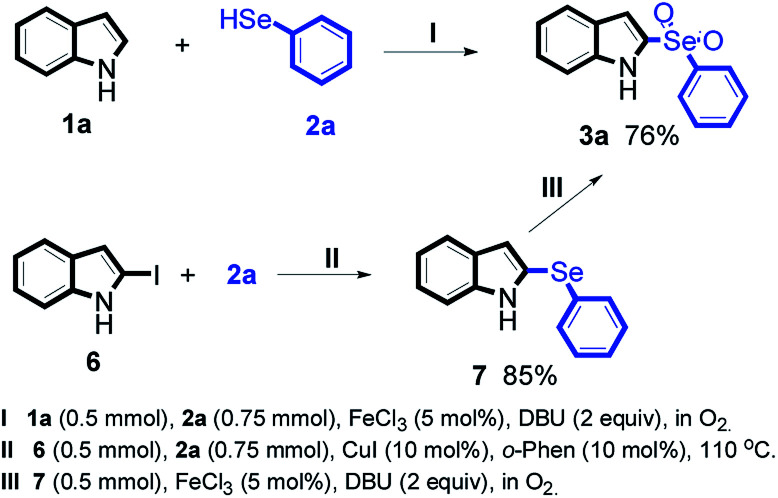
Preliminary data of the reaction mechanism.

Next, we used isotope experiments to further study the reaction mechanism, as shown in Scheme 2. The kinetic deuterium isotope effects^12^ observed in the control experiments were indicated that the C(sp^2^)–H cleavage being the rate-limiting step (*k*_H_/*k*_D_ = 1.3, for detail information please see ESI†).

**Scheme 2:**
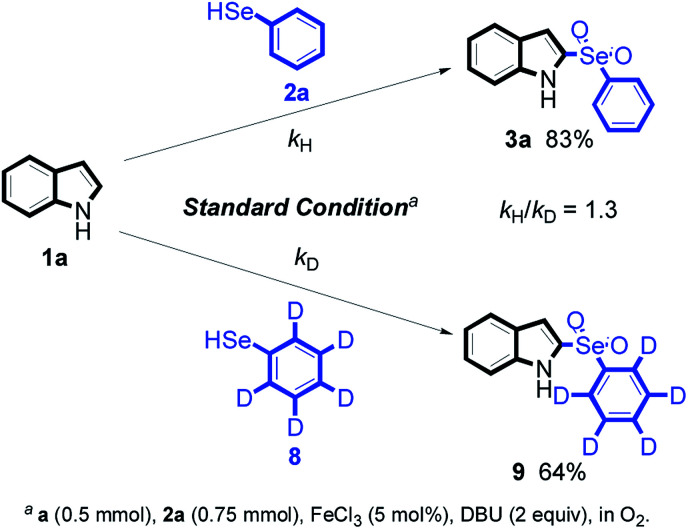
The kinetic deuterium isotope effects.

Additionally, the model reaction mixture^13^ was subjected to the *in situ* ESI-MS analysis which the detection temperature was enacted at 120 °C (Scheme 3). The positive-ion mode ESI-MS showed a peak at 296.0 (*m*/*z*) which corresponding to [C_14_H_11_NNaSe]^+^. The peak at 328.0 was assigned to [C_14_H_11_NNaO_2_Se]^+^ (Scheme 3a). Meanwhile, using the ^18^O_2_ deuterium labeling study gave a peak at 331.9 was assigned to [C_14_H_11_NNa^18^O_2_Se]^+^ (Scheme 3b), also further validated the intermediate components hypothesis (For ESI HR-MS, please see ESI†).^14^

**Scheme 3:**
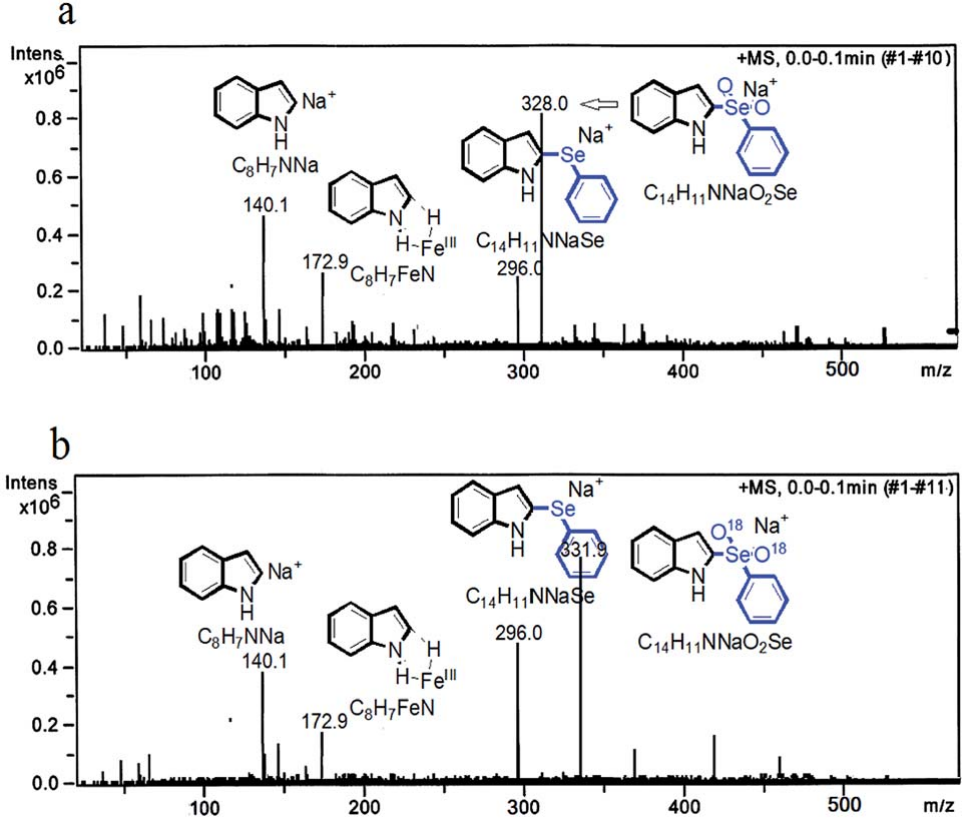
The *in situ* ESI-MS spectras of iron-catalyzed direct C(sp^2^)–H bond activation/C–Se cross coupling ((a) for the mode reaction, (b) for the ^18^O_2_ deuterium labeling reaction).

Based on these results, we proposed a possible reaction mechanism (Scheme 4). At the beginning of the reaction, the coordination process of Fe^III^ and reactant 2 generated a intermediate 10. Then, reactant 1 was converted to intermediate 11 by reacted with DBU. Next, intermediate 12 was provided from intermediate 10 with 11*via* C–Se bond cross coupling. At last, through the oxidation reaction by O_2_, intermediate 12 generated the desired products 3 and concomitantly formed a Fe^III^ intermediate, which re-entered the catalytic cycle.

**Scheme 4:**
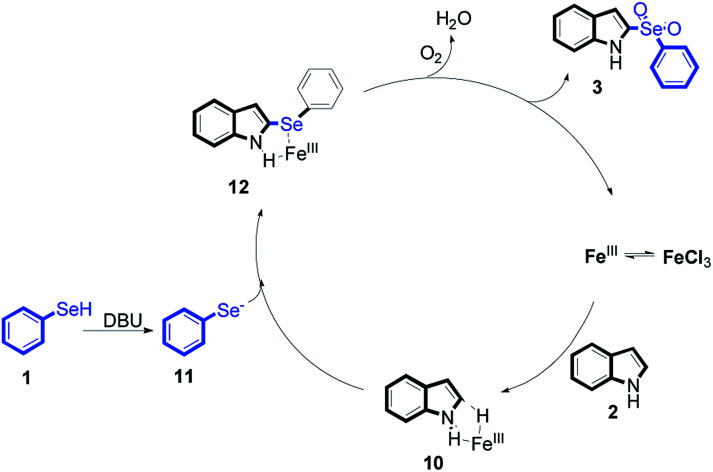
Proposed mechanism.

## Conclusions

In summary, we have reported an iron-catalyzed tandem reaction of C–Se bond coupling/selenosulfonation. Starting from sample indols and benzeneselenols versatile biologically active 2-benzeneselenonyl-1*H*-indoles derivatives were efficiently synthesized. The reaction mechanism was studied by the deuterium isotope study and *in situ* ESI-MS experiments. This protocol features mild reaction conditions, wider substrate scope and provides an economical approach toward C(sp^2^)–Se bond formation.

## Conflicts of interest

There are no conflicts to declare.

## Supplementary Material

RA-010-D0RA05922F-s001
