# Supplementary material for: Iron-catalyzed tandem reaction of C–Se bond coupling/selenosulfonation of indols with benzeneselenols
Source: RSC Adv. 2020 Jul 20;10(45):27058–63. doi: 10.1039/d0ra05922f (PMC9055526; doi:10.1039/d0ra05922f)
Supplement: RA-010-D0RA05922F-s001 [file RA-010-D0RA05922F-s001.pdf]

Electronic Supplementary Material (ESI) for RSC Advances.
This journal is © The Royal Society of Chemistry 2020

Electronic Supplementary Information

Iron-catalyzed Tandem Reaction of C-Se Bond Coupling/Selenosulfonation of Indols with Benzeneselenols

Senling Guan, Yue Chen, Hongjie Wu and Runsheng Xu*
Department of Biology and Environment, Jiyang College of Zhejiang Agriculture and Forestry University, Zhejiang 311800, China;
* Correspondence: 20140041 @zafu.edu.cn; Tel.: +86-0575-87761234

Contents

Experimental Details......2
Mechanism Study......2
Analytical Data of 3, 5......4
Spectrums of 3, 5......15
Spectrums of ESI HR-MS......44

Experimental Details

General Information

All reagents used in experiment were obtained from commercial sources and used without further purification. Solvents for chromatography were technical grade and distilled prior for using. Solvent mixtures were understood as volume/volume. Chemical yields refer to pure isolated substances. Catalysts were purchased from Alfa Aesar (Analytical reagent). Thin layer chromatography (TLC) employed glass 0.25 mm silica gel plates with F-254 indicator, visualized by irradiation with UV light.

The NMR spectra were recorded on Bruker AVANCE III-400 spectrometry at 400 MHz and 100 MHz for ^{1}H and ^{13}C NMR in $CDCl_3$, respectively. The NMR chemical shift was reported in ppm relative to 7.26 and 77 ppm of $CDCl_3$ as the standards of ^{1}H and ^{13}C NMR, respectively. The NMR spectra were reported in delta (δ) units, parts per million (ppm) downfield from the internal standard and coupling constants were reported in Hertz (Hz). Multiplicities were indicated s (singlet), d (doublet), t (triplet), q (quartet), m (multiplet). The mass spectra were performed on a Bruker Esquire 3000plus mass spectrometer equipped with ESI interface and ion trap analyzer. The ESI HR-MS were tested on Bruker 7-tesla FT-ICR MS equipped with an electrospray source.

General procedure for preparation of 3 and 5

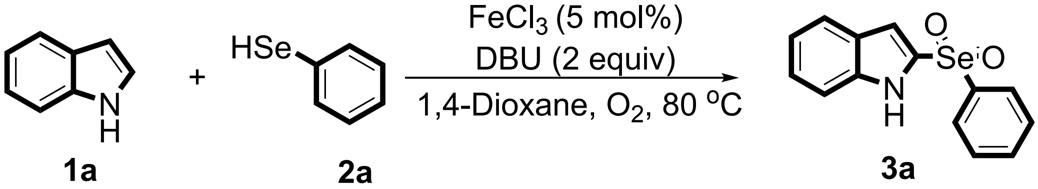

A mixture of indol **1a** (0.5 mmol, 59 mg), benzeneselenol **2a** (0.75 mmol, 118 mg), $FeCl_3$ (5 mol%, 4 mg) and DBU (2 equiv, 152 mg), in 1,4-dioxane (5 mL) was stirred under a O_2 atmosphere. After the reaction mixture was stirred at 80 °C for 10 h, it was allowed to cool to ambient temperature. Then the mixture was quenched with saturated salt water (10 mL), and the solution was extracted with ethyl acetate (3 × 10 mL). The organic layers were combined and dried by sodium sulfate and concentrated in vacuo. The pure product 2-benzeneselenonyl-1H-indole **3a** (126 mg, 83% yield) was obtained by flash column chromatography on silica gel.

Mechanism Study

Procedure for preparation of 7

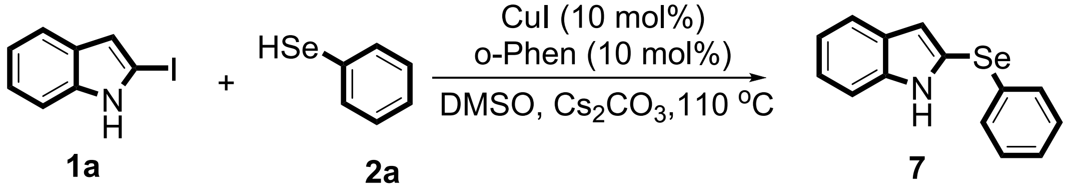

A mixture of indol **1a** (0.5 mmol, 59 mg), benzeneselenol **2a** (0.75 mmol, 118 mg), CuI (10 mol%, 19 mg) and Cs_2CO_3 (2 equiv, 326 mg), in DMSO (5 mL) was stirred under a N_2 atmosphere. After the reaction mixture was stirred at 110 °C for 12 h, it was allowed to cool to ambient temperature. Then the mixture was quenched with saturated salt water (10 mL), and the solution was extracted with ethyl acetate (3 × 10 mL). The organic layers were combined and dried by sodium sulfate and concentrated in vacuo. The pure product 2-phenylselanyl-*1H*-indole **7** (Yellow solid, m.p. 155-157 °C, 116 mg, 85%) was obtained by flash column chromatography on silica gel.

Synthesis Method of 8

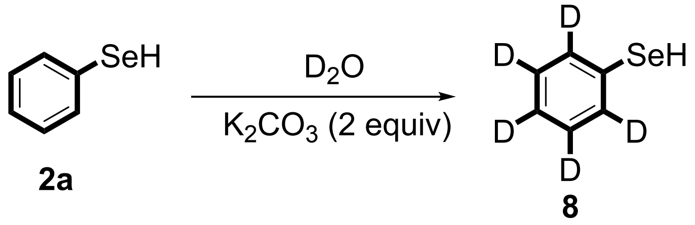

Benzeneselenol (79 mg, 0.5 mmol) was dissolved in D_2O (5 mL) in the presence of K_2CO_3(138 mg, 1.0 mmol). The reaction was stopped after 10 h, and the mixture of **8** was analyzed by ^{13}C NMR spectroscopy. ^{13}C NMR (101 MHz, $CDCl_3$): δ 130.45, 128.12, 128.13, 127.01, 127.00.

KIE Experiment

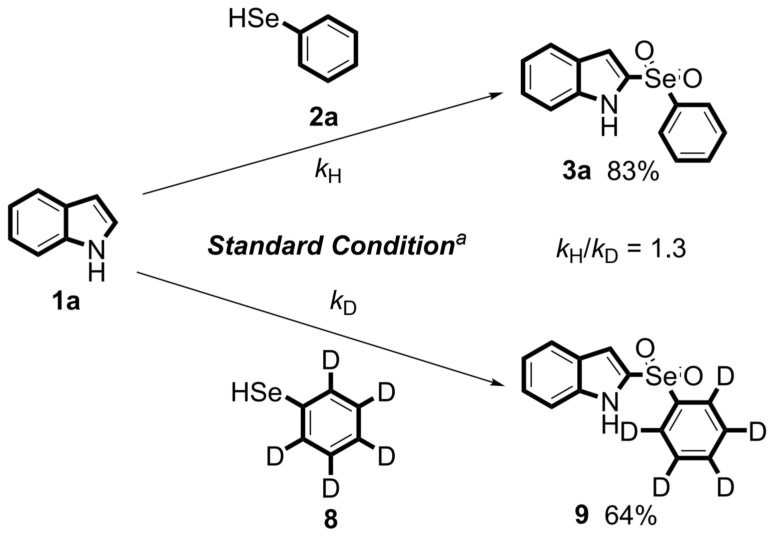

A mixture of indol **1a** (0.5 mmol, 59 mg), benzeneselenol **2a** (0.75 mmol, 118 mg) or **8** (0.75 mmol, 122 mg), $FeCl_3$ (5 mol%, 4 mg) and DBU (2 equiv, 152 mg), in 1,4-dioxane (5 mL) was stirred under a O_2 atmosphere. After the reaction mixture was stirred at 80 °C for 10 h, it was allowed to cool to ambient temperature. Then the mixture was quenched with saturated salt water (10 mL), and the solution was extracted with ethyl acetate (3 × 10 mL). The organic layers were combined and dried by sodium sulfate and concentrated in vacuo. The pure product **3a** and **9** was obtained by flash column chromatography on silica gel. The KIE value of k_H/k_D= 1.3 was determined based on the product yield of **3a (**126 mg, 83%) and **9 (**99 mg, 64%).

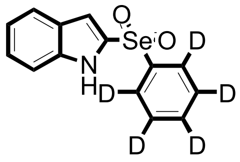

9

9 White solid, 99 mg, 64% yield, m.p. 159-161 °C;

^{1}H NMR (400 MHz, $CDCl_3$) δ 9.53 (s, 1H), 7.65 (d, *J* = 8.0 Hz, 1H), 7.53 (t, *J* = 7.3 Hz, 1H), 7.32-7.28 (m, 1H), 7.22 (s, 1H), 7.15 (t, *J* = 7.1 Hz, 1H);

^{13}C NMR (101 MHz, $CDCl_3$) δ 141.50, 137.51, 133.93, 133.56, 129.47, 127.32, 127.05, 126.14, 122.68, 121.62, 112.64, 109.39;

HRMS(ESI): m/z calcd for $C_{14}H_6D_5NNaO_2Se$ $(M+Na)^+$: 333.0161, found: 333.0162.

The *in situ* ESI-MS analysis MS Analysis

The model reaction mixture was picked and dissolved in methanol/toluene mixture (3:1, v/v) toluene when the reactant time 2 h, 4 h, 6 h, 8h, 10 h, prior to FT-ICR MS analysis. Each sample was analyzed three times by negative ion ESI FT-ICR MS. The mass range was set to 150-800 Da, and the instrument parameters were optimized for a mass range of 200-500 Da in order to cover the most abundant acidic compound mass peaks measured here. The ion accumulation time was 0.6 s. A total of 40 continuous 4 M data FT-ICR transients were coded. Repeatability of the FT-ICR MS experiments was tested by comparing the relative concentrations of the ^{16}O2 class species for the three replicates of all samples. The relative standard deviation of the relative concentration for most O_2 class species was below 5% (except for some O_2 class species with very small relative concentration), indicating that results of the FT-ICR MS experiments were stable.

Analytical Datas

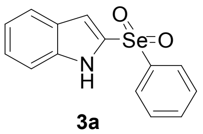

3a

2-Benzeneselenonyl-1H-indole (3a) White solid, 126.2 mg, 83% yield, m.p. 159-161 °C;

^{1}H NMR (400 MHz, $CDCl_3$) δ 9.53 (s, 1H), 8.02 (d, *J* = 7.7 Hz, 2H), 7.65 (d, *J* = 8.0 Hz, 1H), 7.53 (t, *J* = 7.3 Hz, 1H), 7.45 (dd, *J* = 18.0, 8.4 Hz, 3H), 7.32-7.28 (m, 1H), 7.22 (s, 1H), 7.15 (t, *J* = 7.1 Hz, 1H);

^{13}C NMR (101 MHz, $CDCl_3$) δ 141.50, 137.51, 133.93, 133.56, 129.47, 127.32, 127.05, 126.14, 122.68, 121.62, 112.64, 109.39;

HRMS(ESI): m/z calcd for $C_{14}H_{11}NNaO_2Se$ $(M+Na)^+$: 327.9847, found: 327.9844.

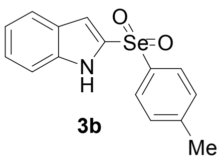

3b

2-(Toluene-4-selenonyl)-1H-indole (3b) White solid, 133.6 mg, 84% yield, m.p. 190-192 °C;

^{1}H NMR (400 MHz, $CDCl_3$) δ 8.91 (s, 1H), 7.88 (d, *J* = 8.4 Hz, 2H), 7.66 (d, *J* = 8.8 Hz, 1H), 7.43-7.39 (m, 1H), 7.36-7.27 (m, 3H), 7.20-7.14 (m, 2H), 2.39 (s, 3H);

^{13}C NMR (101 MHz, $CDCl_3$) δ 144.68, 138.68, 137.12, 134.69, 130.12, 127.49, 126.10, 122.81, 121.69, 112.36, 108.98, 21.74;

HRMS(ESI): m/z calcd for $C_{15}H_{13}NNaO_2Se$ $(M+Na)^+$: 342.0004, found: 342.0001.

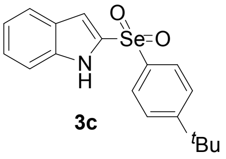

3c

2-(4-tert-Butyl-benzeneselenonyl)-1H-indole (3c) White solid, 156.7 mg, 87% yield, m.p. 192-194 °C;

^{1}H NMR (400 MHz, $CDCl_3$) δ 9.50 (s, 1H), 7.94 (d, *J* = 8.7 Hz, 2H), 7.65 (d, *J* = 8.1 Hz, 1H), 7.47 (d, *J* = 8.7 Hz, 2H), 7.42 (d, *J* = 7.7 Hz, 1H), 7.29 (ddd, *J* = 8.3, 7.1, 1.0 Hz, 1H), 7.21 (d, *J* = 1.3 Hz, 1H), 7.18-7.12 (m, 1H), 1.27 (s, 9H);

^{13}C NMR (101 MHz, $CDCl_3$) δ 157.57, 138.47, 137.38, 134.50, 127.28, 127.12, 126.54, 126.02, 122.67, 121.58, 112.59, 109.01, 35.33, 31.10;

HRMS(ESI): m/z calcd for $C_{18}H_{19}NNaO_2Se$ $(M+Na)^+$: 384.0473, found: 384.0470.

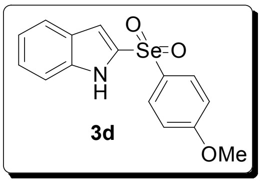

2-(4-Methoxy-benzeneselenonyl)-1H-indole (3d) White solid, 153.7 mg, 92% yield, m.p. 186-187 °C;

^{1}H NMR (400 MHz, $CDCl_3$) δ 8.25 (s, 1H), 7.55 (d, *J* = 8.0 Hz, 1H), 7.37 (d, *J* = 2.6 Hz, 1H), 7.32 (d, *J* = 8.1 Hz, 1H), 7.19-7.14 (m, 1H), 7.07 (ddd, *J* = 12.3, 7.3, 1.6 Hz, 3H), 6.69-6.62 (m, 2H), 3.65 (s, 3H);

^{13}C NMR (101 MHz, $CDCl_3$) δ 157.89, 136.57, 130.14, 129.62, 129.12, 128.68, 123.06, 120.89, 119.77, 114.61, 111.64, 104.72, 55.46;

HRMS(ESI): m/z calcd for $C_{15}H_{13}NNaO_3Se$ $(M+Na)^+$: 357.9953, found: 357.9950.

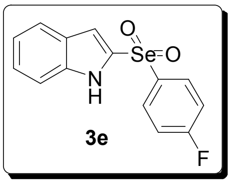

2-(4-Fluoro-benzeneselenonyl)-1H-indole (3e) White solid, 125.7 mg, 78% yield, m.p. 139-141 °C;

^{1}H NMR (400 MHz, $CDCl_3$) δ 9.12 (s, 1H), 8.02 (dd, *J* = 8.9, 5.0 Hz, 2H), 7.67 (d, *J* = 8.0 Hz, 1H), 7.42 (d, *J* = 7.8 Hz, 1H), 7.33 (d, *J* = 8.3 Hz, 1H), 7.20-7.15 (m, 4H);

^{13}C NMR (101 MHz, $CDCl_3$) δ 137.29, 133.98, 130.34, 130.24, 127.21, 126.39, 122.87, 121.88, 116.95, 116.72, 112.45, 109.48;

HRMS(ESI): m/z calcd for $C_{14}H_{10}FNNaO_2Se$ (M+Na)+: 345.9753, found: 345.9750.

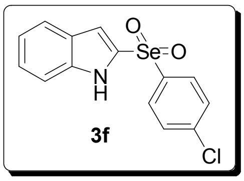

2-(4-Chloro-benzeneselenonyl)-1H-indole (3f) White solid, 137.1 mg, 81% yield, m.p. 146-148 °C;

^{1}H NMR (400 MHz, $CDCl_3$) δ 9.51 (s, 1H), 8.05 (d, *J* = 8.7 Hz, 2H), 7.50 (d, *J* = 8.8 Hz, 3H), 7.44-7.37 (m, 2H), 7.29-7.23 (m, 2H);

^{13}C NMR (101 MHz, $CDCl_3$) δ 140.67, 138.65, 135.98, 133.34, 131.34, 129.58, 129.38, 127.39, 123.24, 122.52, 112.51;

HRMS(ESI): m/z calcd for $C_{14}H_{10}ClNNaO_2Se$ $(M+Na)^+$: 361.9457, found: 361.9454.

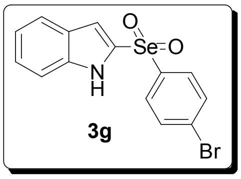

2-(4-Bromo-benzeneselenonyl)-1H-indole (3g) White solid, 159.0 mg, 83% yield, m.p. 191-193 °C;

^{1}H NMR (400 MHz, $CDCl_3$) δ 8.97 (s, 1H), 7.85 (d, *J* = 8.7 Hz, 2H), 7.67 (d, *J* = 8.1 Hz, 1H), 7.63 (d, *J* = 8.7 Hz, 2H), 7.42 (d, *J* = 8.4 Hz, 1H), 7.38-7.33 (m, 1H), 7.21-7.17 (m, 2H);

^{13}C NMR (101 MHz, $CDCl_3$) δ 140.67, 137.31, 133.63, 132.82, 131.77, 128.93, 127.25, 126.51, 122.92, 121.95, 112.42, 109.75;

HRMS(ESI): m/z calcd for $C_{14}H_{10}BrNNaO_2Se$ $(M+Na)^+$: 371.9664, found: 371.9661.

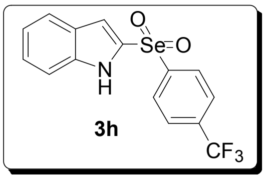

2-(4-Trifluoromethyl-benzeneselenonyl)-1H-indole (3h) White solid, 139.6 mg, 75% yield, m.p. 151-153 °C;

^{1}H NMR (400 MHz, $CDCl_3$) δ 8.96 (s, 1H), 8.12 (d, *J* = 8.2 Hz, 2H), 7.76 (d, *J* = 8.3 Hz, 2H), 7.68 (d, *J* = 8.1 Hz, 1H), 7.43 (d, *J* = 8.4 Hz, 1H), 7.39-7.35 (m, 1H), 7.24-7.08 (m, 2H);

^{13}C NMR (101 MHz, $CDCl_3$) δ 145.05, 137.35, 132.82, 127.81, 127.11, 126.61, 122.86, 121.94, 112.33, 110.25, 65.61;

HRMS(ESI): m/z calcd for $C_{15}H_{10}F_3NNaO_2Se$ (M+Na)+: 395.9721, found: 395.9718.

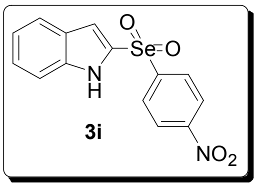

2-(4-Nitro-benzeneselenonyl)-1H-indole (3i) Light yellow solid, 120.5 mg, 69% yield, m.p. 129-132 °C;

^{1}H NMR (400 MHz, $CDCl_3$) δ 8.90 (s, 1H), 8.36-8.34 (m, 1H), 8.33 (d, *J* = 2.1 Hz, 1H), 8.20-8.15 (m, 2H), 7.69 (d, *J* = 8.1 Hz, 1H), 7.46-7.42 (m, 1H), 7.41-7.37 (m, 1H), 7.29 (dd, *J* = 2.1, 0.8 Hz, 1H), 7.22 (ddd, *J* = 8.0, 6.8, 1.1 Hz, 1H);

^{13}C NMR (101 MHz, $CDCl_3$) δ 150.40, 147.18, 137.49, 132.20, 129.24, 128.58, 127.15, 126.92, 124.61,

124.46, 122.97, 122.12, 112.35, 110.85;

HRMS(ESI): m/z calcd for $C_{14}H_{10}N_2NaO_4Se$ $(M+Na)^+$: 372.9698, found: 372.9695.

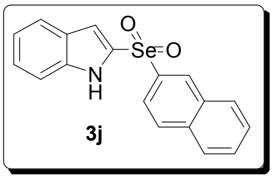

2-(Naphthalene-2-selenonyl)-1H-indole (3j) White solid, 139.9 mg, 79% yield, m.p. 156-158 °C;

^1H NMR (400 MHz, $CDCl_3$) δ 8.91 (s, 1H), 8.61 (s, 1H), 7.99-7.91 (m, 3H), 7.87 (d, *J* = 7.7 Hz, 1H), 7.68-7.58 (m, 3H), 7.49-7.29 (m, 3H), 7.17 (t, *J* = 7.5 Hz, 1H);

^{13}C NMR (101 MHz, $CDCl_3$) δ 138.24, 137.07, 135.14, 134.19, 132.18, 129.77, 129.44, 129.30, 128.74, 127.96, 127.74, 127.16, 126.11, 122.73, 122.27, 121.63, 112.22, 109.32.

HRMS(ESI): m/z calcd for $C_{18}H_{13}NNaO_2Se$ $(M+Na)^+$: 378.0004, found: 378.0001.

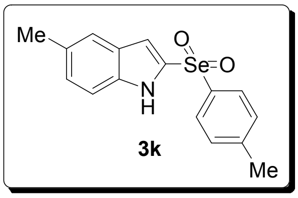

5-Methyl-2-(toluene-4-selenonyl)-1H-indole (3k) White solid, 124.6 mg, 75% yield, m.p. 135-137 °C;

^1H NMR (400 MHz, $CDCl_3$) δ 8.88 (s, 1H), 7.87 (d, *J* = 8.4 Hz, 2H), 7.42 (s, 1H), 7.29 (dd, *J* = 8.3, 5.3 Hz, 3H), 7.15 (dd, *J* = 8.5, 1.5 Hz, 1H), 7.09-7.07 (m, 1H), 2.41 (s, 3H), 2.38 (s, 3H);

^{13}CNMR (101 MHz, $CDCl_3$) δ 144.55, 138.80, 135.61, 134.43, 130.07, 128.03, 127.41, 121.98, 112.03, 108.53, 21.72;

HRMS(ESI): m/z calcd for $C_{16}H_{15}NNaO_2Se$ $(M+Na)^+$: 356.0160, found: 356.0157.

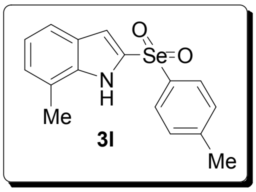

7-Methyl-2-(toluene-4-selenonyl)-1H-indole (3l) White solid, 126.3 mg, 76% yield, m.p. 171-173 °C;

^1H NMR (400 MHz, $CDCl_3$) δ 9.01 (s, 1H), 7.92 (t, *J* = 6.5 Hz, 2H), 7.49 (d, *J* = 7.7 Hz, 1H), 7.29 (d, *J* = 8.1 Hz, 2H), 7.18 (d, *J* = 2.2 Hz, 1H), 7.13-7.05 (m, 2H), 2.48 (s, 3H), 2.39 (s, 3H);

^{13}C NMR (101 MHz, $CDCl_3$) δ 144.61, 138.76, 137.21, 134.27, 130.11, 127.44, 126.30, 121.86, 120.26,

109.62, 21.72, 16.89;

HRMS(ESI): m/z calcd for $C_{16}H_{15}NNaO_2Se$ $(M+Na)^+$: 356.0160, found: 356.0157.

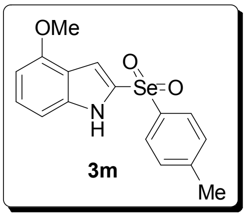

4-Methoxy-2-(toluene-4-selenonyl)-1H-indole (3m) White solid, 128.9 mg, 74% yield, m.p. 155-158 °C;

^1H NMR (400 MHz, $CDCl_3$) δ 8.90 (s, 1H), 7.87 (d, *J* = 8.4 Hz, 2H), 7.28 (d, *J* = 7.1 Hz, 3H), 7.23 (d, *J* = 8.0 Hz, 1H), 6.99 (d, *J* = 8.4 Hz, 1H), 6.52 (d, *J* = 7.8 Hz, 1H), 3.92 (s, 3H), 2.38 (s, 3H);

^{13}C NMR (101 MHz, $CDCl_3$) δ 154.65, 144.52, 130.06, 127.45, 127.20, 106.80, 105.11, 100.55, 55.52, 21.72;

HRMS(ESI): m/z calcd for $C_{16}H_{15}NNaO_3Se$ $(M)^+$: 372.0109, found: 372.0106.

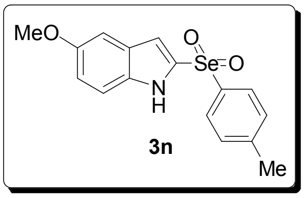

5-Methoxy-2-(toluene-4-selenonyl)-1H-indole (3n) White solid, 125.4 mg, 72% yield, m.p. 145-147 °C;

^1H NMR (400 MHz, $CDCl_3$) δ 8.92 (s, 1H), 7.87 (d, *J* = 8.4 Hz, 2H), 7.29 (dd, *J* = 8.5, 4.2 Hz, 3H), 7.08 (dd, *J* = 2.1, 0.9 Hz, 1H), 7.04-6.97 (m, 2H), 3.82 (s, 3H), 2.39 (s, 3H);

^{13}C NMR (101 MHz, $CDCl_3$) δ 155.26, 144.58, 134.74, 132.48, 130.09, 127.73, 127.41, 117.75, 113.35, 108.52, 102.66, 55.83, 21.73;

HRMS(ESI): m/z calcd for $C_{16}H_{15}NNaO_3Se$ (M+Na)+: 372.0109, found: 372.0106.

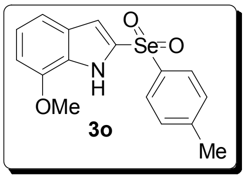

7-Methoxy-2-(toluene-4-selenonyl)-1H-indole (3o) White solid, 116.7 mg, 67% yield, m.p.149-151 °C;

^1H NMR (400 MHz, $CDCl_3$) δ 9.00 (s, 1H), 7.86 (d, *J* = 8.3 Hz, 2H), 7.28 (s, 2H), 7.23 (d, *J* = 8.2 Hz, 1H),

7.16–7.03 (m, 3H), 6.73 (d, *J* = 7.7 Hz, 1H), 3.95 (s, 3H), 2.38 (s, 3H);

^{13}C NMR (101 MHz, $CDCl_3$) δ 146.65, 144.52, 138.77, 134.40, 130.04, 127.47, 122.15, 114.85, 109.09, 104.80, 55.60, 21.71;

HRMS(ESI): m/z calcd for $C_{16}H_{15}NNaO_3Se$ (M+Na)+: 372.0109, found: 372.0106.

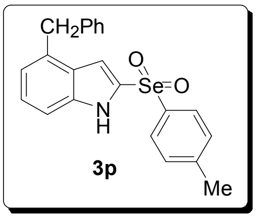

4-Benzyl-2-(toluene-4-selenonyl)-1H-indole (3p) White solid, 134.8 mg, 66% yield, m.p. 136-139 °C;

^{1}H NMR (400 MHz, $CDCl_3$) δ 8.97 (s, 1H), 7.87 (d, *J* = 8.3 Hz, 2H), 7.47 (d, *J* = 7.2 Hz, 2H), 7.37 (dt, *J* = 24.0, 7.0 Hz, 4H), 7.28 (s, 1H), 7.22 (t, *J* = 8.1 Hz, 1H), 6.58 (d, *J* = 7.8 Hz, 1H), 5.18 (s, 2H), 2.38 (s, 3H);

^{13}C NMR (101 MHz, $CDCl_3$) δ 153.74, 138.79, 138.54, 136.92, 133.16, 130.07, 128.72, 128.15, 127.44, 107.00, 105.38, 101.85, 70.11, 21.72;

HRMS(ESI): m/z calcd for $C_{22}H_{19}NNaO_2Se$ $(M)^+$: 432.0473, found: 432.0470.

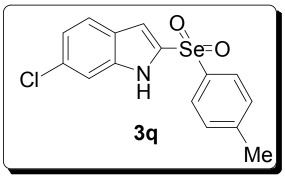

6-Bromo-2-(toluene-4-selenonyl)-1H-indole (3q) Brown solid, 158.7 mg, 90% yield, m.p. 181-182 °C;

^{1}H NMR (400 MHz, $CDCl_3$) δ 9.25 (s, 1H), 7.88 (d, *J* = 8.3 Hz, 2H), 7.61-7.48 (m, 2H), 7.33-7.27 (m, 3H), 2.39 (s, 3H);

^{13}C NMR (101 MHz, $CDCl_3$) δ 144.99, 138.28, 137.75, 135.32, 130.24, 127.50, 126.01, 125.30, 123.96, 119.78, 115.35, 108.91, 21.77;

HRMS(ESI): m/z calcd for $C_{15}H_{12}ClNNaO_2Se$ $(M+Na)^+$: 375.9614, found: 375.9611.

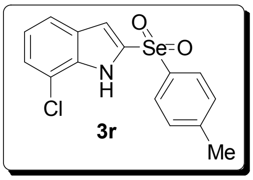

7-Bromo-2-(toluene-4-selenonyl)-1H-indole (3r) Dark red solid, 160.5 mg, 91% yield, m.p. 155-158

°C;

^{1}H NMR (400 MHz, $CDCl_3$) δ 8.86 (s, 1H), 7.91 (d, *J* = 8.3 Hz, 2H), 7.60 (d, *J* = 8.1 Hz, 1H), 7.51-7.47 (m, 1H), 7.33 (d, *J* = 8.1 Hz, 2H), 7.22 (d, *J* = 2.2 Hz, 1H), 7.06 (t, *J* = 7.8 Hz, 1H), 2.41 (s, 3H);

^{13}C NMR (101 MHz, $CDCl_3$) δ 144.87, 138.14, 135.77, 135.57, 130.10, 128.20, 128.02, 127.51, 122.67, 121.87, 109.60, 105.32, 21.64;

HRMS(ESI): m/z calcd for $C_{15}H_{12}ClNNaO_2Se$ $(M+Na)^+$: 375.9614, found: 375.9611.

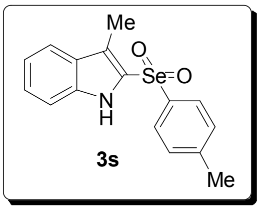

3s

3-Methyl-2-(toluene-4-selenonyl)-1H-indole (3s) Yellow solid, 108.0 mg, 65% yield, m.p. 186-187 °C;

^{1}H NMR (400 MHz, $CDCl_3$) δ 9.28 (s, 1H), 7.87 (dd, *J* = 8.3, 2.6 Hz, 2H), 7.58 (d, *J* = 8.1 Hz, 1H), 7.39 (d, *J* = 8.4 Hz, 1H), 7.33-7.23 (m, 3H), 7.17-7.11 (m, 1H), 2.53 (s, 3H), 2.36 (s, 3H);

^{13}C NMR (101 MHz, $CDCl_3$) δ 144.38, 139.09, 136.09, 130.02, 129.52, 128.32, 126.12, 118.51, 112.42, 21.65, 9.02;

HRMS(ESI): m/z calcd for $C_{16}H_{15}NNaO_2Se$ $(M+Na)^+$: 356.0160, found: 356.0157.

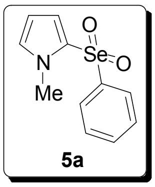

5a

2-Benzeneselenonyl-1-methyl-1H-pyrrole (5a) Light yellow viscous solid, 101.9 mg, 76% yield, m.p. 76-78 ^{o}C;

^{1}H NMR (400 MHz, $CDCl_3$) δ 7.88 (d, *J* = 7.1 Hz, 2H), 7.57-7.48 (m, 3H), 7.03 (dd, *J* = 4.0, 1.9 Hz, 1H), 6.76 (s, 1H), 6.17 (dd, *J* = 4.0, 2.6 Hz, 1H), 3.70 (s, 3H);

^{13}C NMR (101 MHz, $CDCl_3$) δ 142.25, 132.98, 129.81, 129.31, 127.23, 118.96, 108.45, 35.73;

HRMS(ESI): m/z calcd for $C_{11}H_{11}NNaO_2Se$ $(M+H)^+$: 291.9847, found: 291.9844.

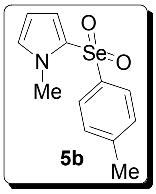

5b

1-Methyl-2-(toluene-4-selenonyl)-1H-pyrrole (5b) White solid, 111.5 mg, 79% yield, m.p. 99-101 °C;

^{1}H NMR (400 MHz, $CDCl_3$) δ 7.77 (d, *J* = 8.3 Hz, 2H), 7.29 (d, *J* = 8.0 Hz, 2H), 7.00 (dd, *J* = 4.0, 1.9 Hz, 1H), 6.74 (t, *J* = 2.2 Hz, 1H), 6.16 (dd, *J* = 4.0, 2.6 Hz, 1H), 3.70 (s, 3H), 2.41 (s, 3H);

^{13}C NMR (101 MHz, $CDCl_3$) δ 143.90, 139.36, 129.94, 129.54, 128.44, 118.63, 108.35, 35.72, 21.68;

HRMS(ESI): m/z calcd for $C_{12}H_{13}NNaO_2Se$ $(M+H)^+$: 306.0009, found: 306.0006.

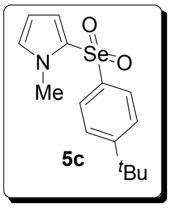

2-(4-tert-Butyl-benzeneselenonyl)-1-methyl-1H-pyrrole (5c) Pale yellow solid, 126.5 mg, 78% yield, m.p. 59-61 °C;

^{1}H NMR (400 MHz, $CDCl_3$) δ 7.80 (d, *J* = 8.7 Hz, 2H), 7.50 (d, *J* = 8.7 Hz, 2H), 7.02 (dd, *J* = 4.0, 1.9 Hz, 1H), 6.75 (t, *J* = 2.2 Hz, 1H), 6.16 (dd, *J* = 4.0, 2.6 Hz, 1H), 3.72 (s, 3H), 1.32 (s, 9H);

^{13}C NMR (101 MHz, $CDCl_3$) δ 156.84, 139.25, 129.56, 128.43, 127.16, 126.33, 118.64, 108.33, 35.79, 35.30, 31.18;

HRMS(ESI): m/z calcd for $C_{15}H_{19}NNaO_2Se$ $(M+H)^+$: 348.0479, found: 348.0476.

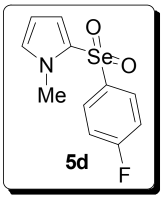

2-(4-Fluoro-benzeneselenonyl)-1-methyl-1H-pyrrole (5d) Pale yellow solid, 98.7 mg, 69% yield, m.p. 61-63 °C;

^{1}H NMR (400 MHz, $CDCl_3$) δ 7.90 (dd, *J* = 8.9, 5.1 Hz, 2H), 7.17 (t, *J* = 8.6 Hz, 2H), 7.01 (dd, *J* = 4.0, 1.9 Hz, 1H), 6.78 (t, *J* = 2.1 Hz, 1H), 6.17 (dd, *J* = 4.0, 2.6 Hz, 1H), 3.71 (s, 3H);

^{13}C NMR (101 MHz, $CDCl_3$) δ 166.49, 163.95, 138.35, 130.07, 129.97, 127.72, 118.97, 116.67, 116.44, 108.52, 35.69;

HRMS(ESI): m/z calcd for $C_{11}H_{10}FNNaO_2Se$ $(M+H)^+$: 309.9758, found: 309.9755.

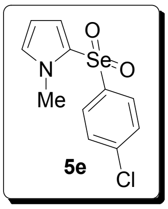

2-(4-Chloro-benzeneselenonyl)-1-methyl-1H-pyrrole (5e) Yellow solid, 98.3 mg, 65% yield, m.p. 73-75 ^{o}C;

^{1}H NMR (400 MHz, $CDCl_3$) δ 7.82 (d, *J* = 8.8 Hz, 2H), 7.47 (d, *J* = 8.8 Hz, 2H), 7.03 (dd, *J* = 4.1, 1.9 Hz, 1H), 6.78 (t, *J* = 2.2 Hz, 1H), 6.18 (dd, *J* = 4.1, 2.6 Hz, 1H), 3.71 (s, 3H);

^{13}C NMR (101 MHz, $CDCl_3$) δ 140.87, 139.57, 130.13, 129.65, 128.77, 127.54, 119.30, 108.70, 35.80;

HRMS(ESI): m/z calcd for $C_{11}H_{10}ClNNaO_2Se$ $(M+H)^+$: 325.9463, found: 325.9460.

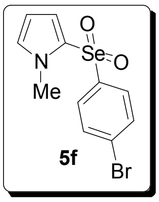

2-(4-Bromo-benzeneselenonyl)-1-methyl-1H-pyrrole (5f) Pale yellow solid, 114.5 mg, 66% yield, m.p. 90-92 ^{o}C;

^{1}H NMR (400 MHz, $CDCl_3$) δ 7.74 (d, *J* = 8.7 Hz, 2H), 7.64 (d, *J* = 8.7 Hz, 2H), 7.03 (dd, *J* = 4.1, 1.9 Hz, 1H), 6.78 (t, *J* = 2.2 Hz, 1H), 6.18 (dd, *J* = 4.1, 2.6 Hz, 1H), 3.71 (s, 3H);

^{13}C NMR (101 MHz, $CDCl_3$) δ 141.39, 132.62, 130.16, 128.83, 128.07, 127.45, 119.32, 35.79;

HRMS(ESI): m/z calcd for $C_{11}H_{10}BrNNaO_2Se$ $(M+H)^+$: 369.8958, found: 369.8955.

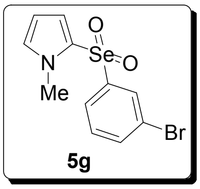

2-(3-Bromo-benzeneselenonyl)-1-methyl-1H-pyrrole (5g) Pale yellow solid, 118.0 mg, 68% yield, m.p. 66-68 ^{o}C;

^{1}H NMR (400 MHz, $CDCl_3$) δ 8.01 (d, *J* = 8.2 Hz, 2H), 7.77 (d, *J* = 8.3 Hz, 2H), 7.09 (dd, *J* = 4.1, 1.9 Hz, 1H), 6.81 (t, *J* = 2.2 Hz, 1H), 6.21 (dd, *J* = 4.1, 2.6 Hz, 1H), 3.73 (s, 3H);

^{13}C NMR (101 MHz, $CDCl_3$) δ 145.96, 134.76, 134.43, 130.61, 127.77, 126.85, 126.53, 126.50, 124.63, 121.92, 119.92, 108.94, 35.87;

HRMS(ESI): m/z calcd for $C_{11}H_{10}BrNNaO_2Se$ $(M+H)^+$: 369.8958, found: 369.8955.

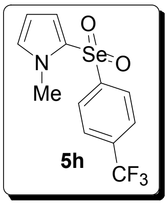

1-Methyl-2-(4-trifluoromethyl-benzeneselenonyl)-1H-pyrrole (5h) Pale yellow solid, 99.2 mg, 59% yield, m.p. 55-58 °C;

^{1}H NMR (400 MHz, $CDCl_3$) δ 8.01 (t, *J* = 1.8 Hz, 1H), 7.81 (d, *J* = 7.9 Hz, 1H), 7.68 (d, *J* = 8.0 Hz, 1H), 7.38 (t, *J* = 7.9 Hz, 1H), 7.05 (dd, *J* = 4.1, 1.9 Hz, 1H), 6.80 (t, *J* = 2.2 Hz, 1H), 6.20 (dd, *J* = 4.1, 2.6 Hz, 1H), 3.72 (s, 3H);

^{13}C NMR (101 MHz, $CDCl_3$) δ 144.26, 136.02, 130.86, 130.36, 130.09, 127.11, 125.78, 123.25, 119.62, 108.77, 35.85;

HRMS(ESI): m/z calcd for $C_{12}H_{10}F_3NNaO_2Se$ (M)+: 359.9726, found: 359.9723.

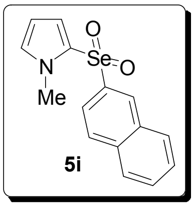

1-Methyl-2-(naphthalene-2-selenonyl)-1H-pyrrole (5i) Pale yellow solid, 111.3 mg, 70% yield, m.p. 60-63 °C;

^{1}H NMR (400 MHz, $CDCl_3$) δ 8.50 (s, 1H), 7.93 (td, *J* = 17.0, 16.6, 7.8 Hz, 3H), 7.80 (dd, *J* = 8.7, 1.9 Hz, 1H), 7.66-7.57 (m, 2H), 7.10 (dd, *J* = 4.0, 1.9 Hz, 1H), 6.75 (t, *J* = 2.2 Hz, 1H), 6.19 (dd, *J*= 4.0, 2.6 Hz, 1H), 3.72 (s, 3H);

^{13}C NMR (101 MHz, $CDCl_3$) δ 139.13, 135.02, 132.30, 129.85, 129.73, 129.47, 129.15, 128.33, 128.06, 127.72, 122.71, 119.14, 108.54, 35.81;

HRMS(ESI): m/z calcd for $C_{15}H_{13}NNaO_2Se$ $(M+H)^+$: 342.0009, found: 342.0006.

Spectrums

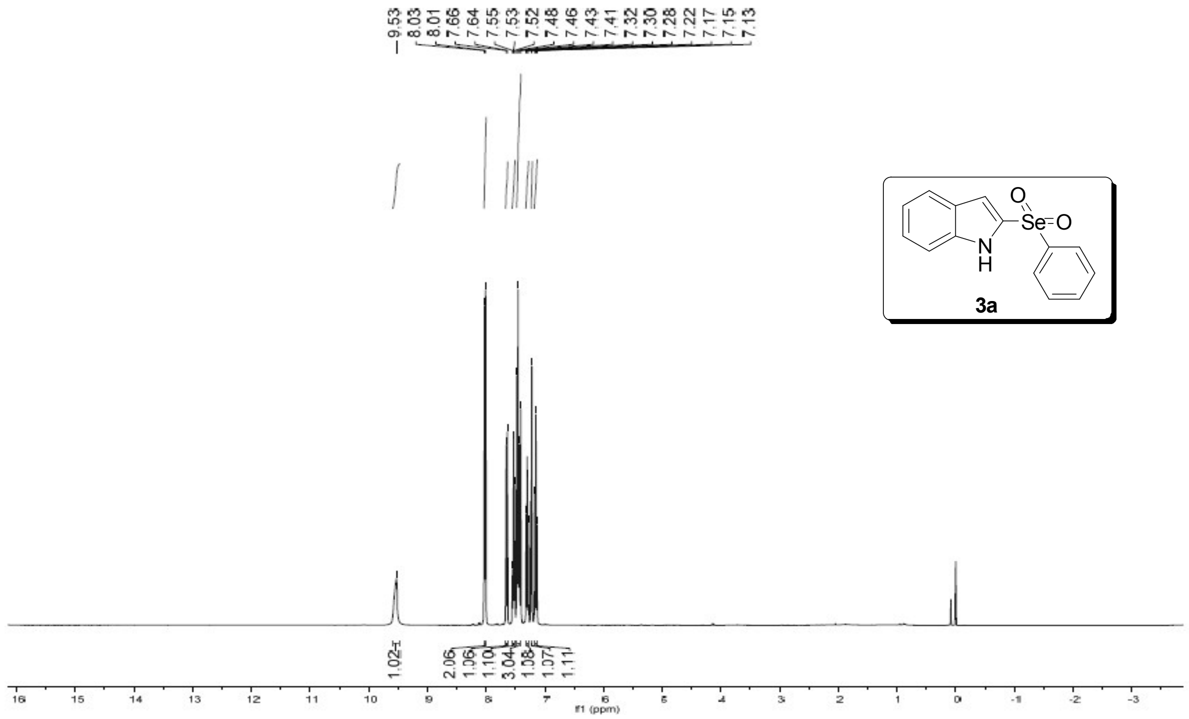

Figure 1. ^{1}H NMR **3a**

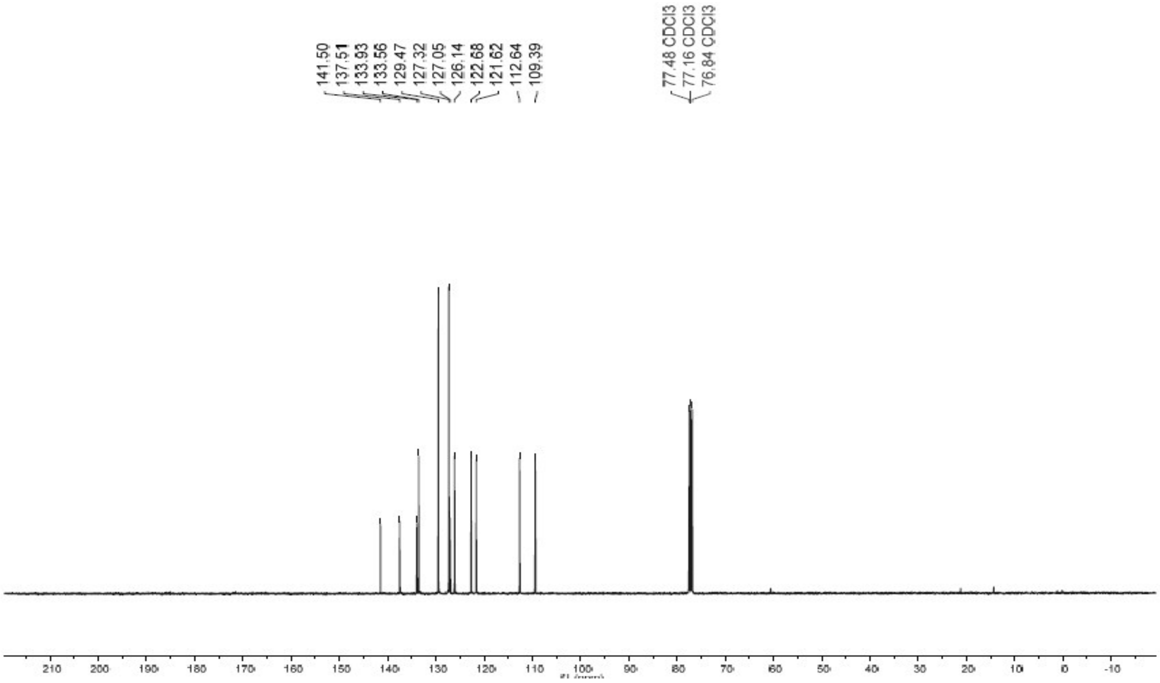

Figure 2. ^{13}C NMR **3a**

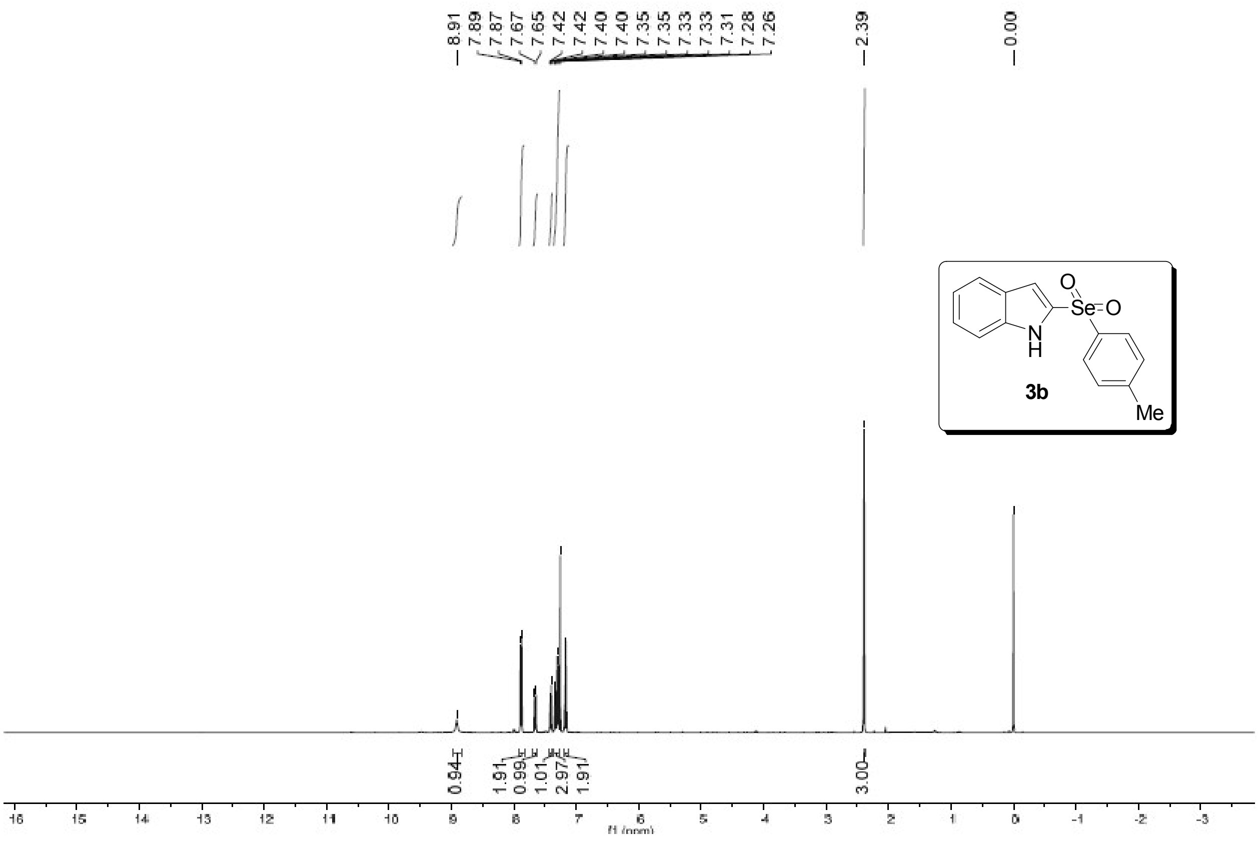

Figure 3. ^{1}H NMR **3b**

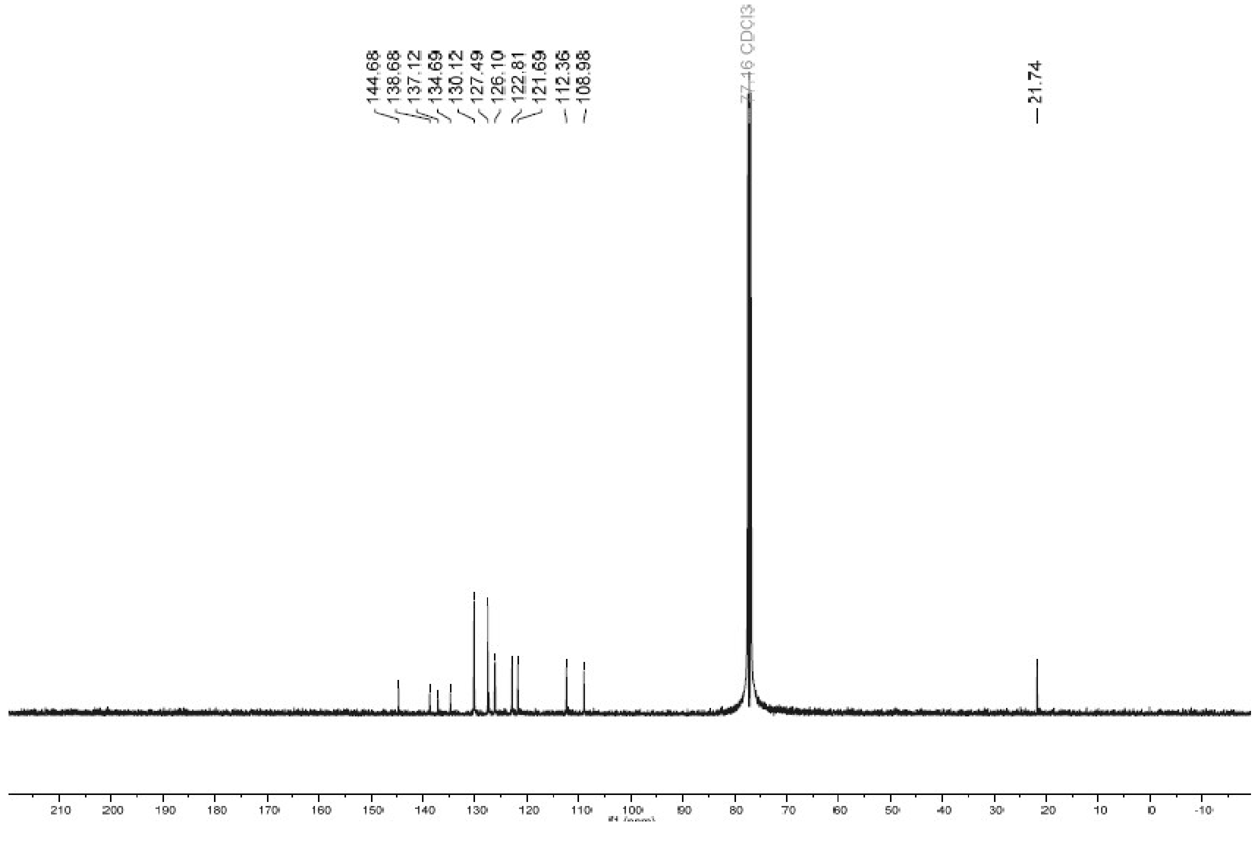

Figure 4. ^{13}C NMR **3b**

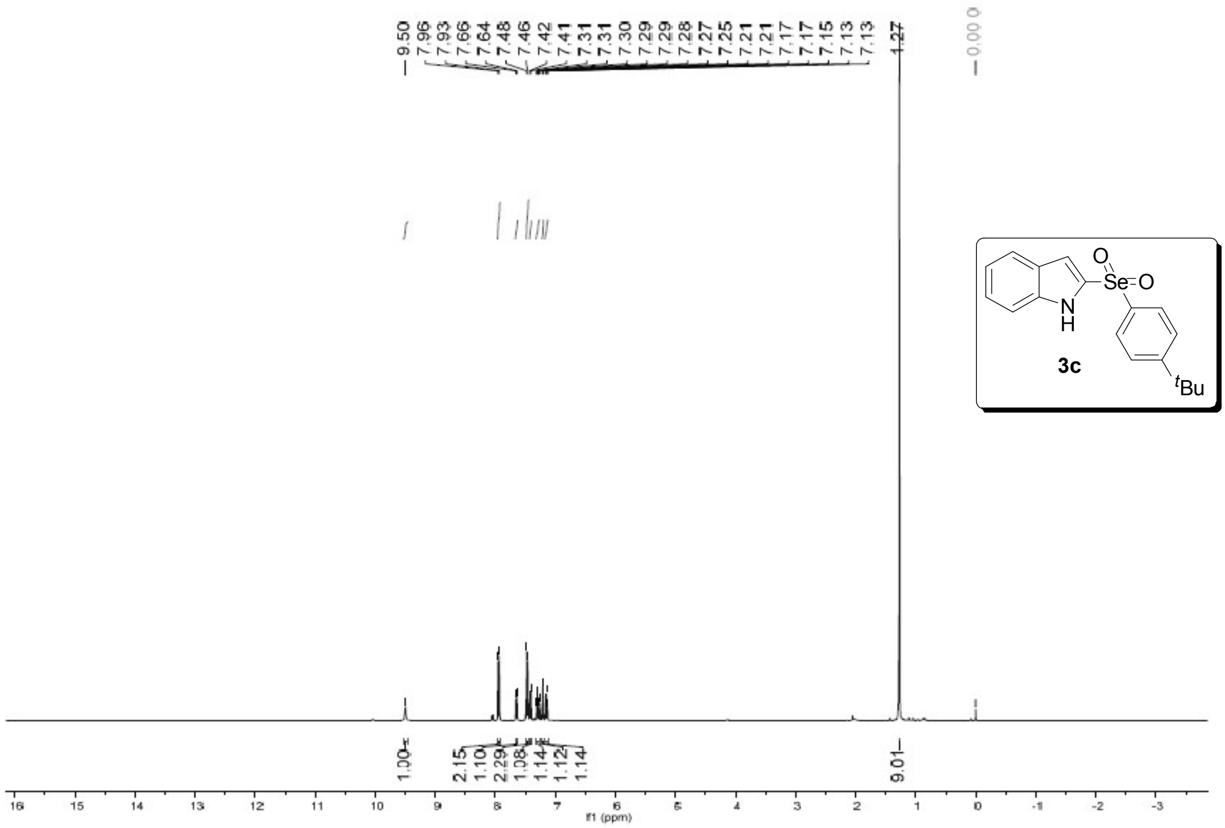

Figure 5. ¹H NMR **3c**

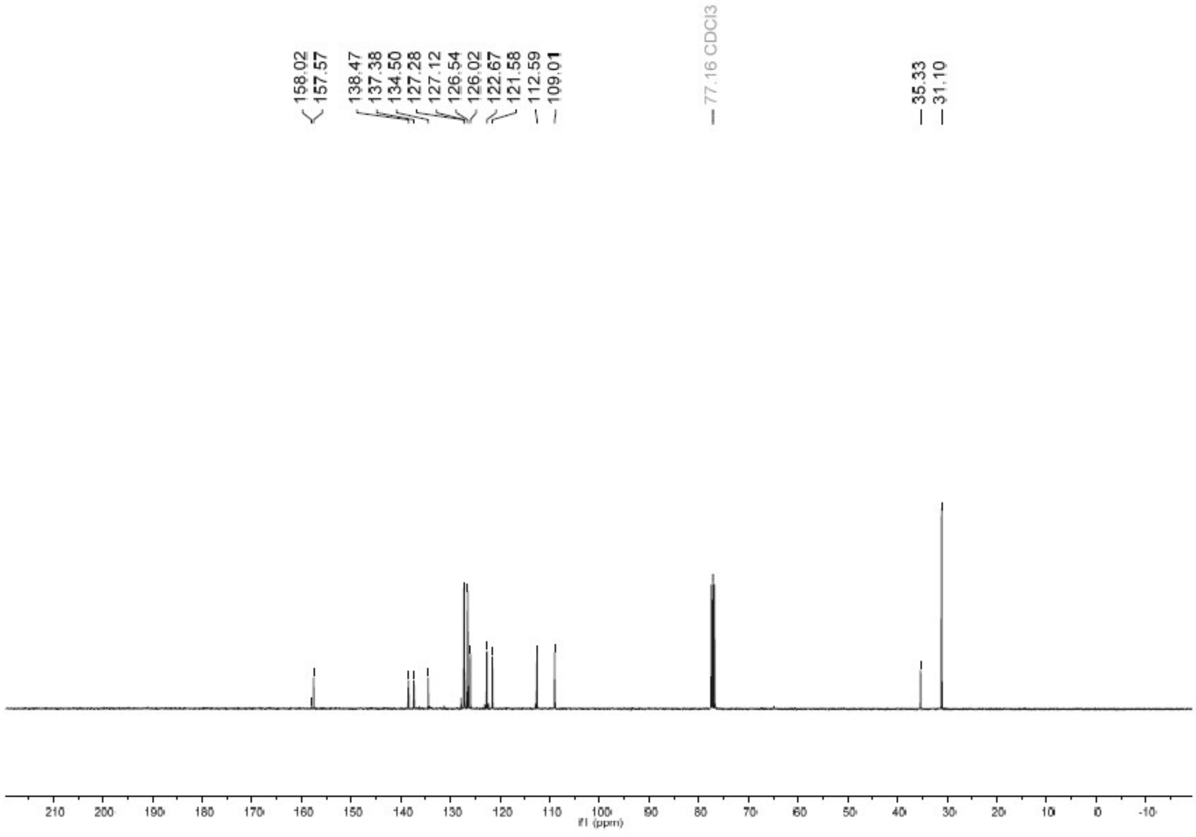

Figure 6. ¹³C NMR **3c**

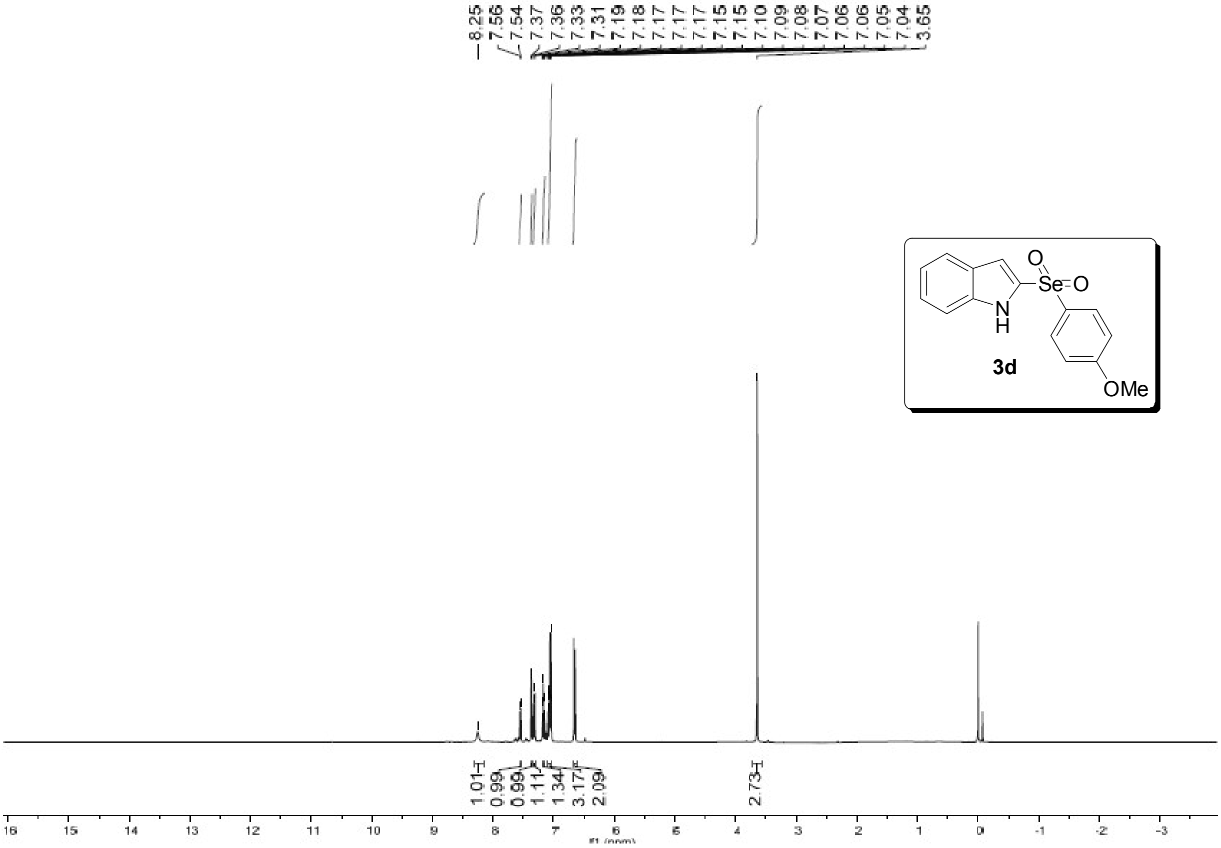

Figure 7. ^{1}H NMR **3d**

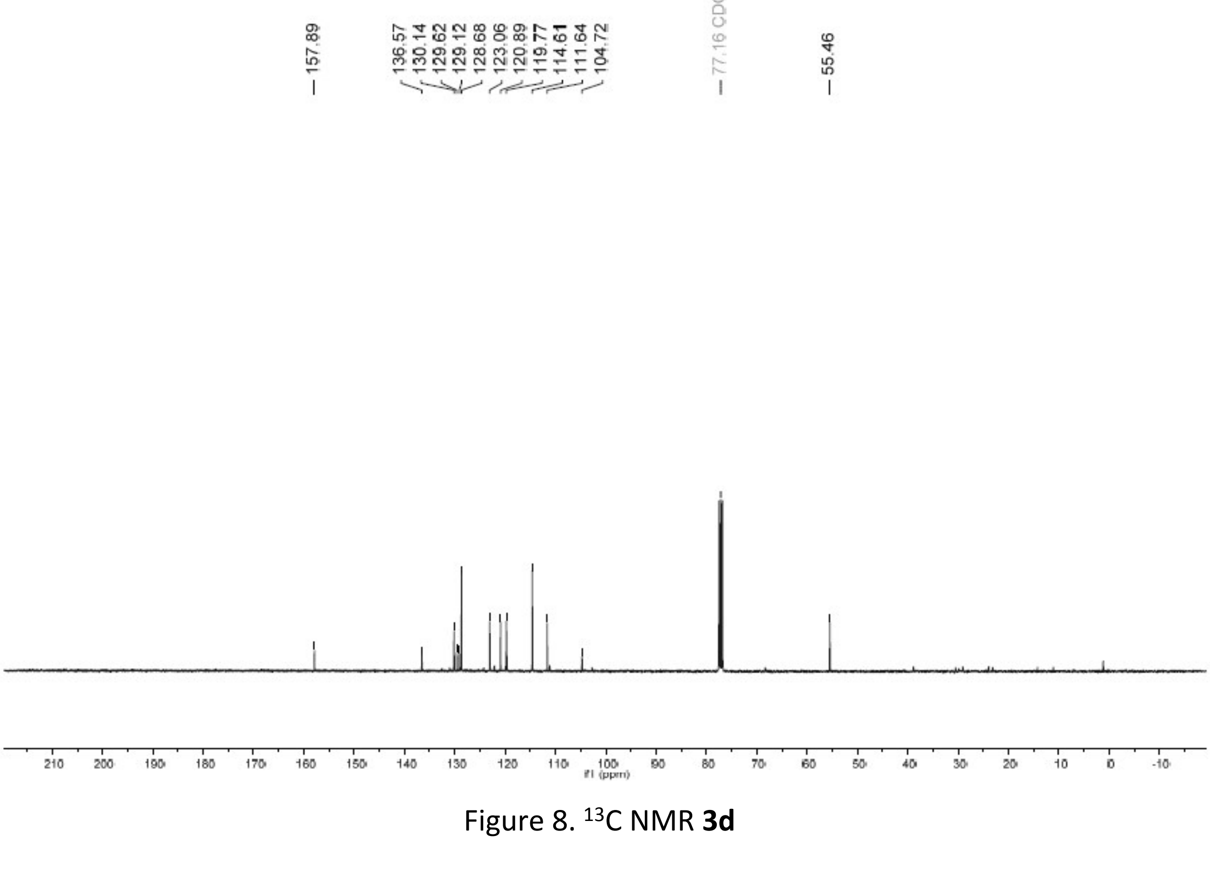

Figure 8. ^{13}C NMR **3d**

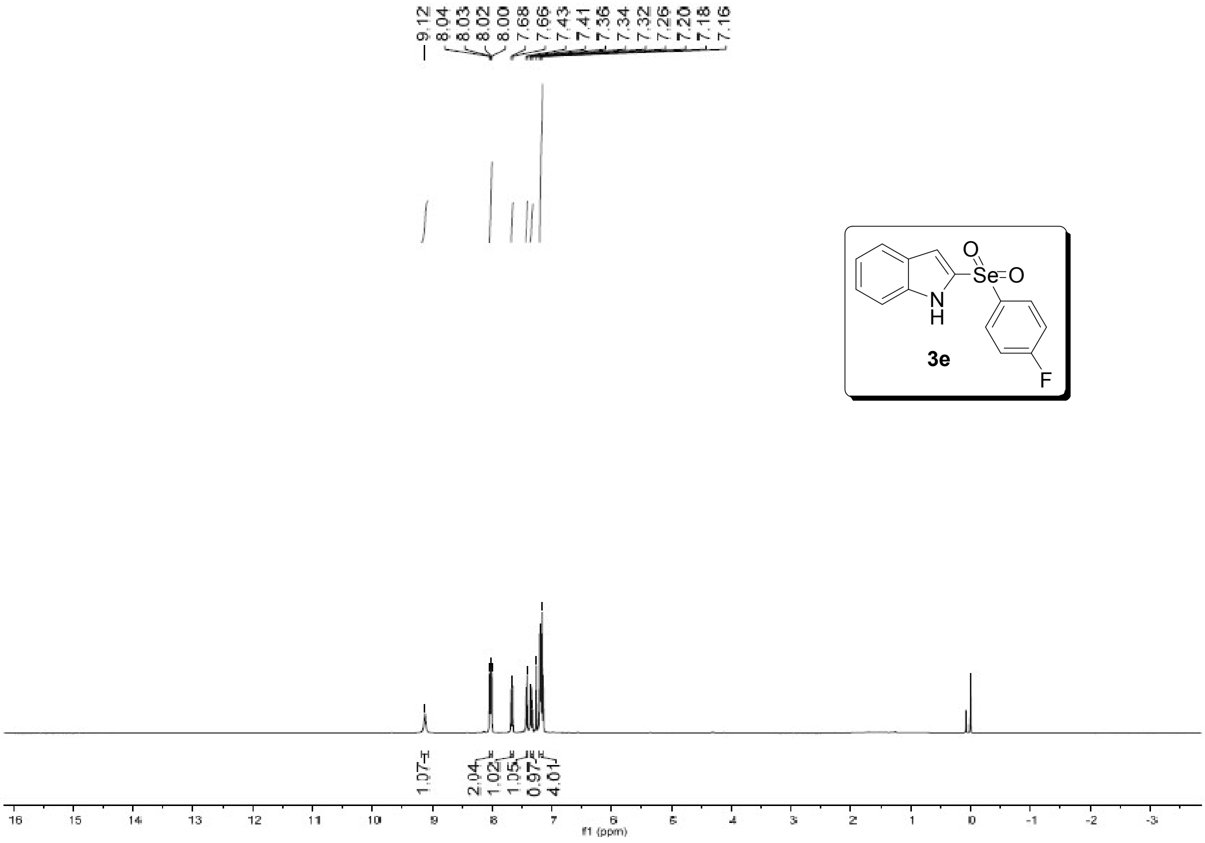

Figure 9. ^{1}H NMR **3e**

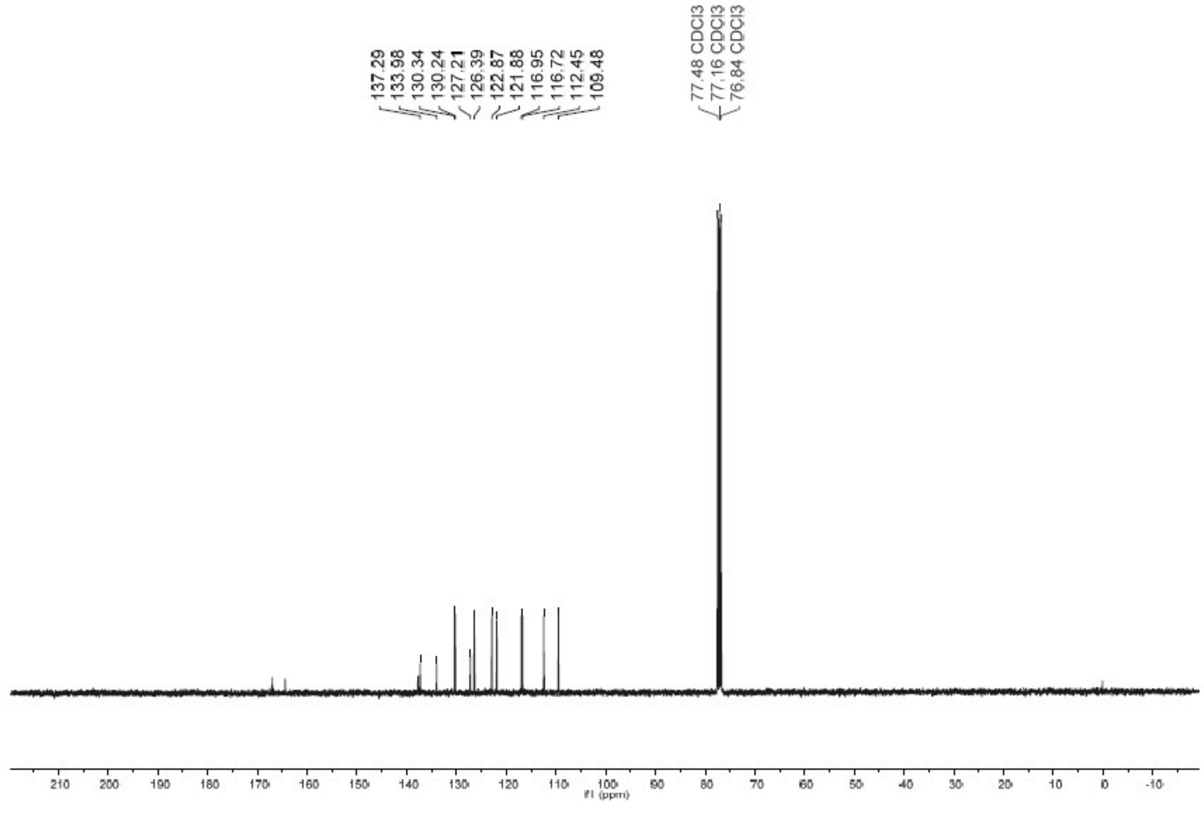

Figure 10. ^{13}C NMR **3e**

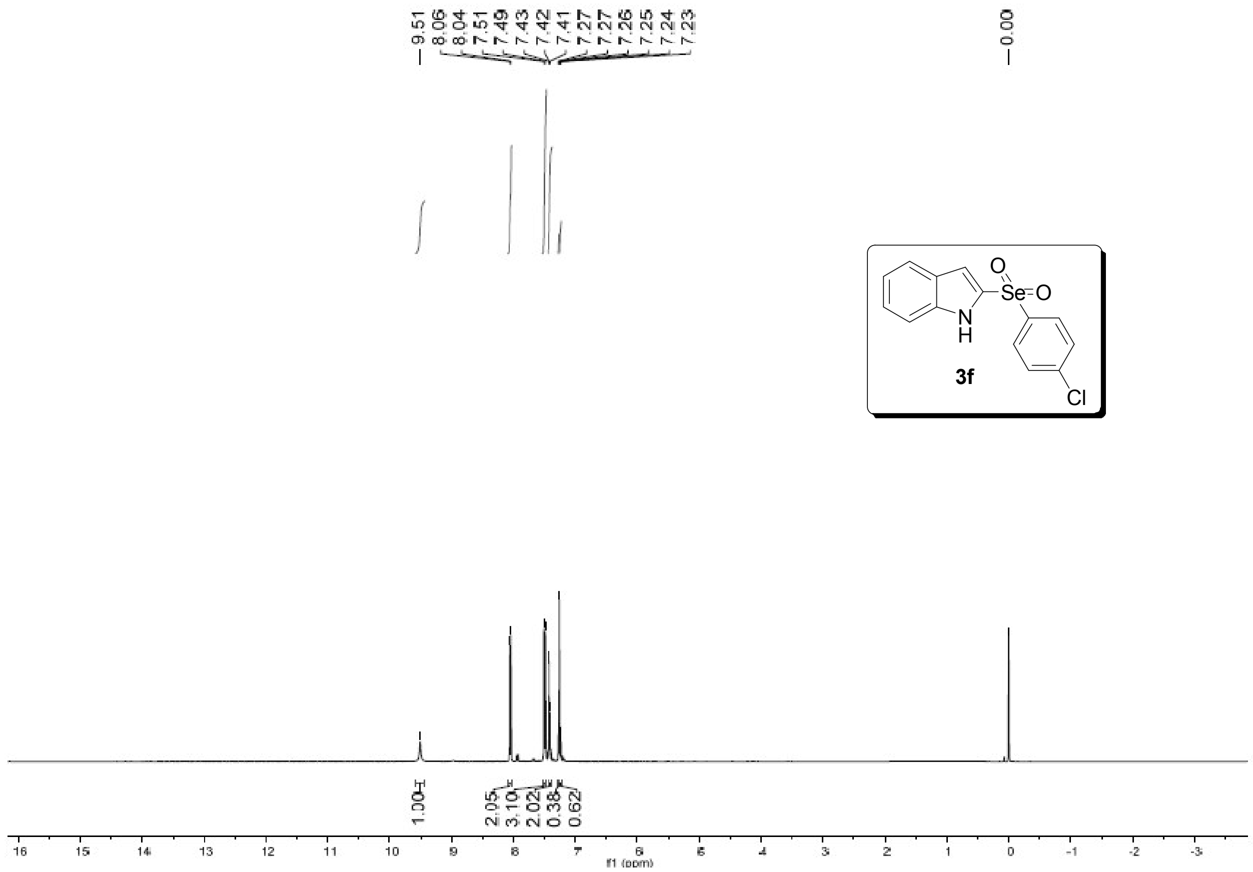

Figure 11. ^{1}H NMR **3f**

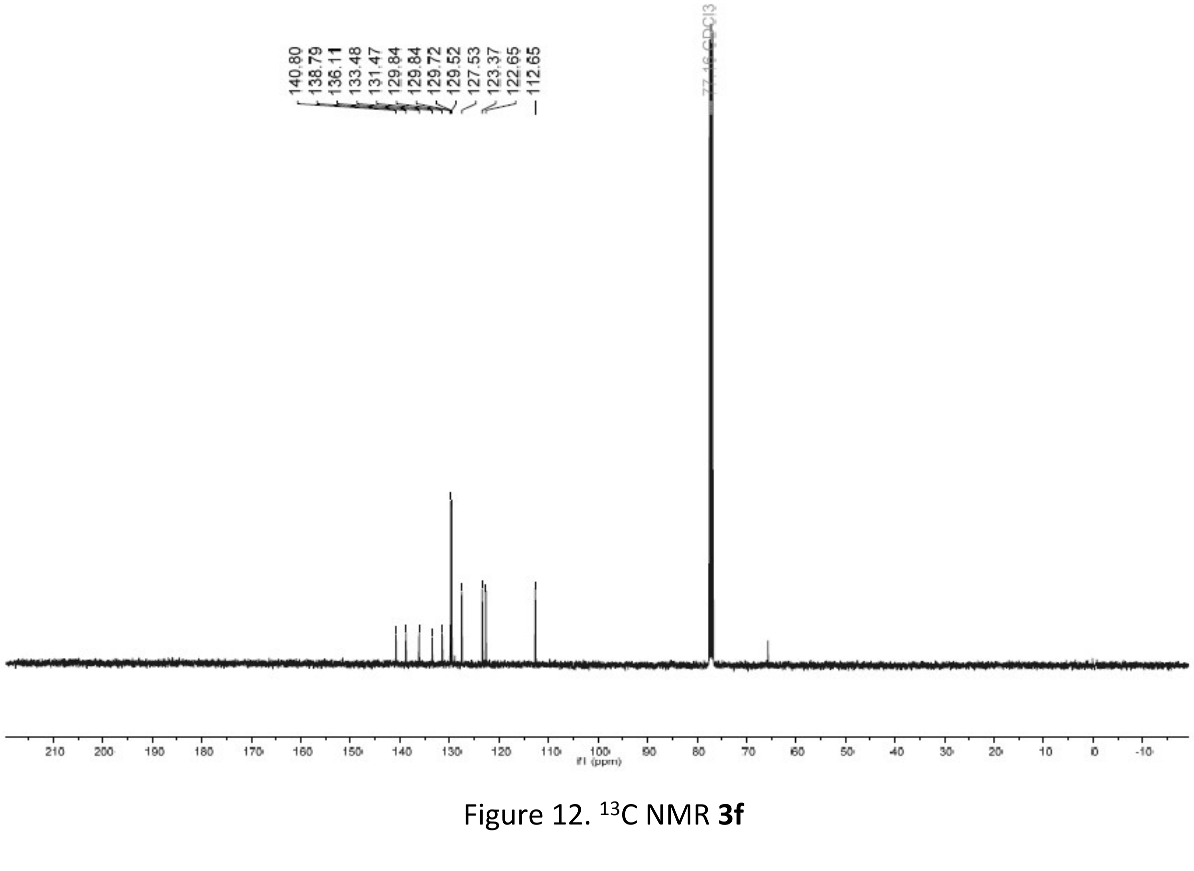

Figure 12. ^{13}C NMR **3f**

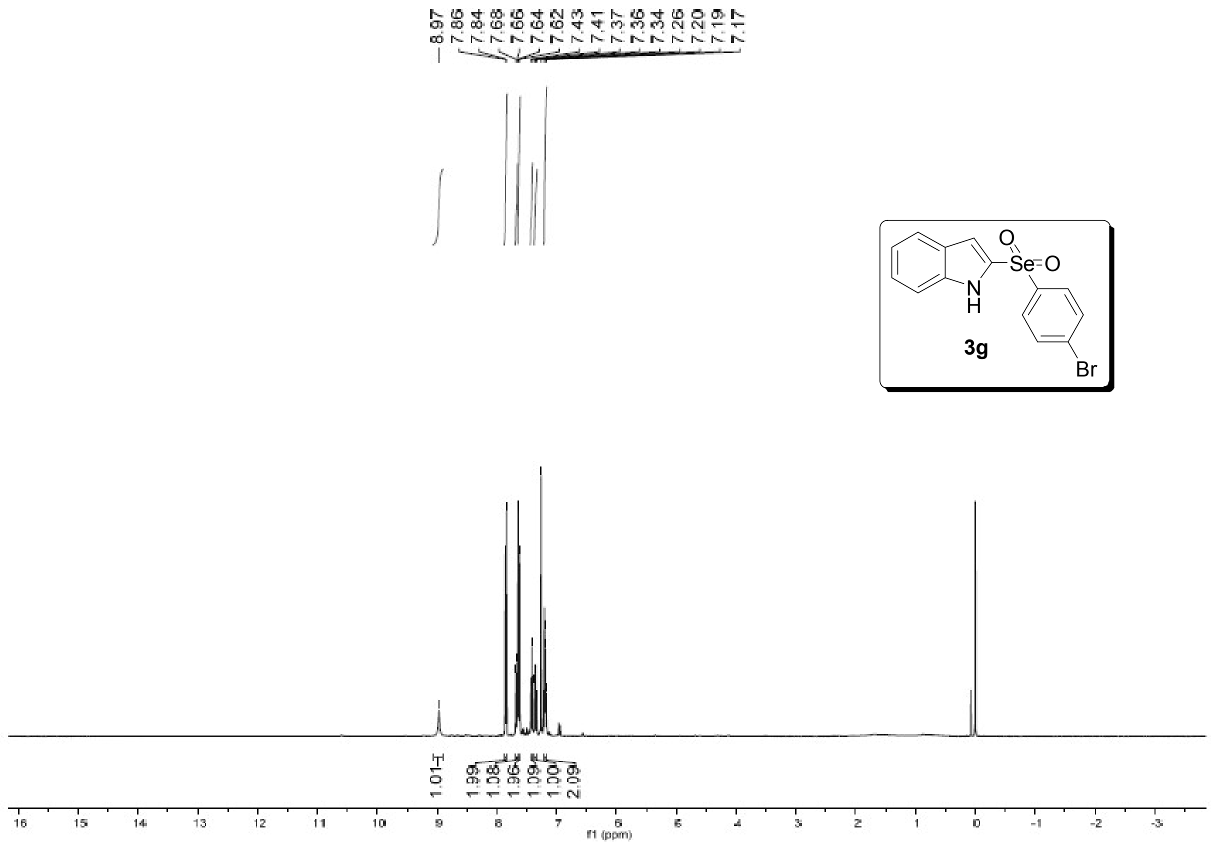

Figure 13. ^{1}H NMR **3g**

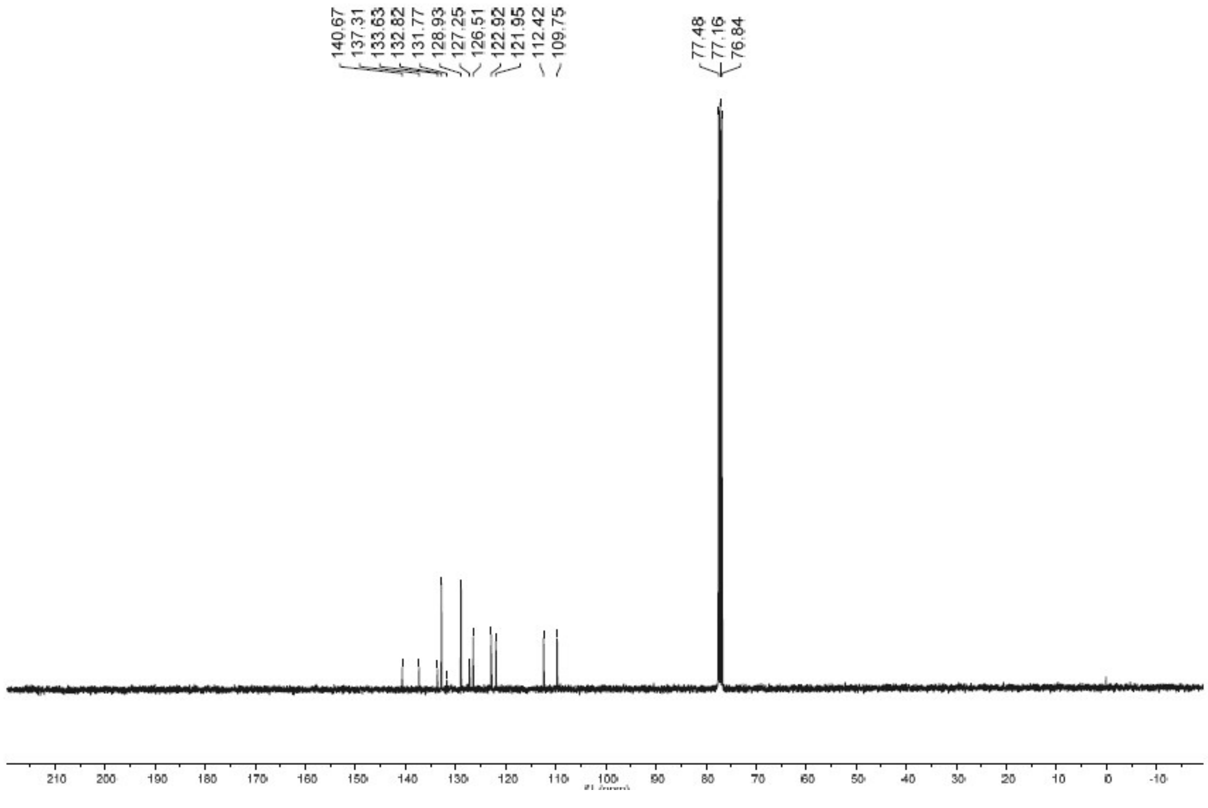

Figure 14. ^{13}C NMR **3g**

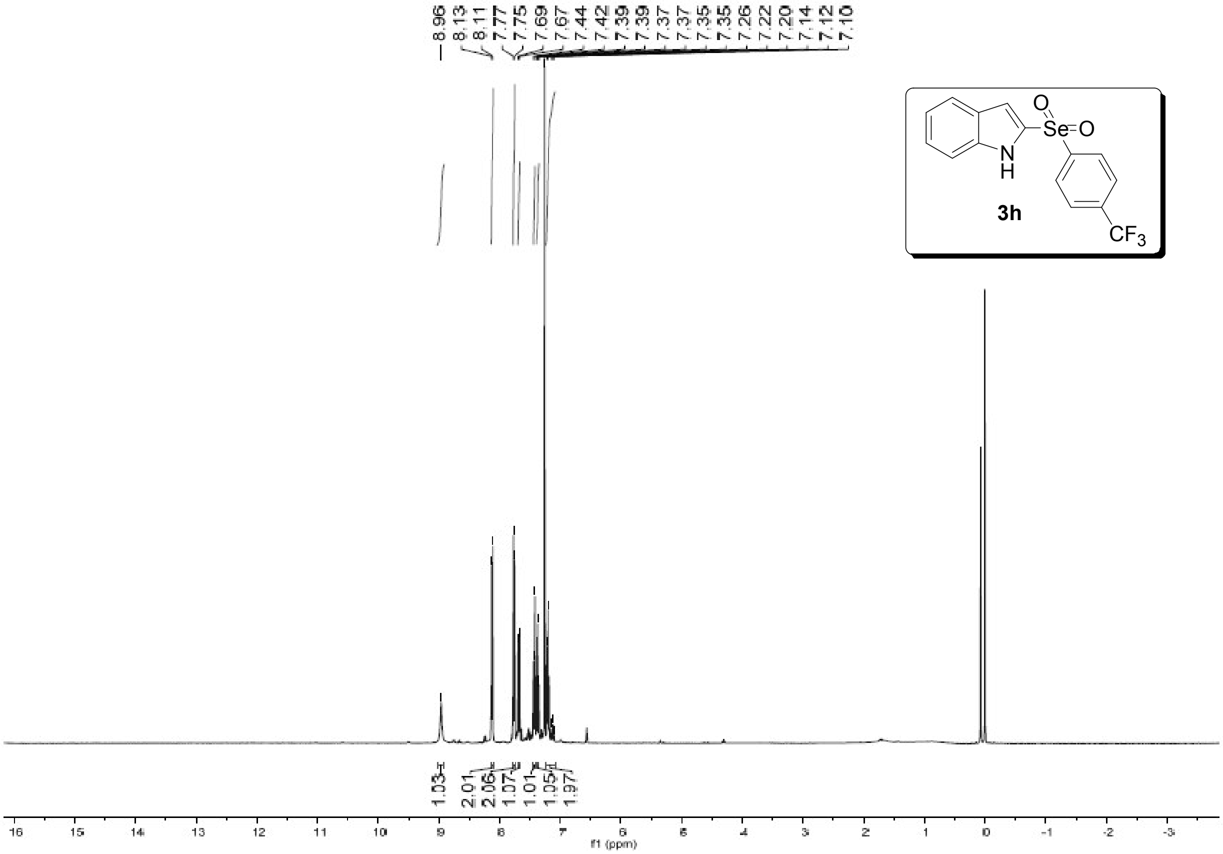

Figure 15. ^{1}H NMR **3h**

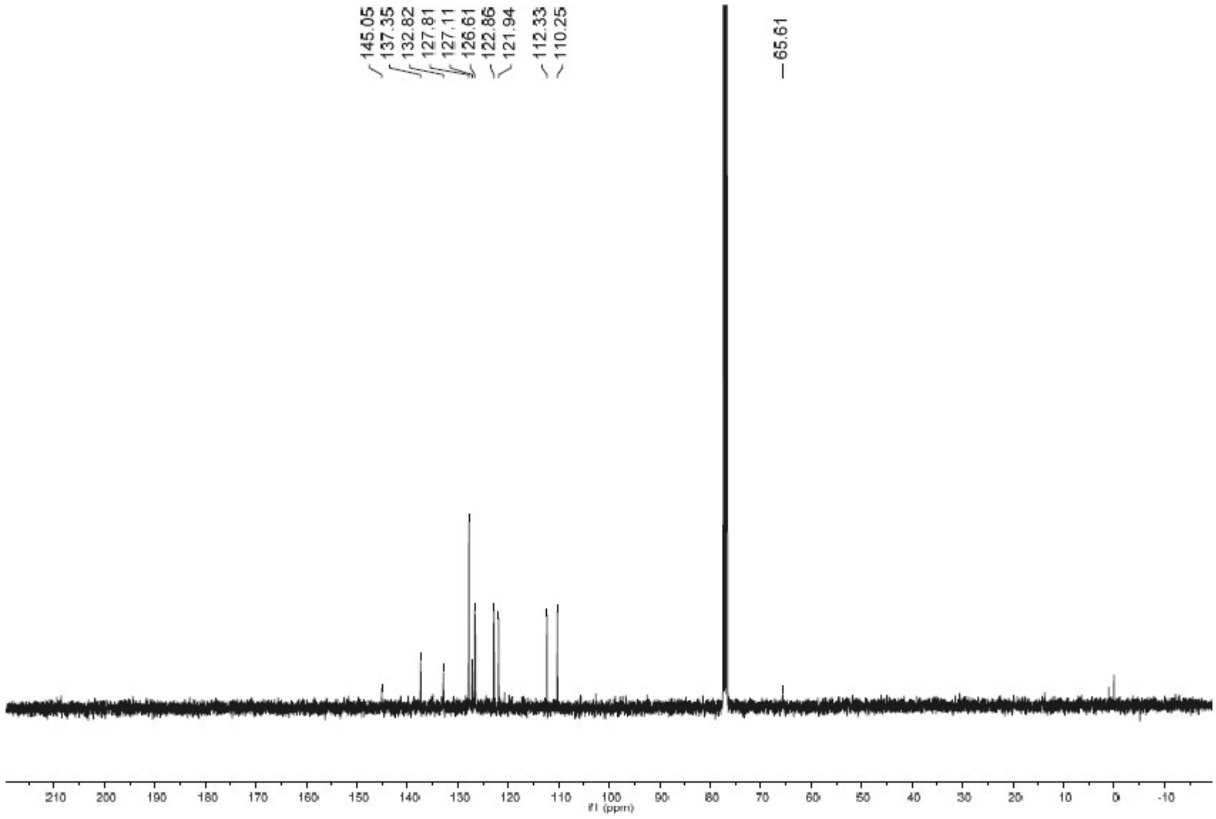

Figure 16. ^{13}C NMR **3h**

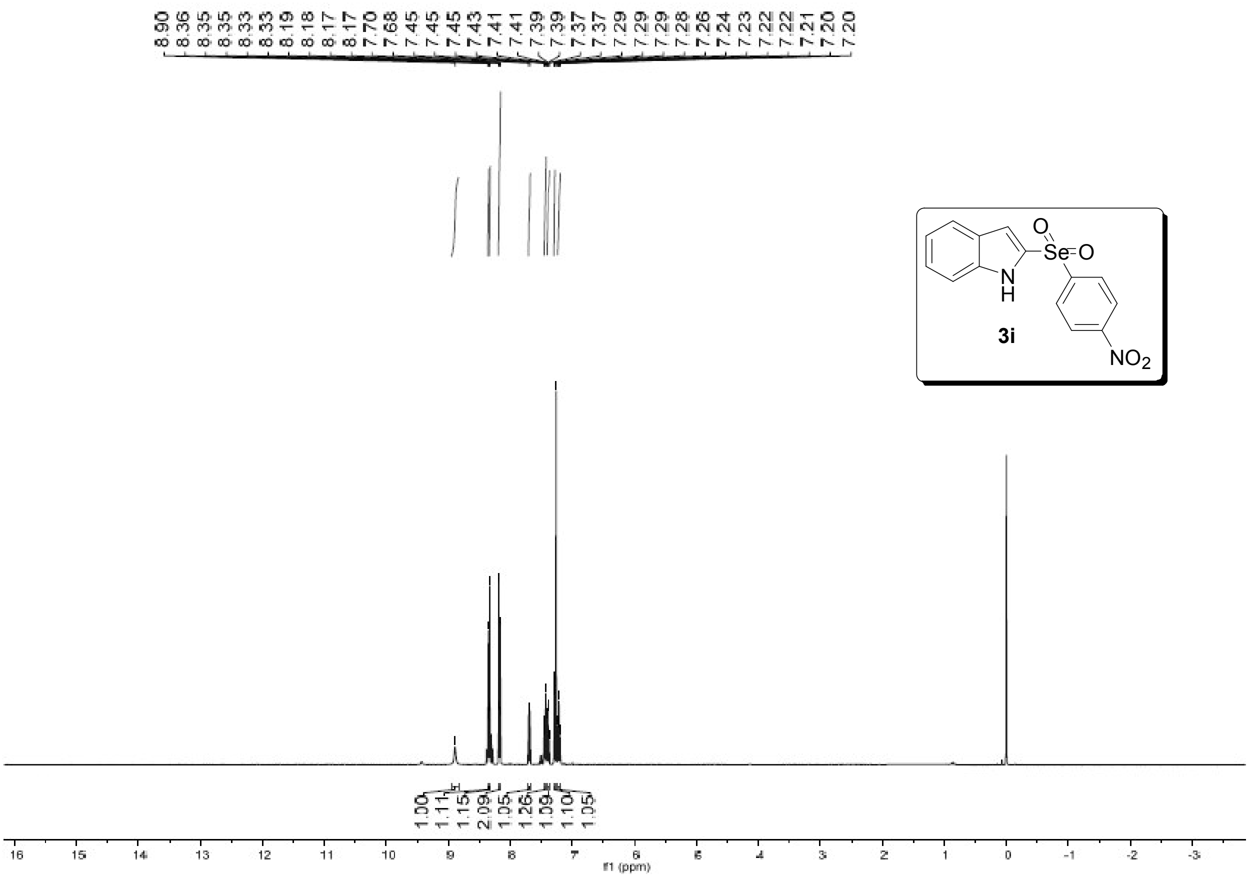

Figure 17. ^{1}H NMR **3i**

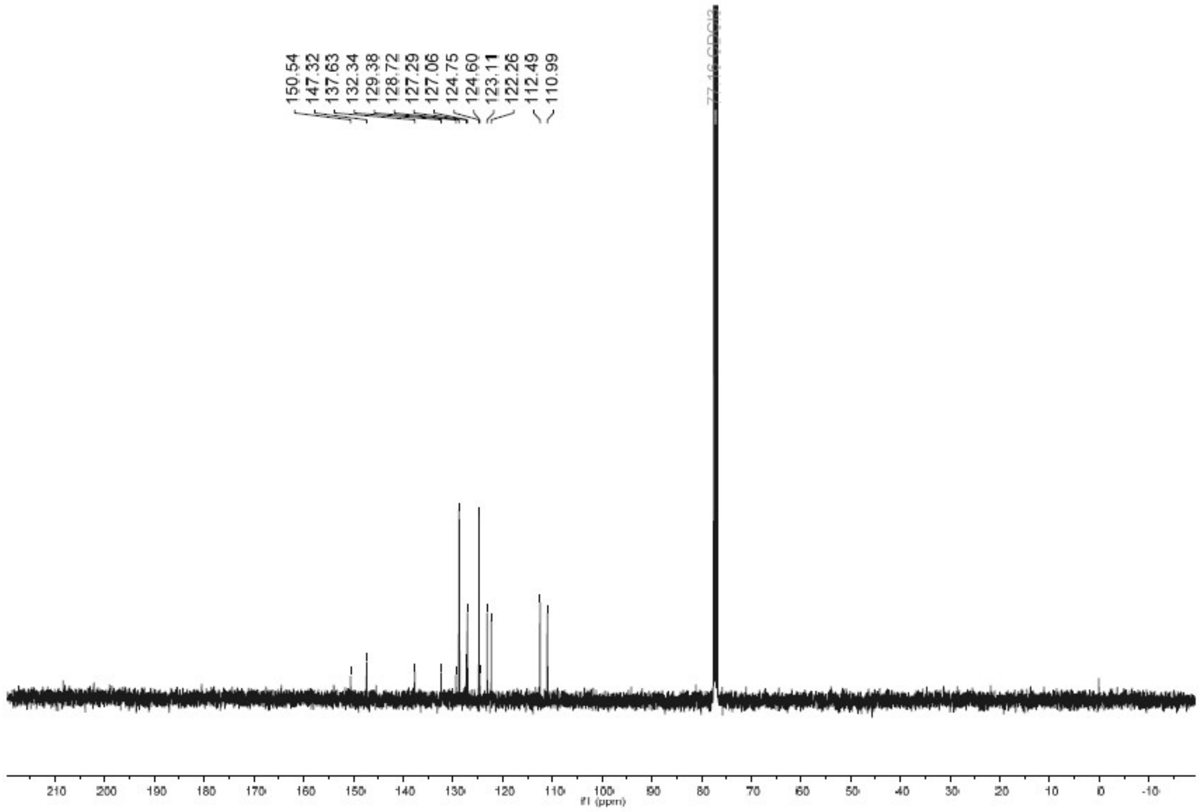

Figure 18. ^{13}C NMR **3i**

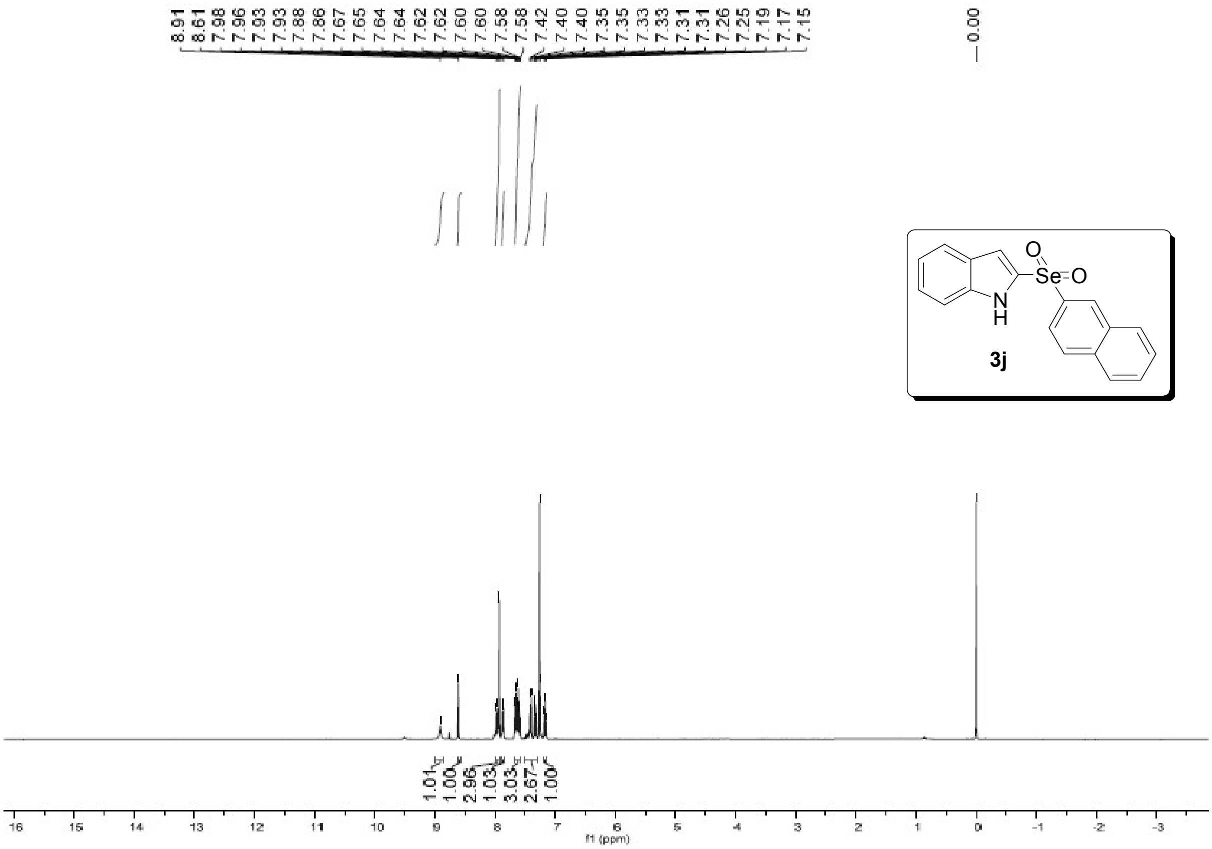

Figure 19. ^{1}H NMR **3j**

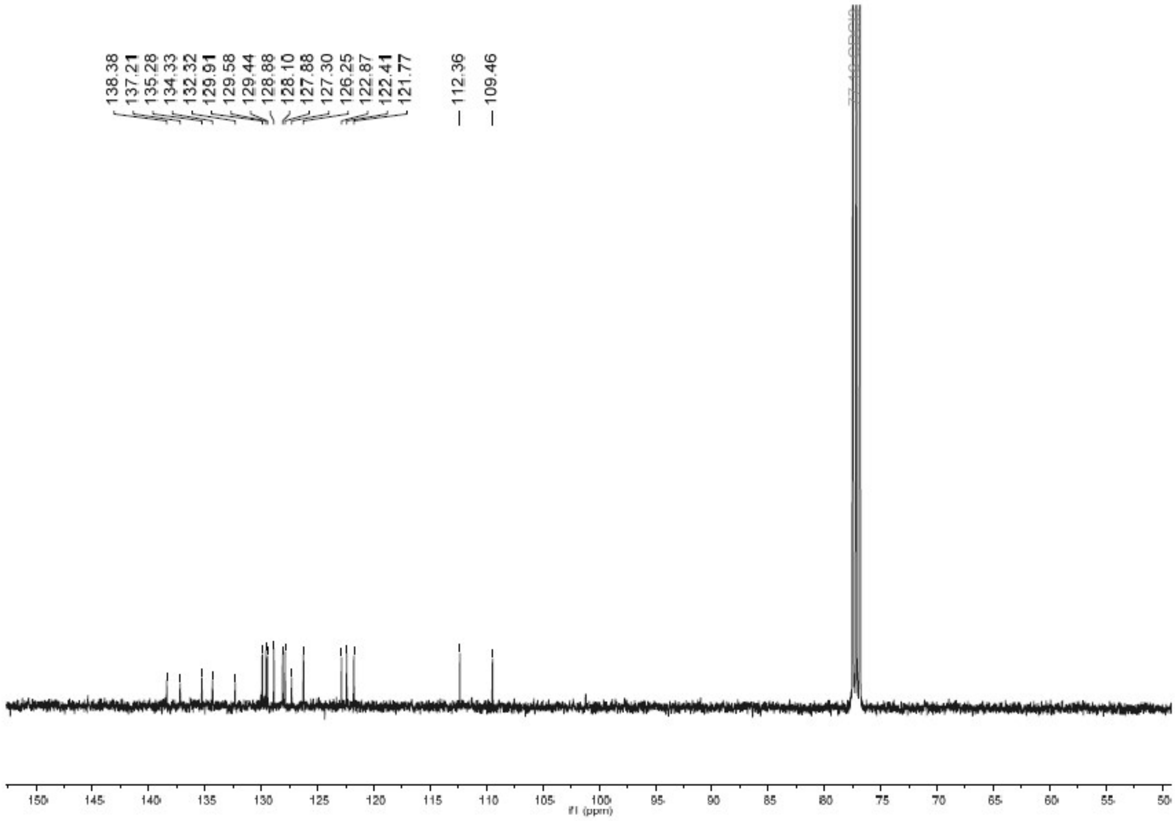

Figure 20. ^{13}C NMR **3j**

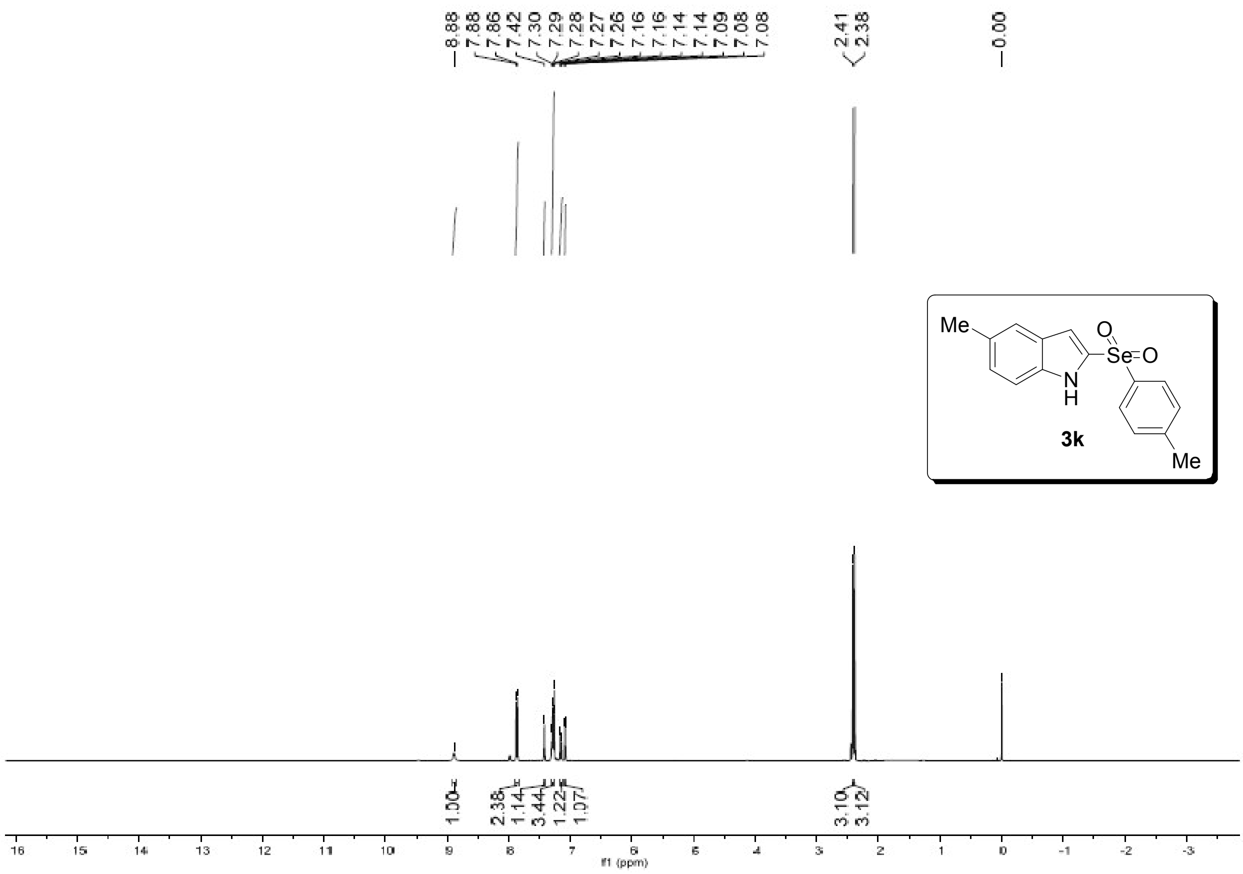

Figure 21. ^{1}H NMR **3k**

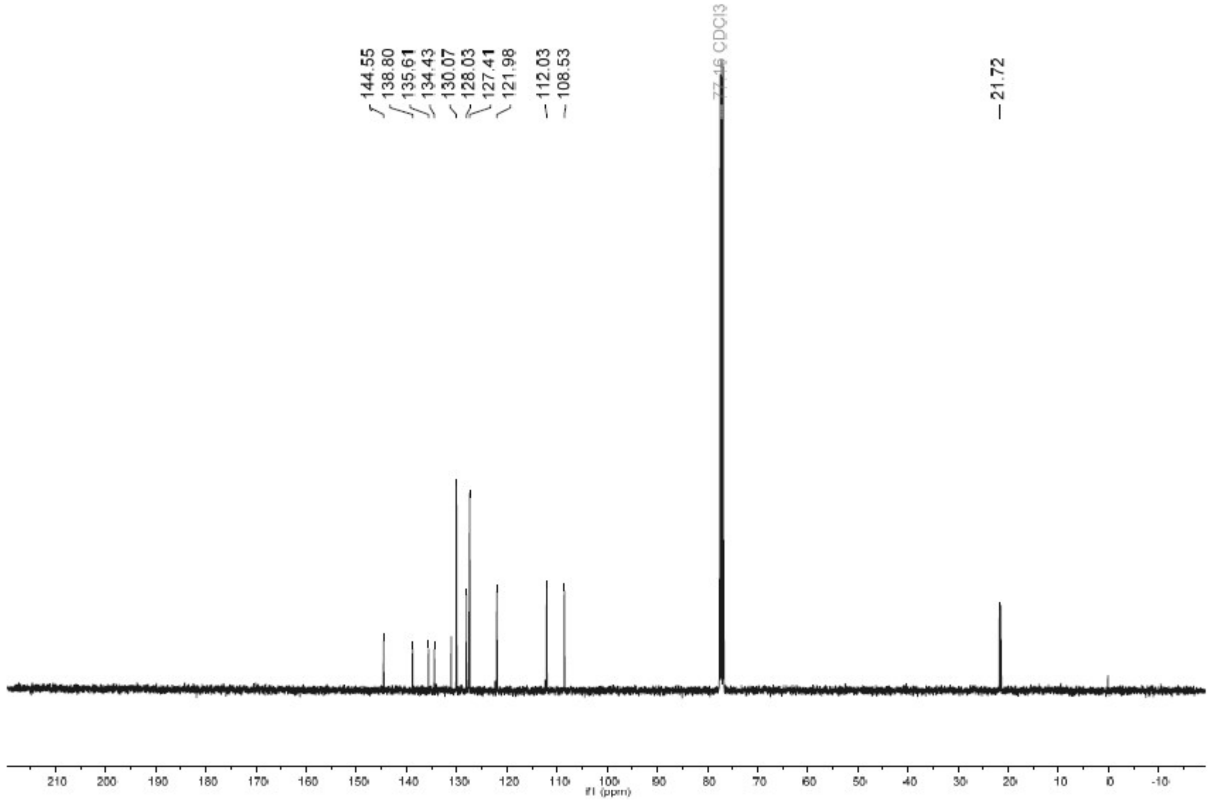

Figure 22. ^{13}C NMR **3k**

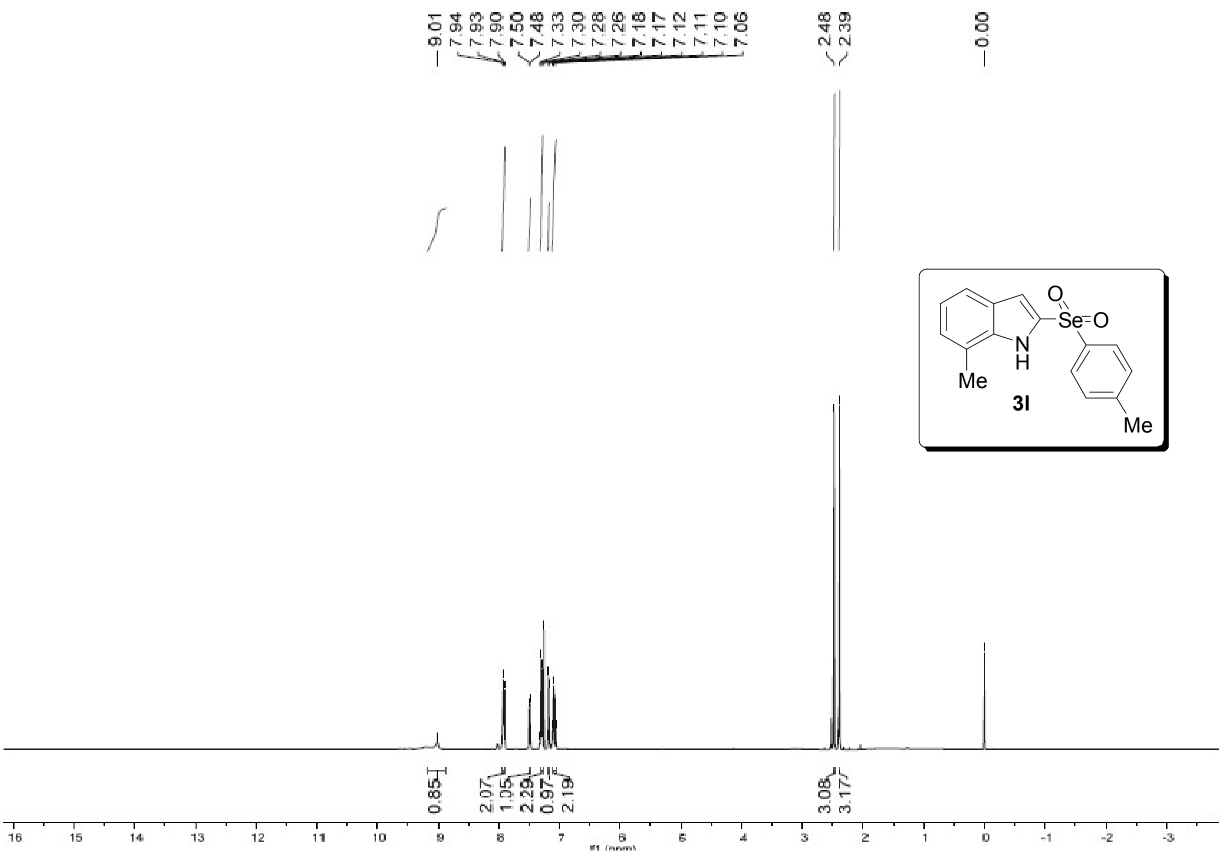

Figure 23. ^{1}H NMR **3l**

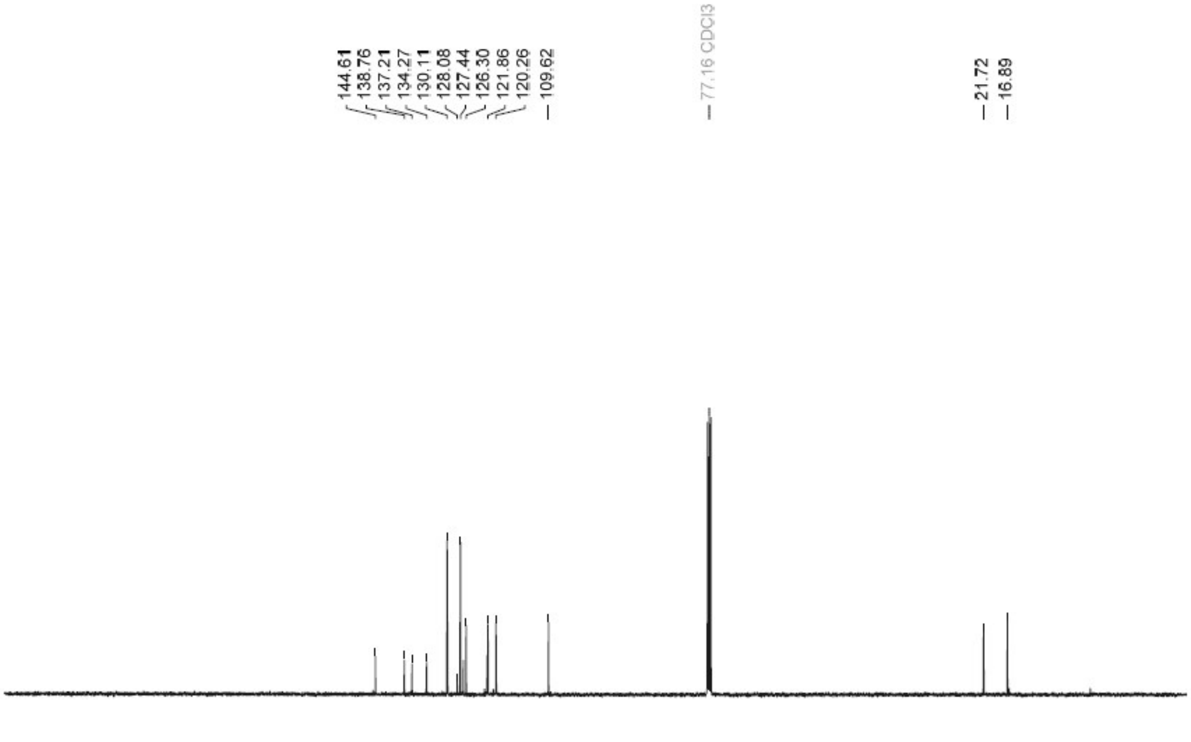

Figure 24. ^{13}C NMR **3l**

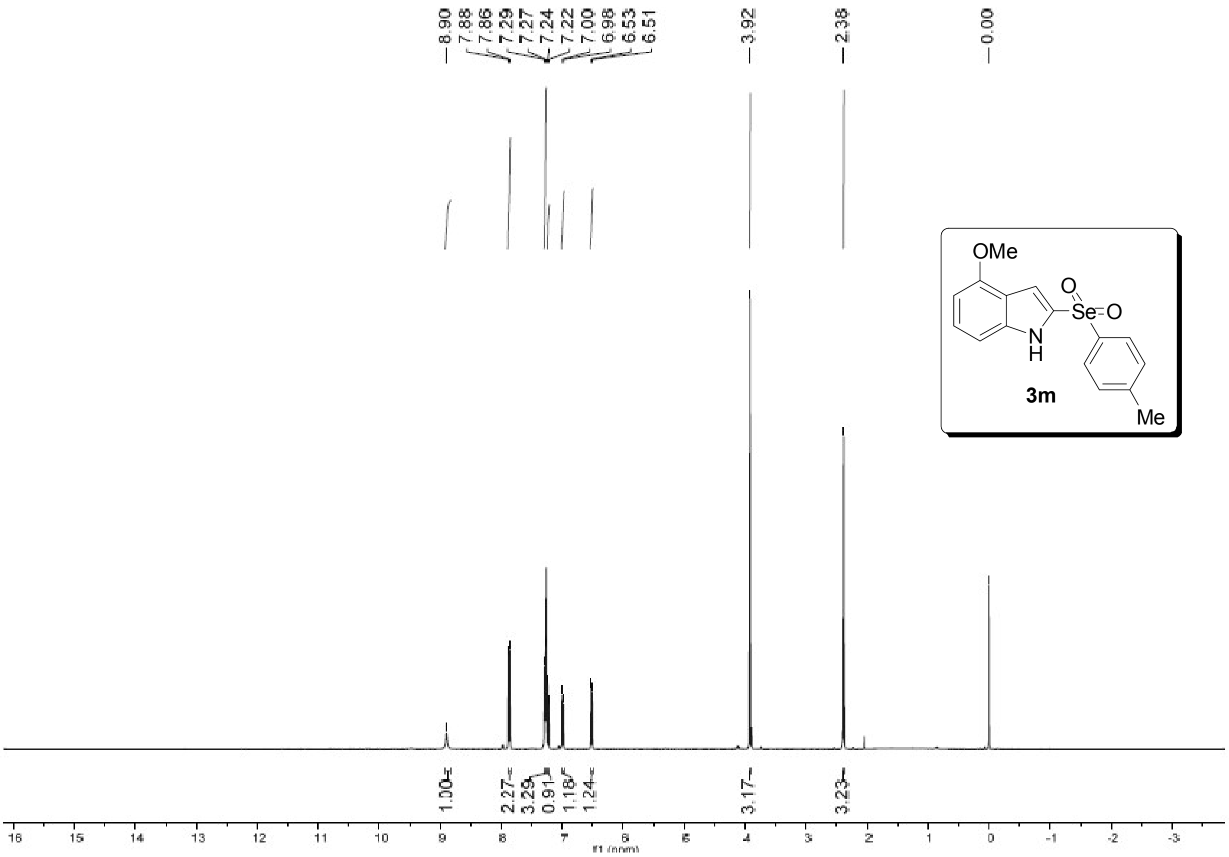

Figure 25. ^{1}H NMR **3m**

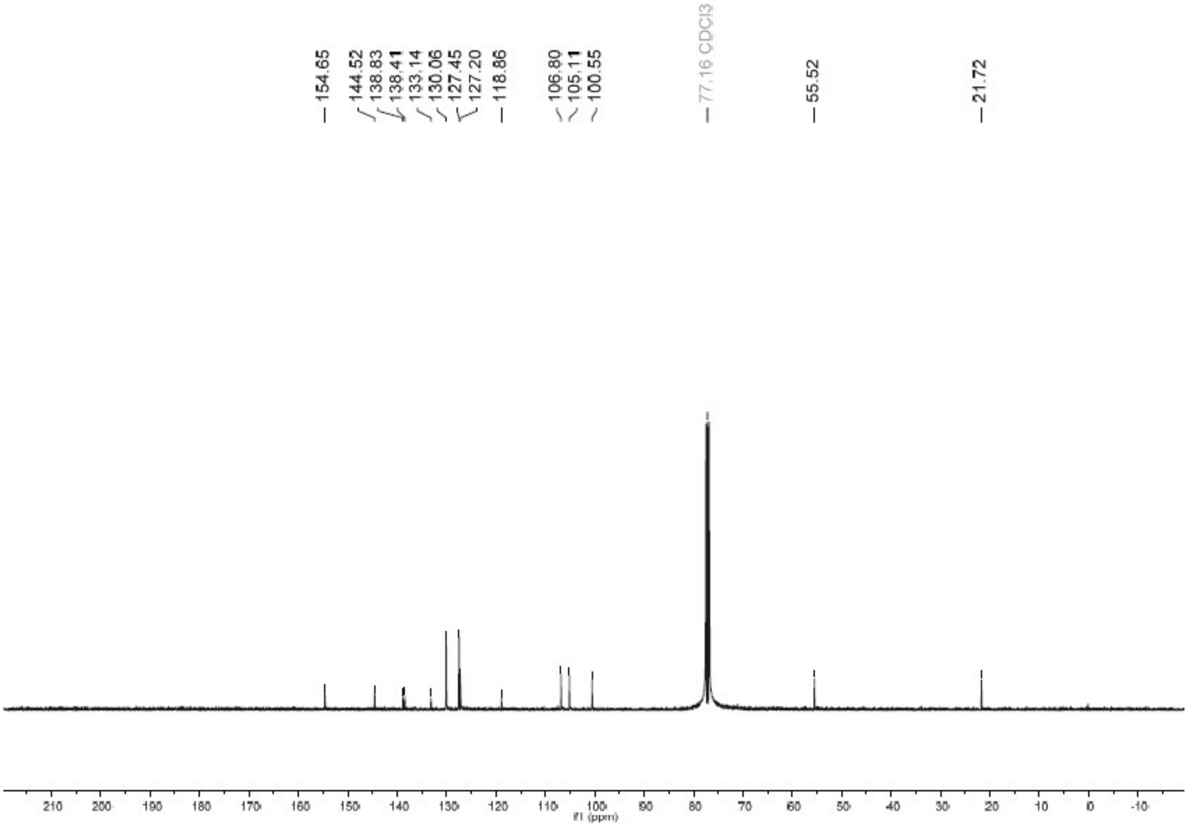

Figure 26. ^{13}C NMR **3m**

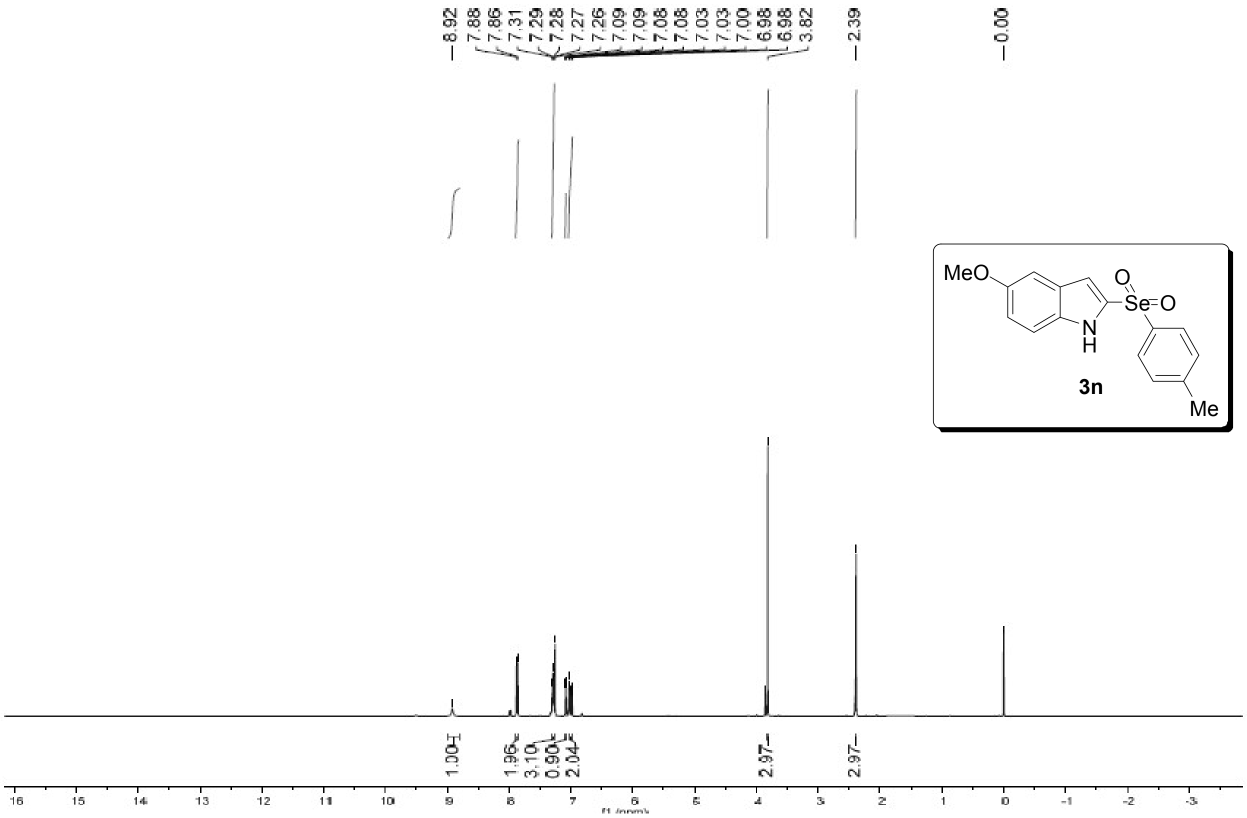

Figure 27. ¹H NMR **3n**

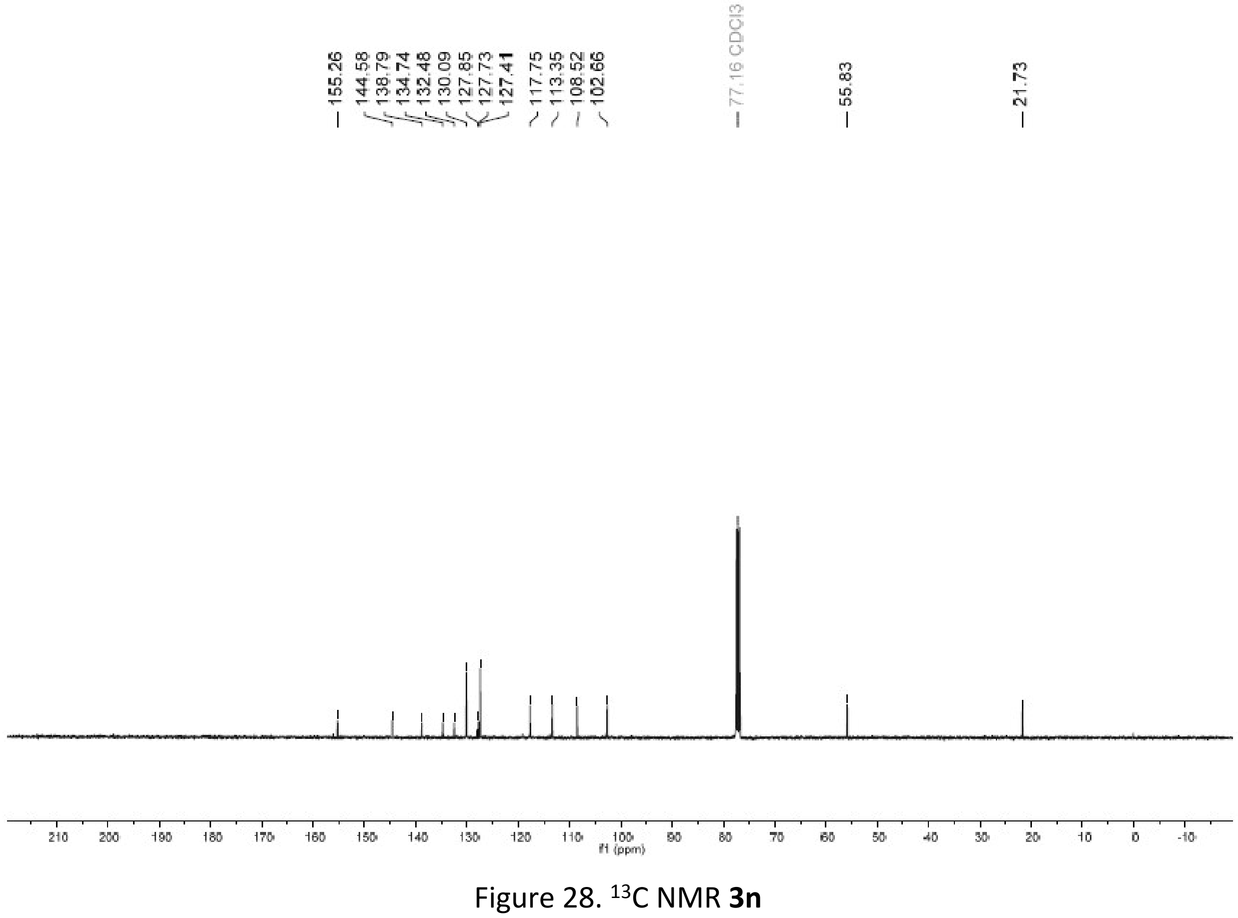

Figure 28. ¹³C NMR **3n**

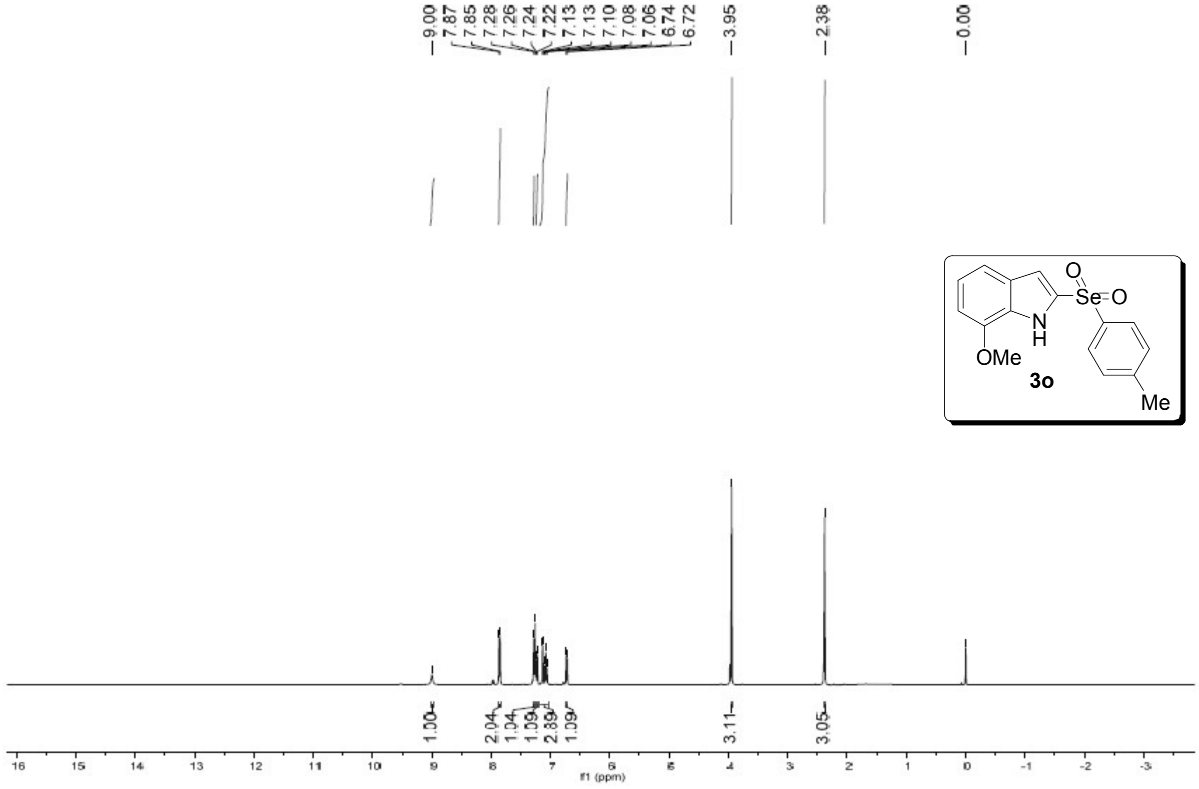

Figure 29. ^{1}H NMR **3o**

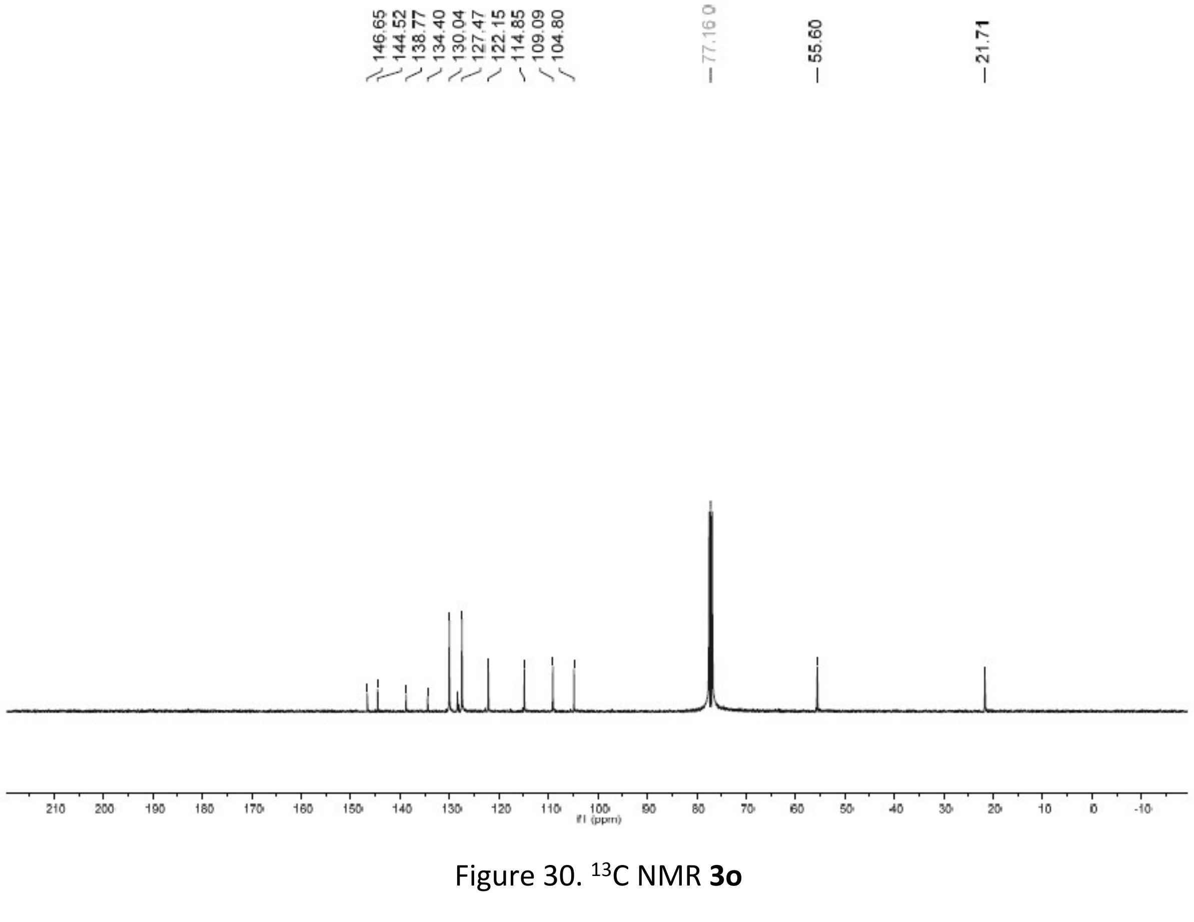

Figure 30. ^{13}C NMR **3o**

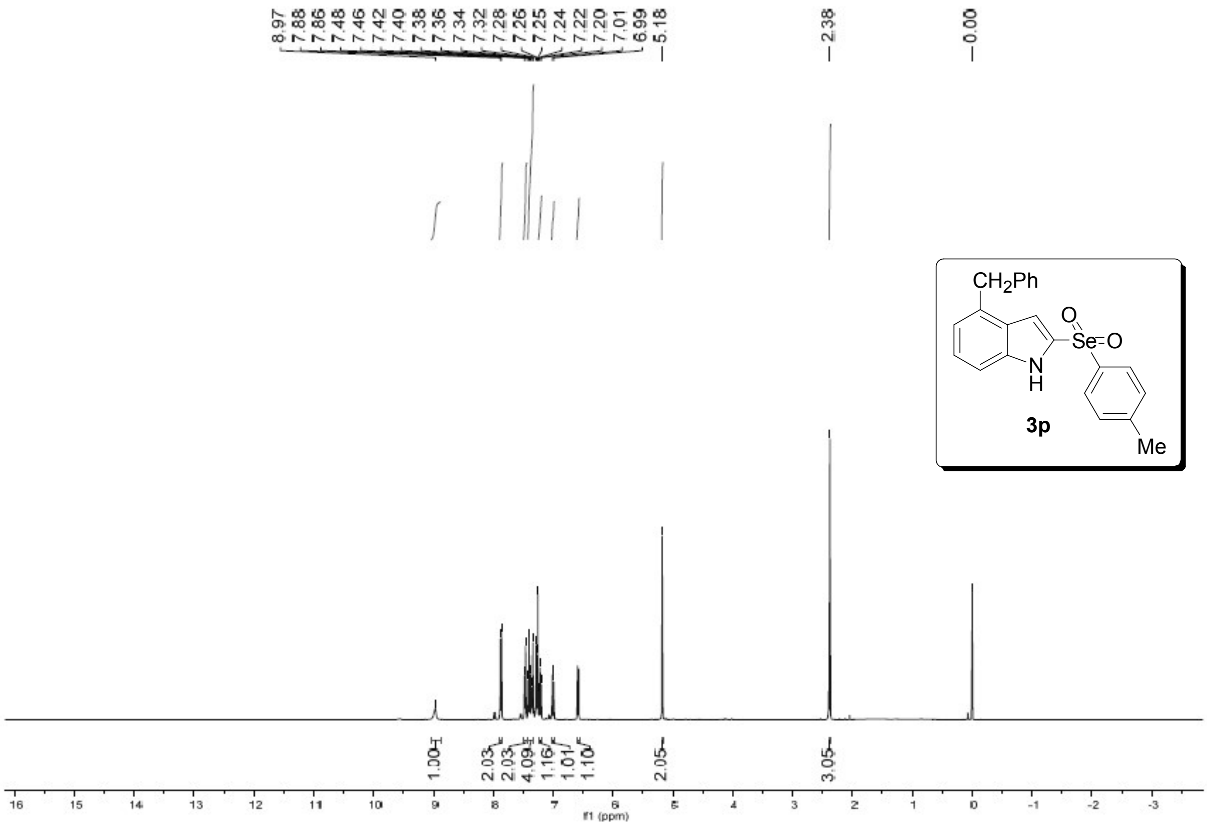

Figure 31. ^{1}H NMR **3p**

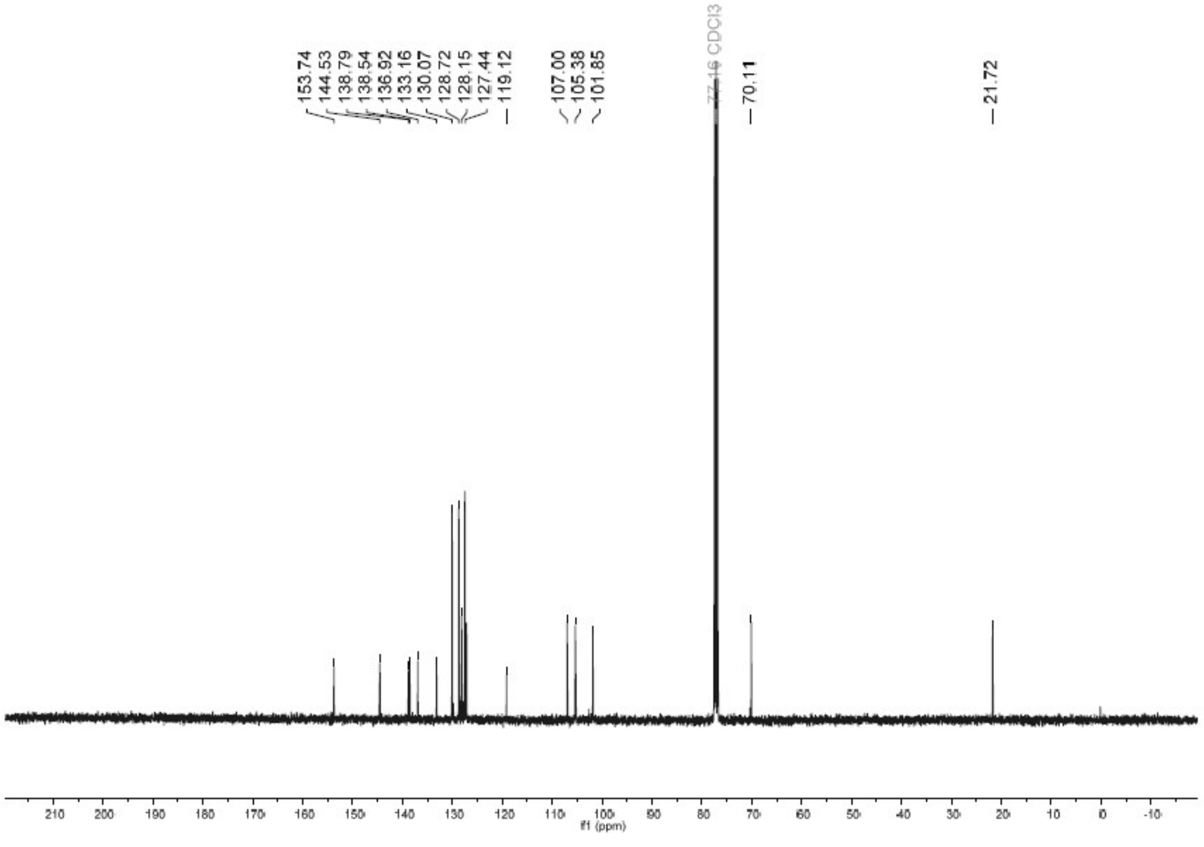

Figure 32. ^{13}C NMR **3p**

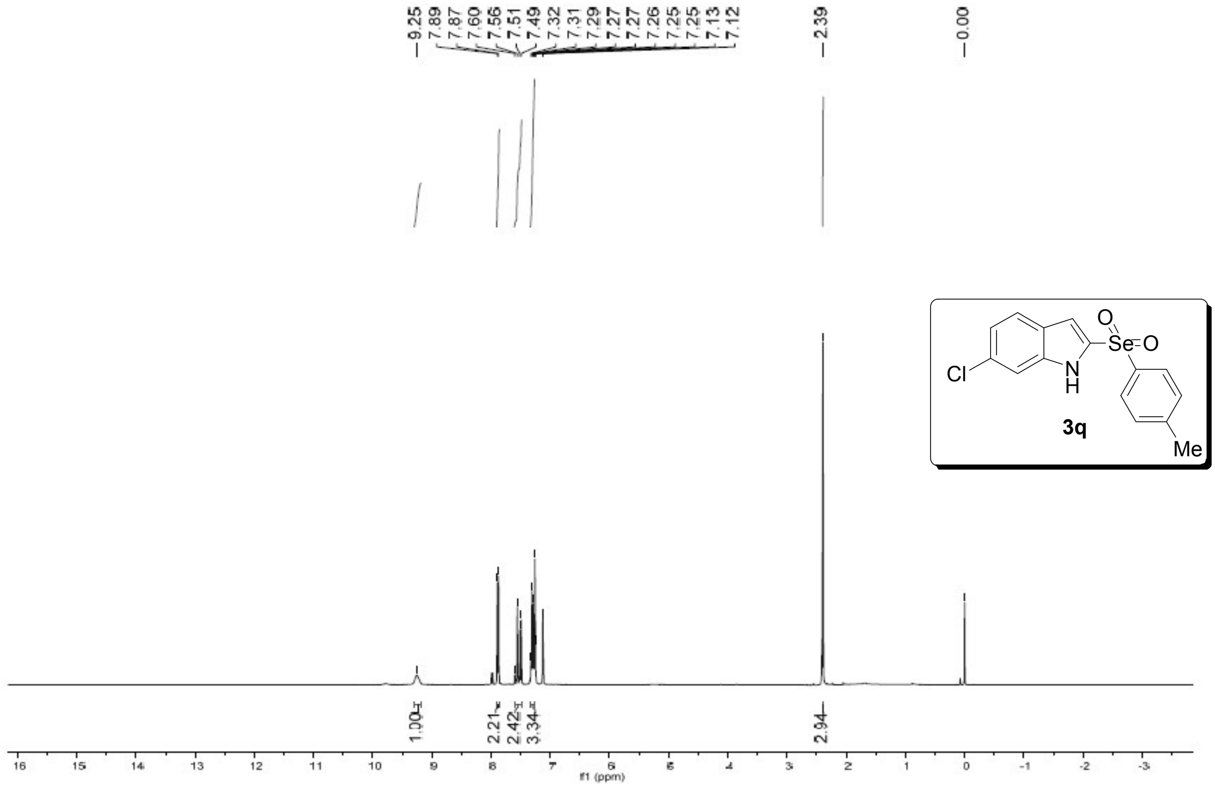

Figure 33. ^{1}H NMR **3q**

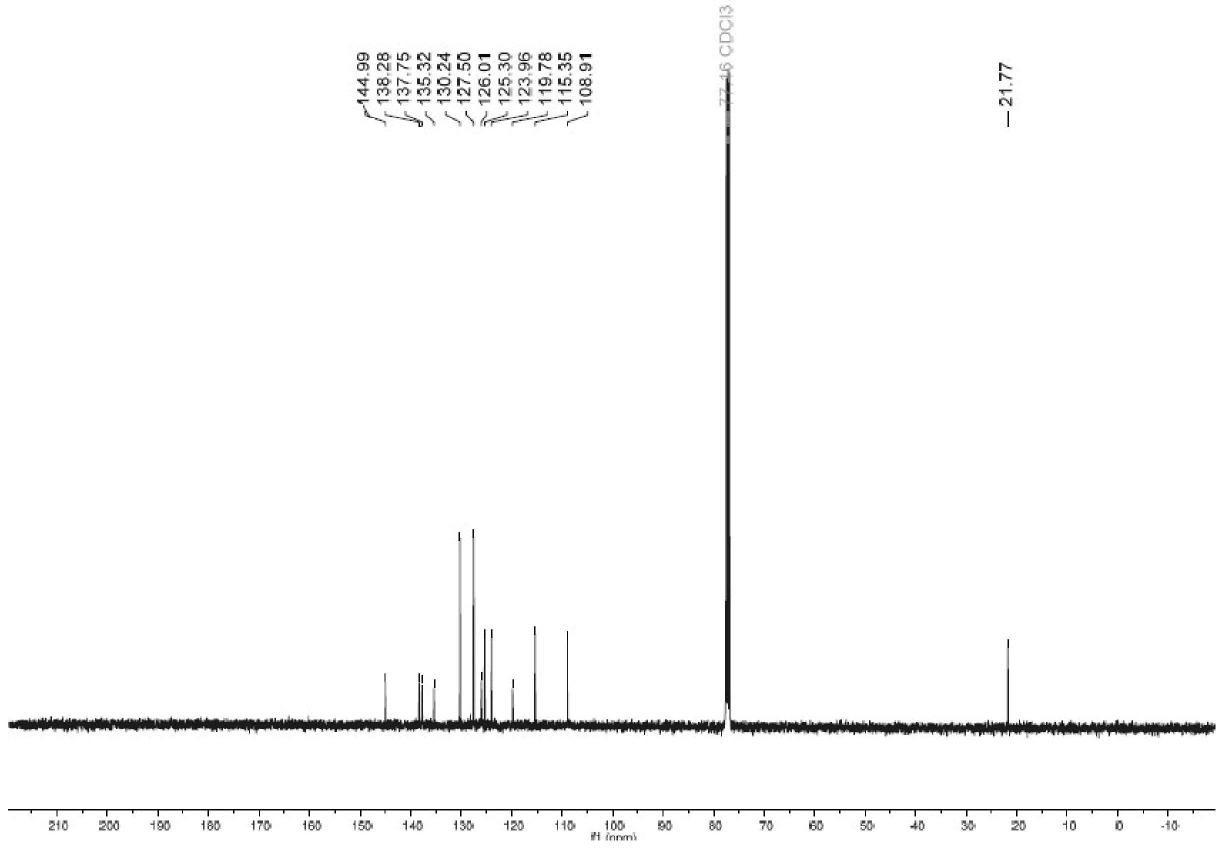

Figure 34. ^{13}C NMR **3q**

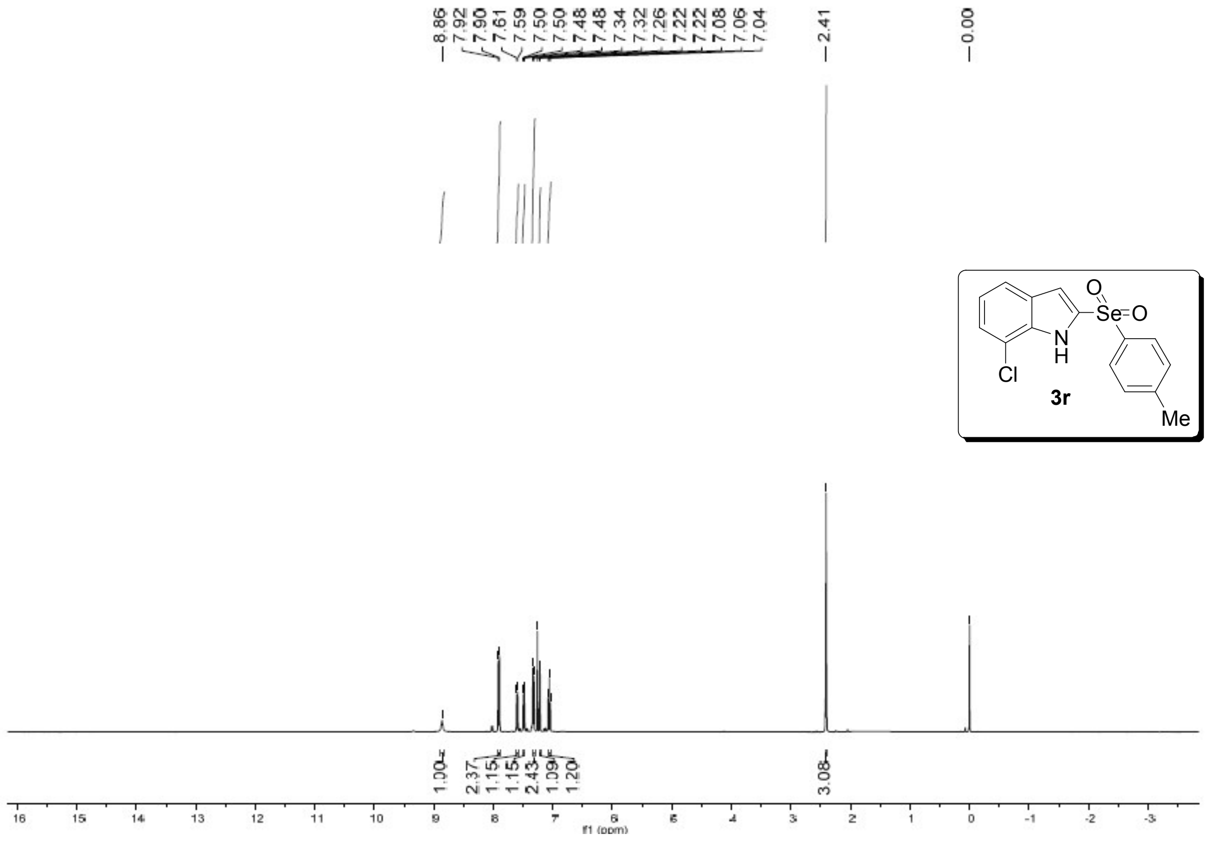

Figure 35. ^{1}H NMR **3r**

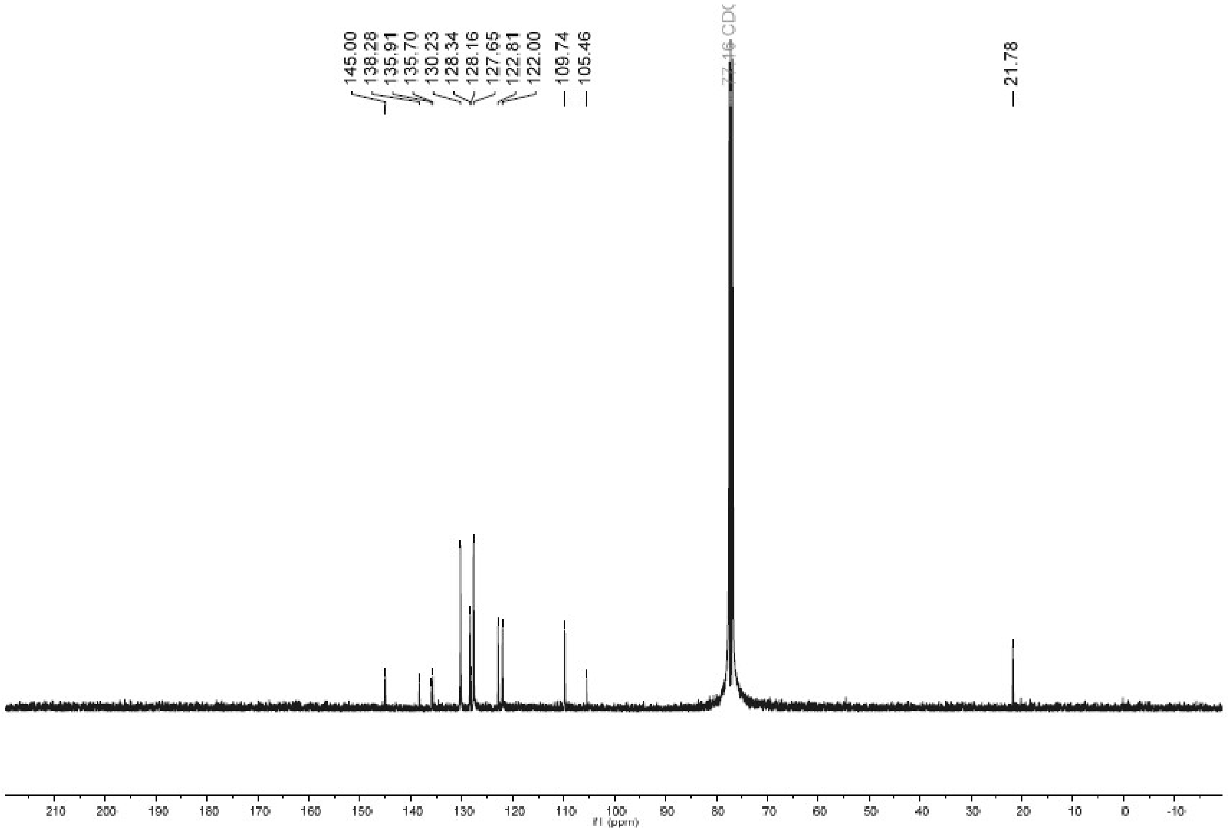

Figure 36. ^{13}C NMR **3r**

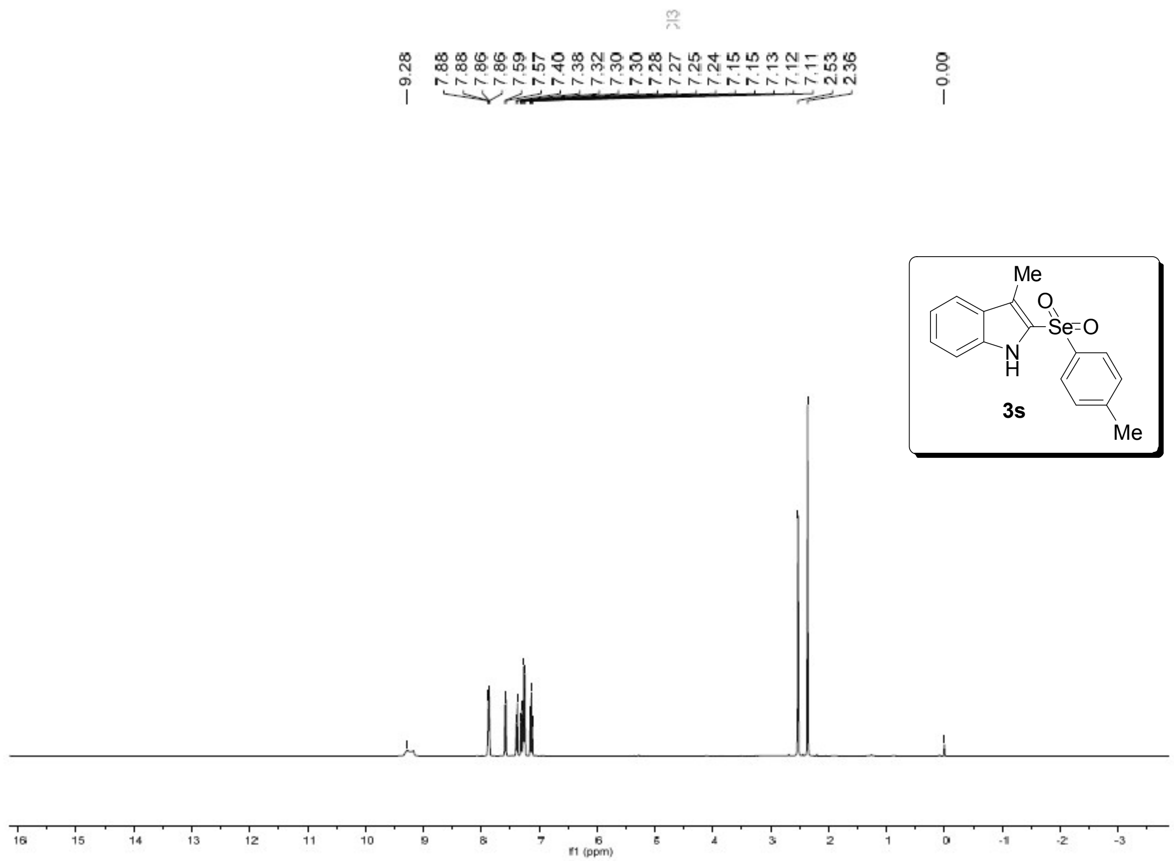

Figure 37. ^{1}H NMR **3s**

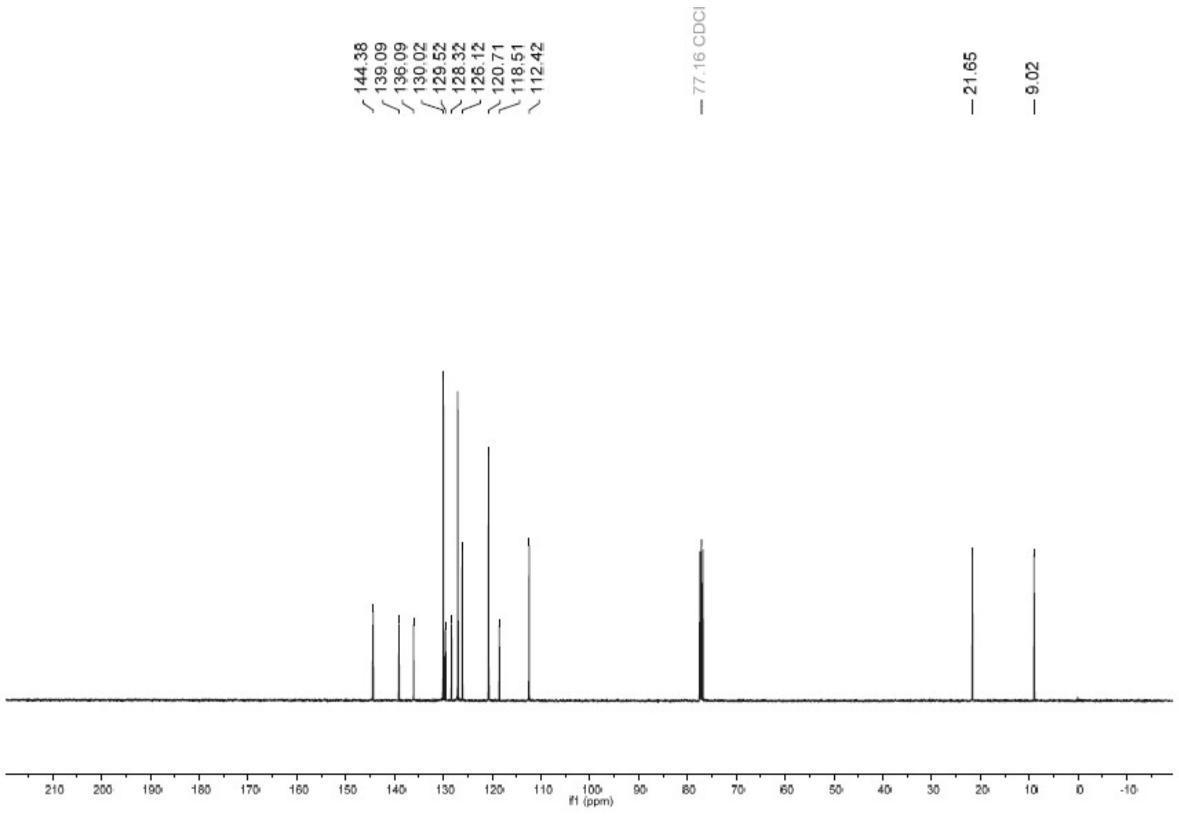

Figure 38. ^{13}C NMR **3s**

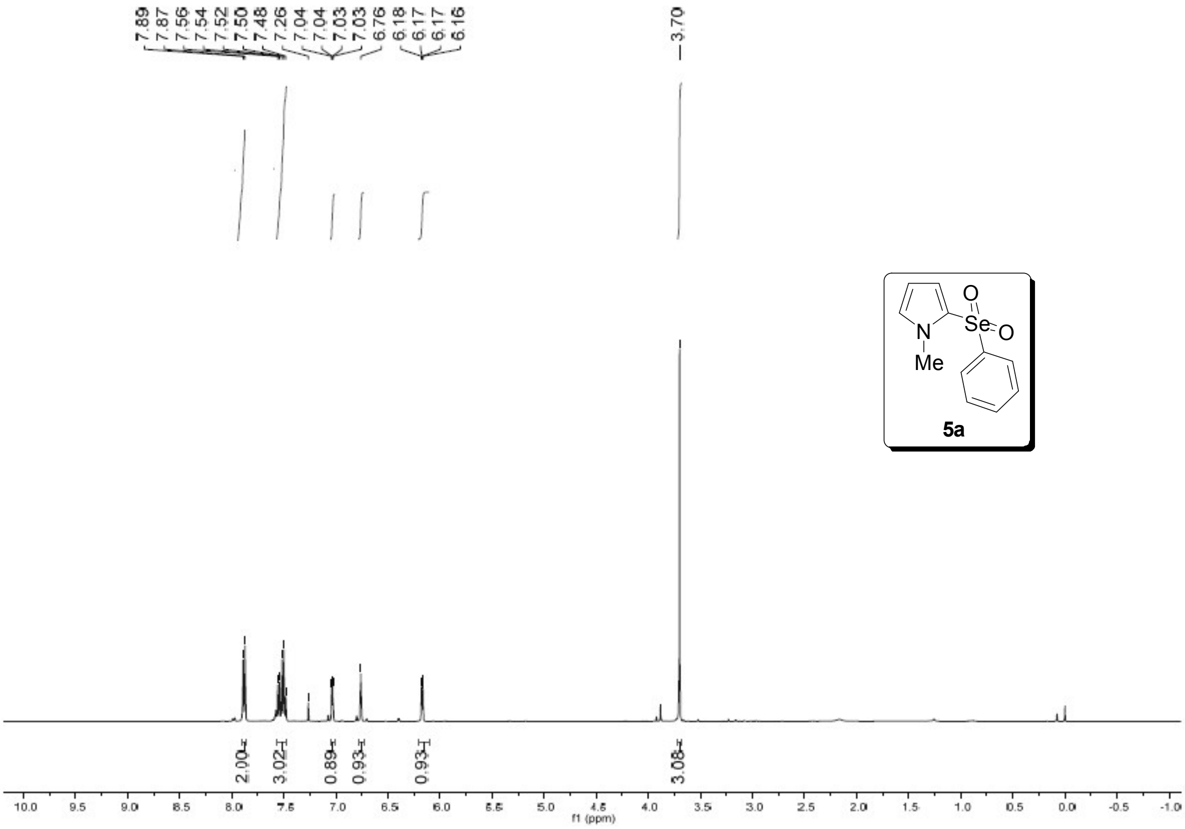

Figure 39. ^{1}H NMR **5a**

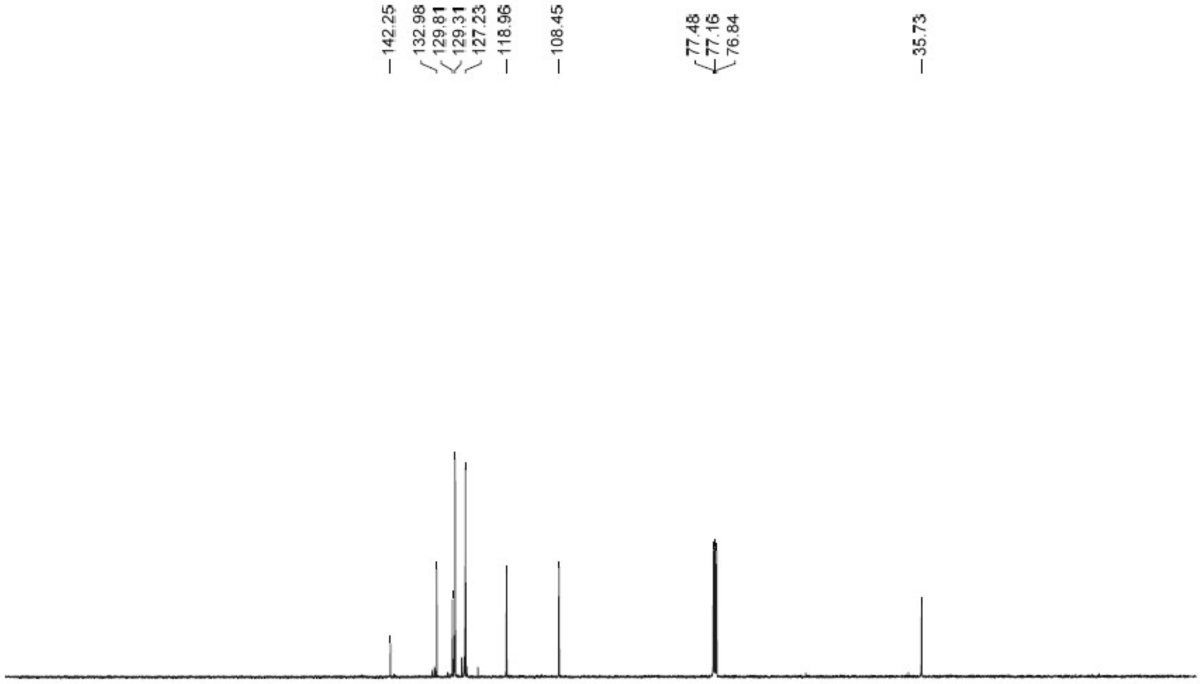

Figure 40. ^{13}C NMR **5a**

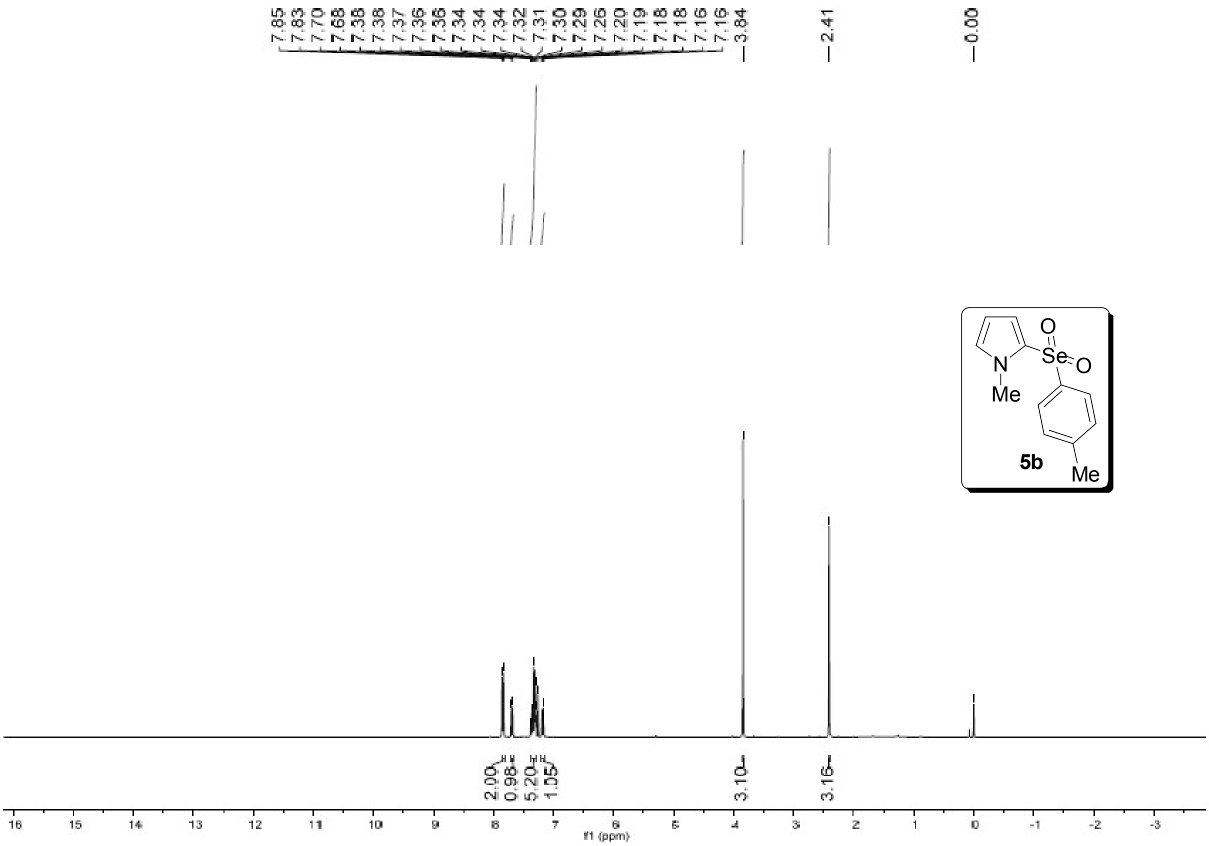

Figure 41. ^{1}H NMR **5b**

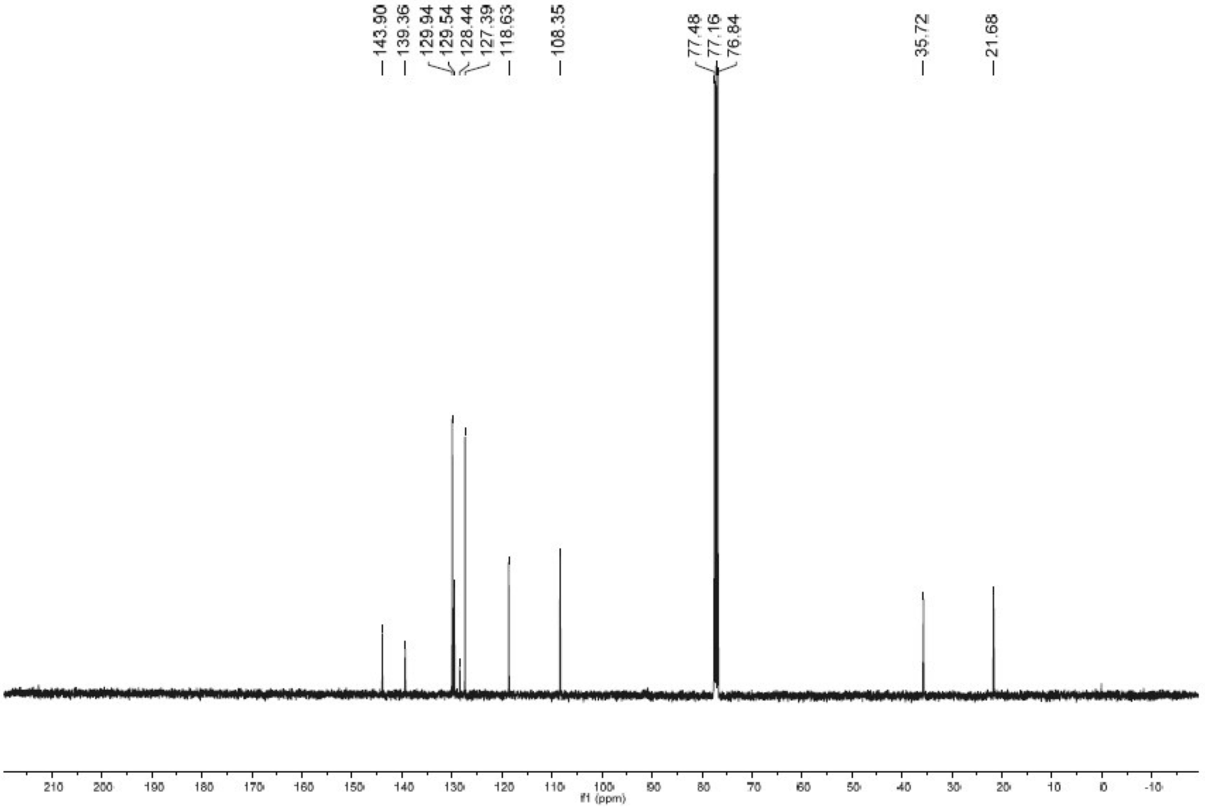

Figure 42. ^{13}C NMR **5b**

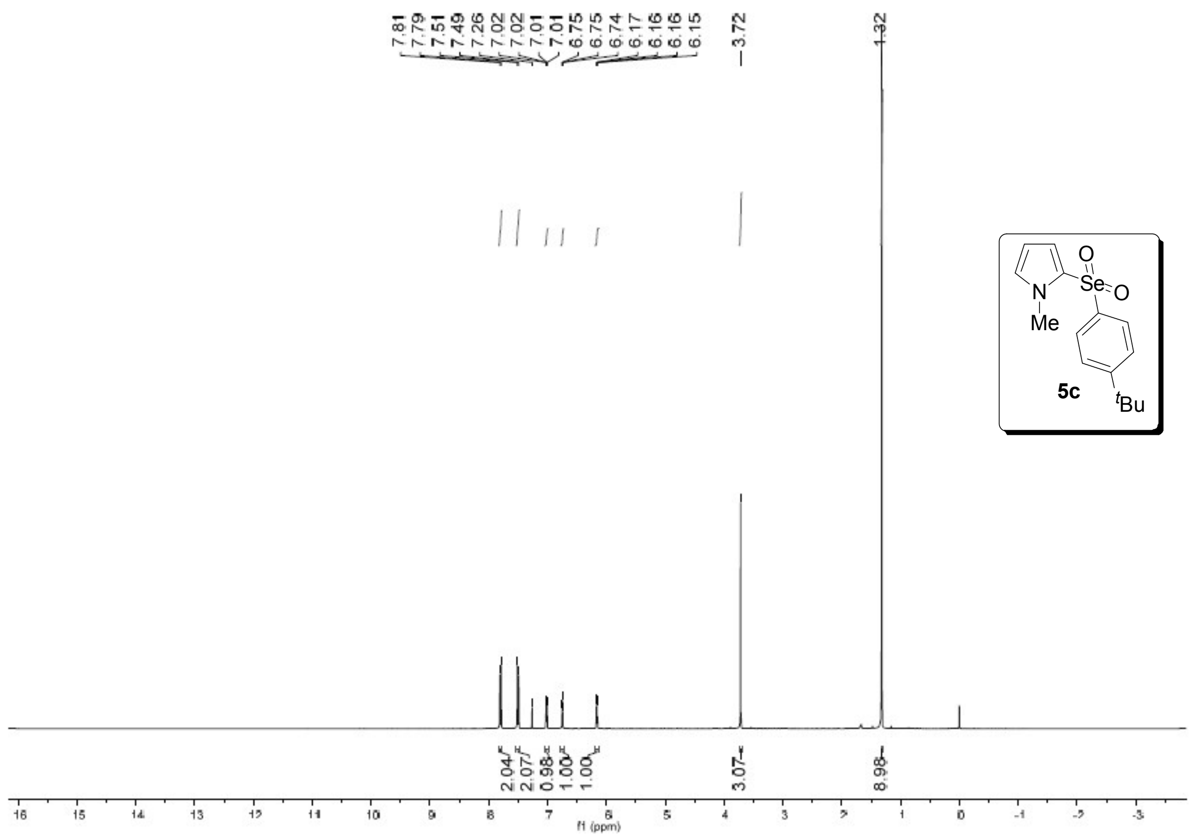

Figure 43. ^{1}H NMR **5c**

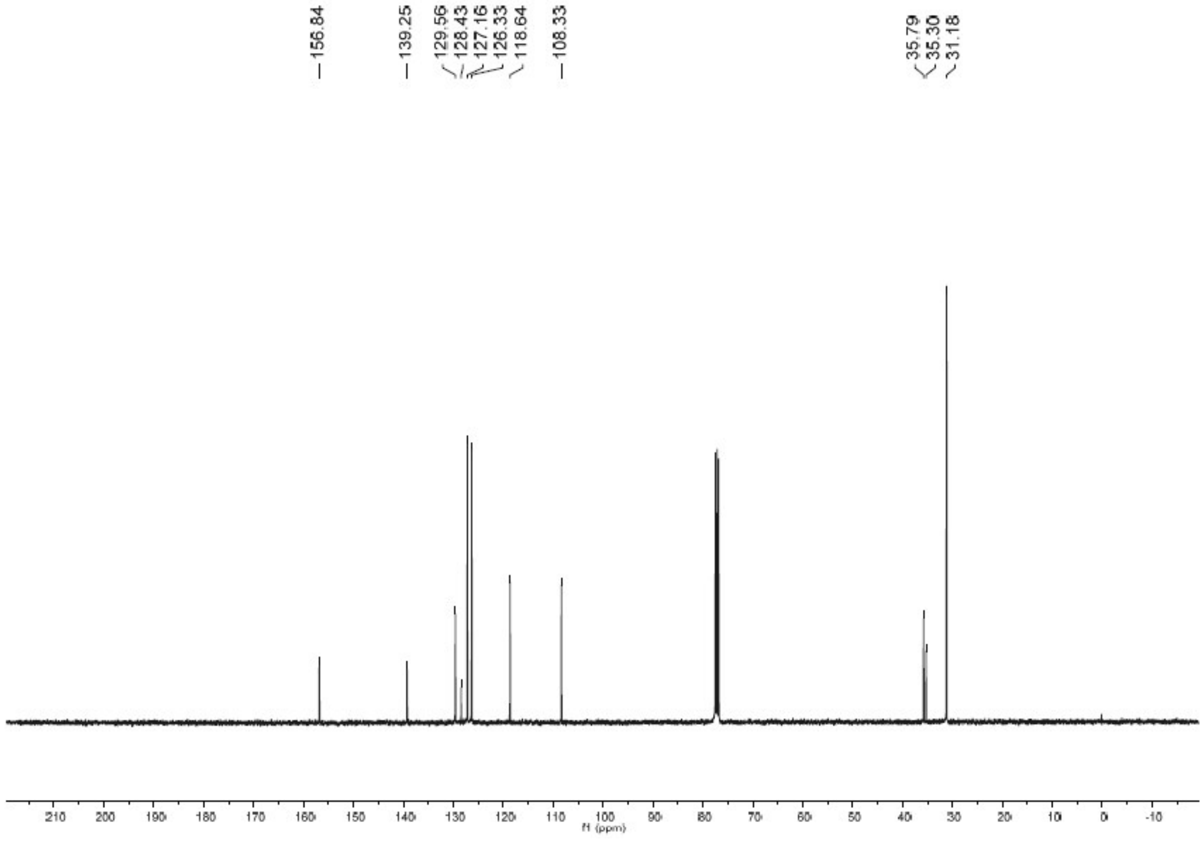

Figure 44. ^{13}C NMR **5c**

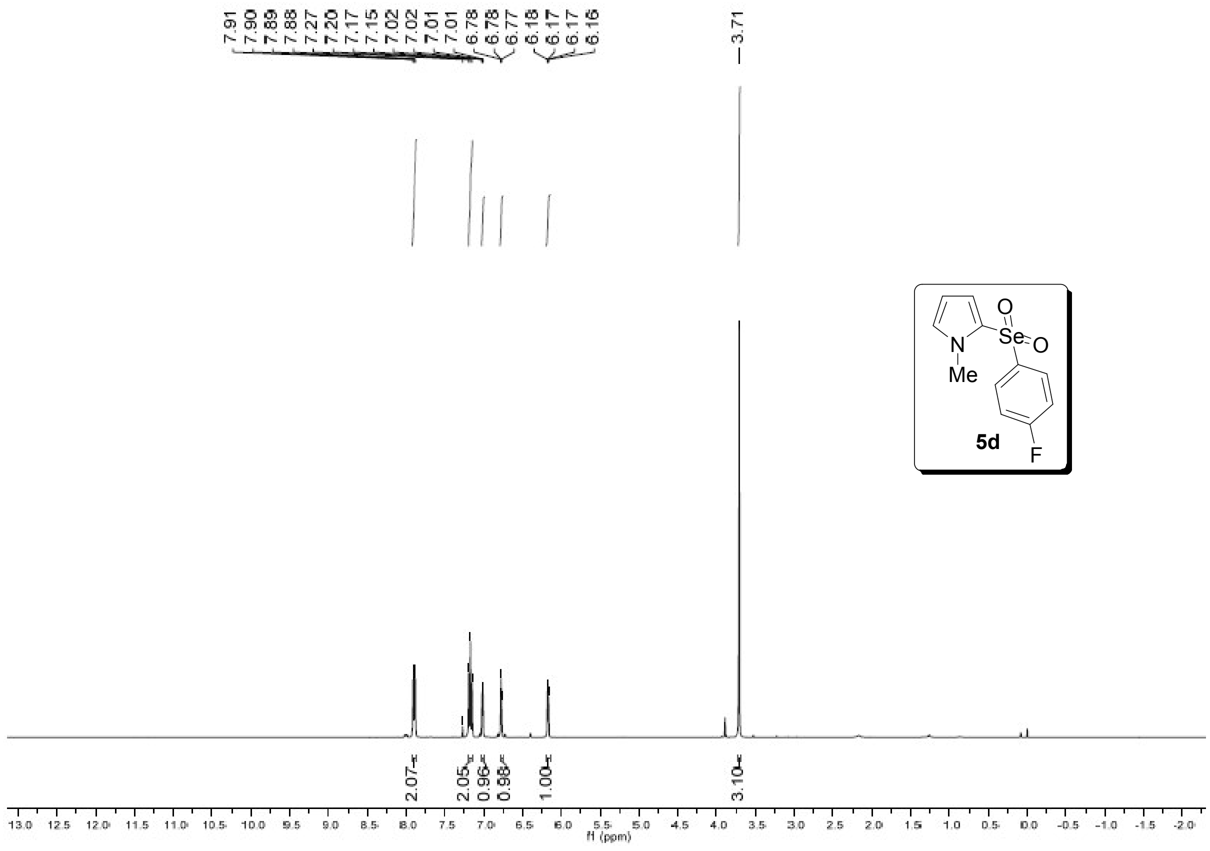

Figure 45. ^{1}H NMR **5d**

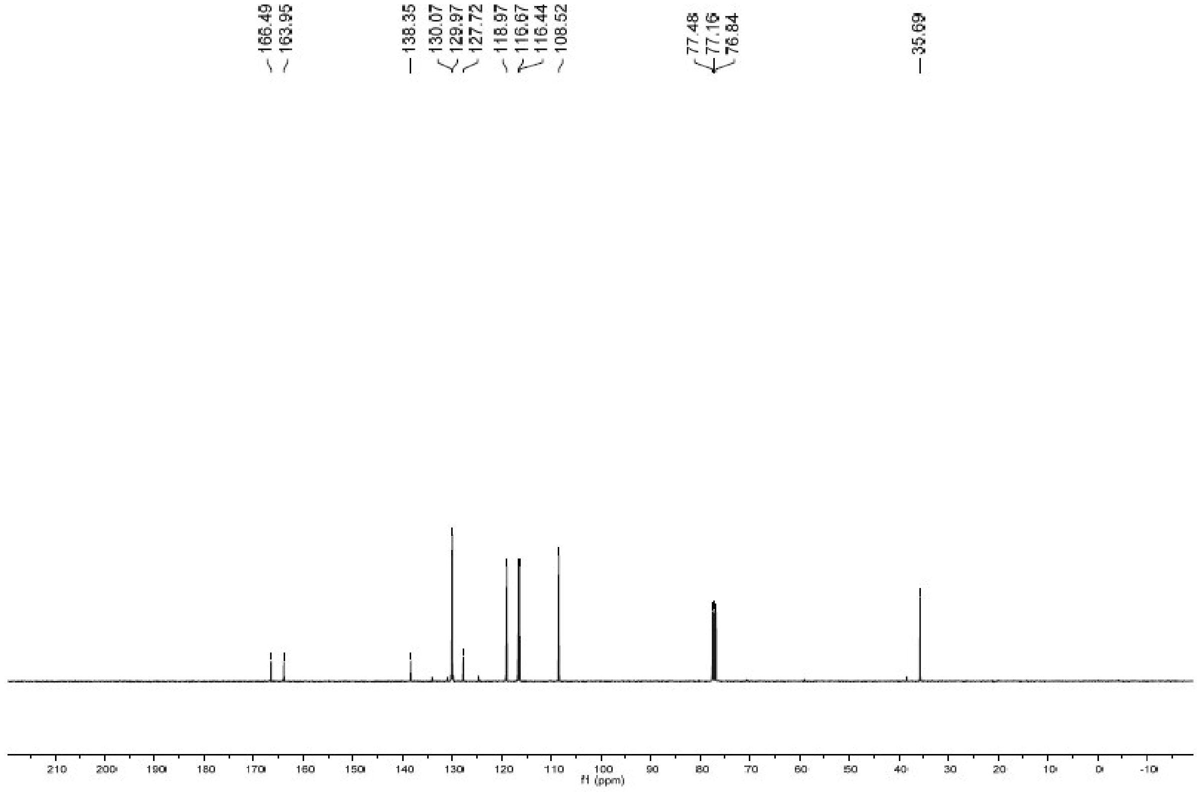

Figure 46. ^{13}C NMR **5d**

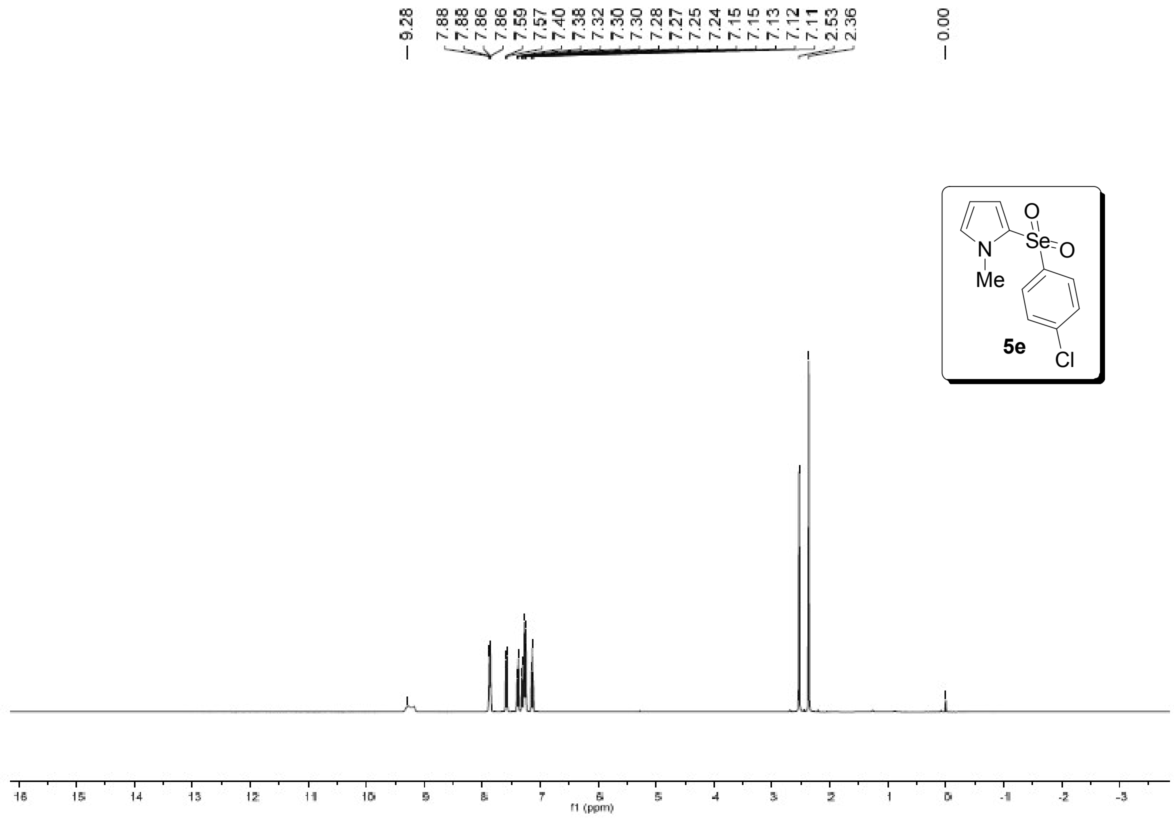

Figure 47. ^{1}H NMR **5e**

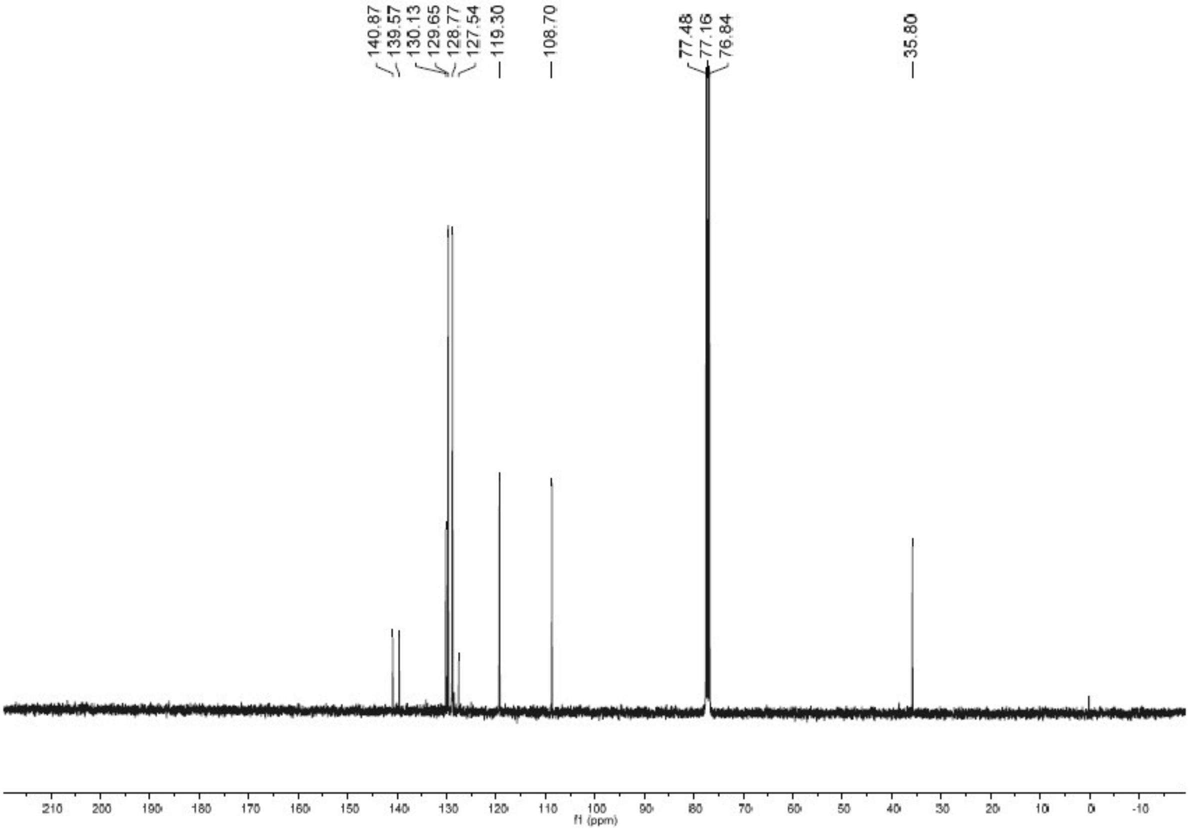

Figure 48. ^{13}C NMR **5e**

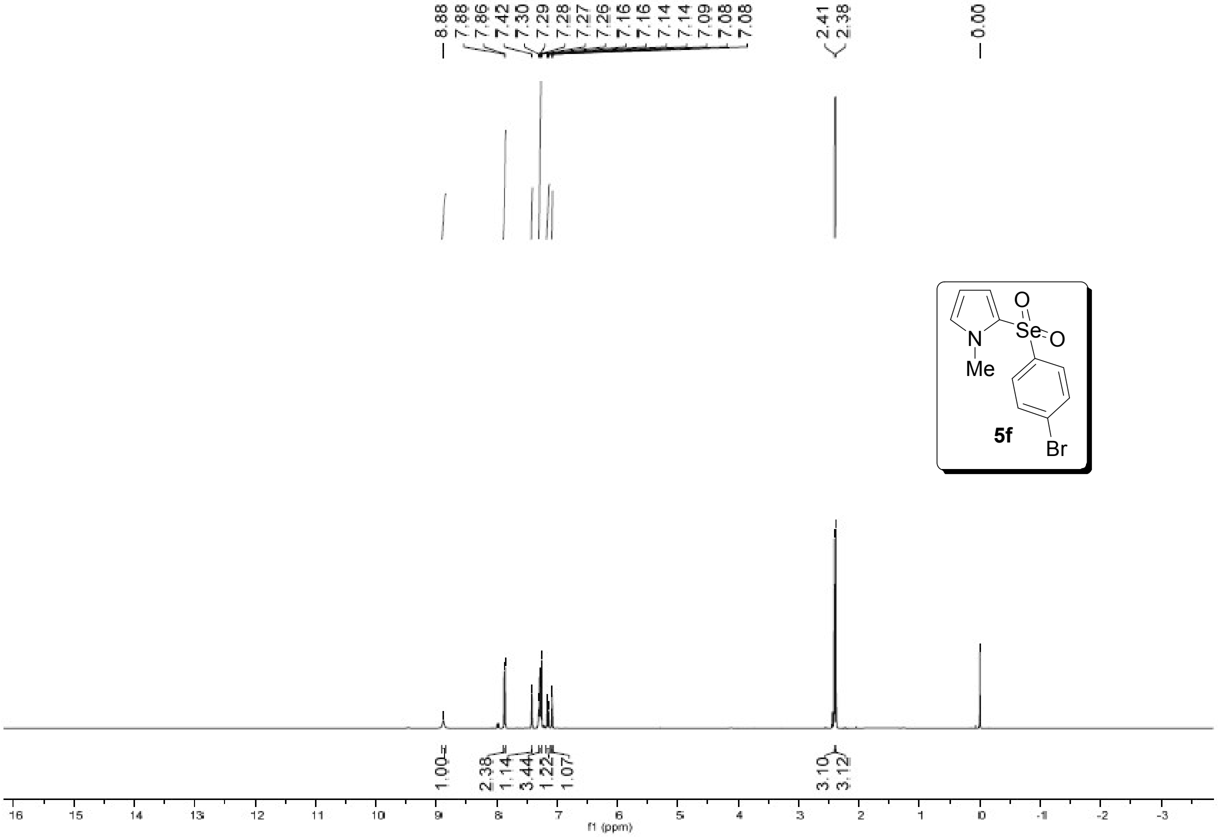

Figure 49. ^{1}H NMR **5f**

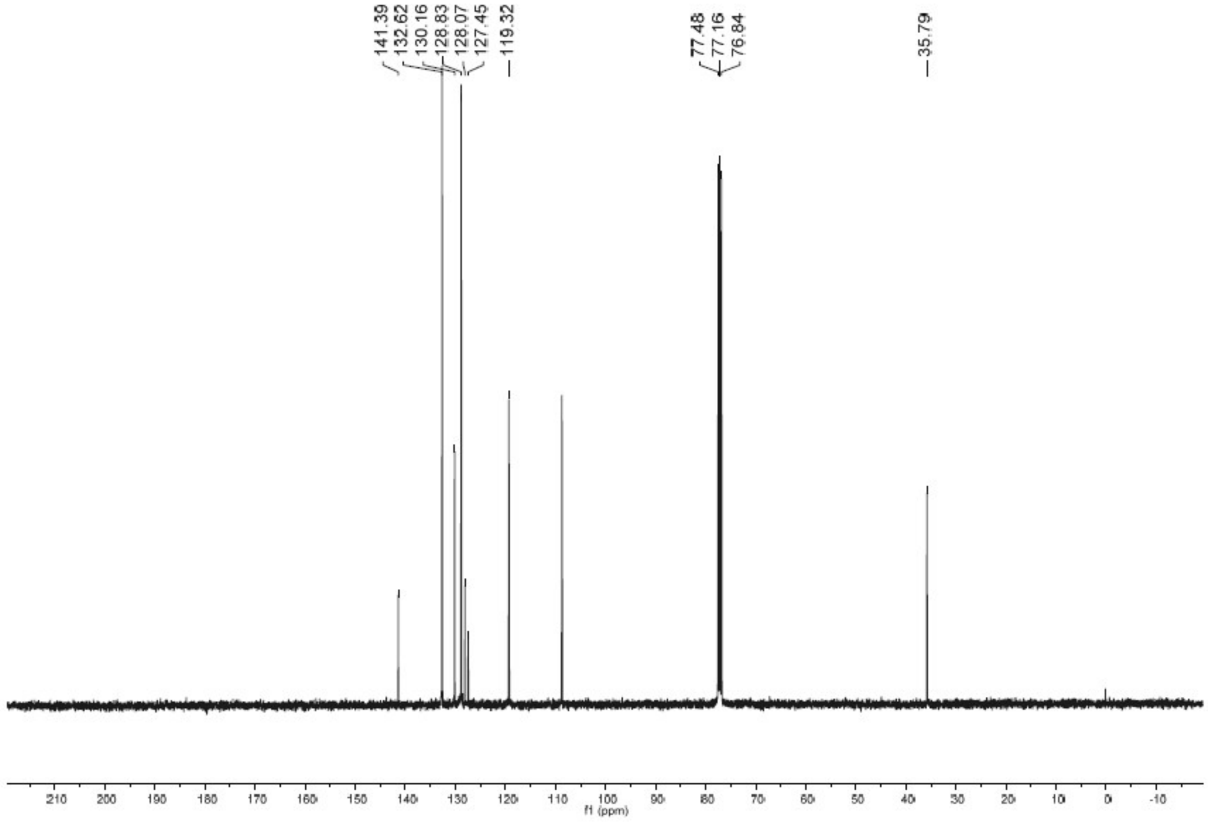

Figure 50. ^{13}C NMR **5f**

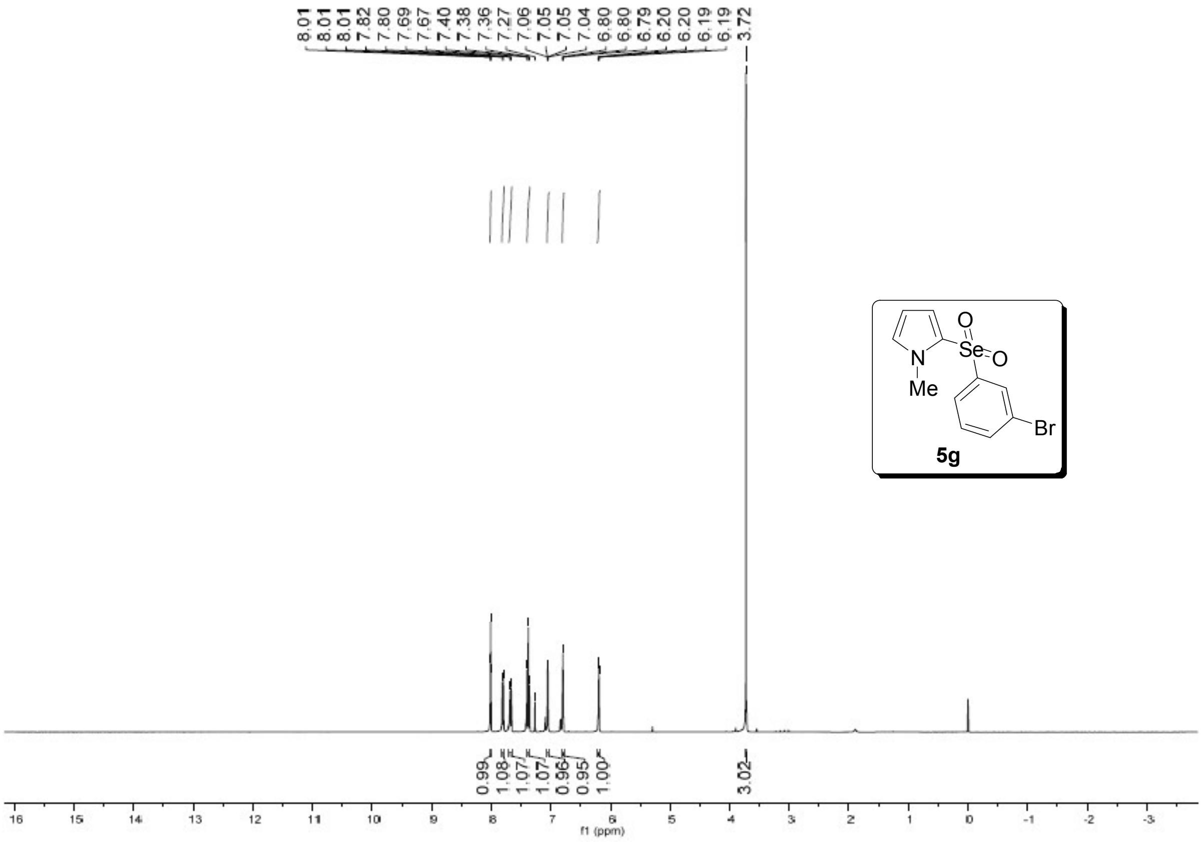

Figure 51. ^{1}H NMR **5g**

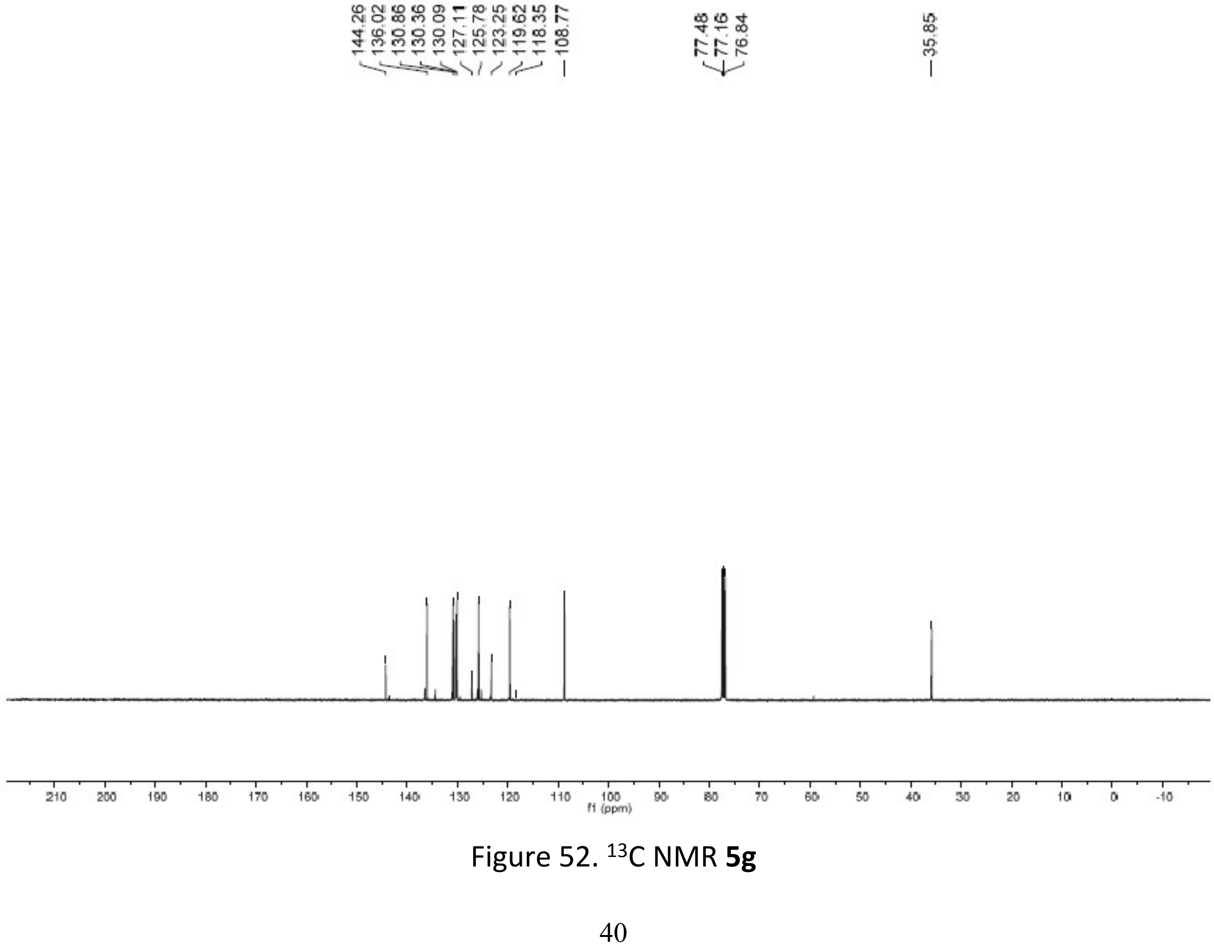

Figure 52. ^{13}C NMR **5g**

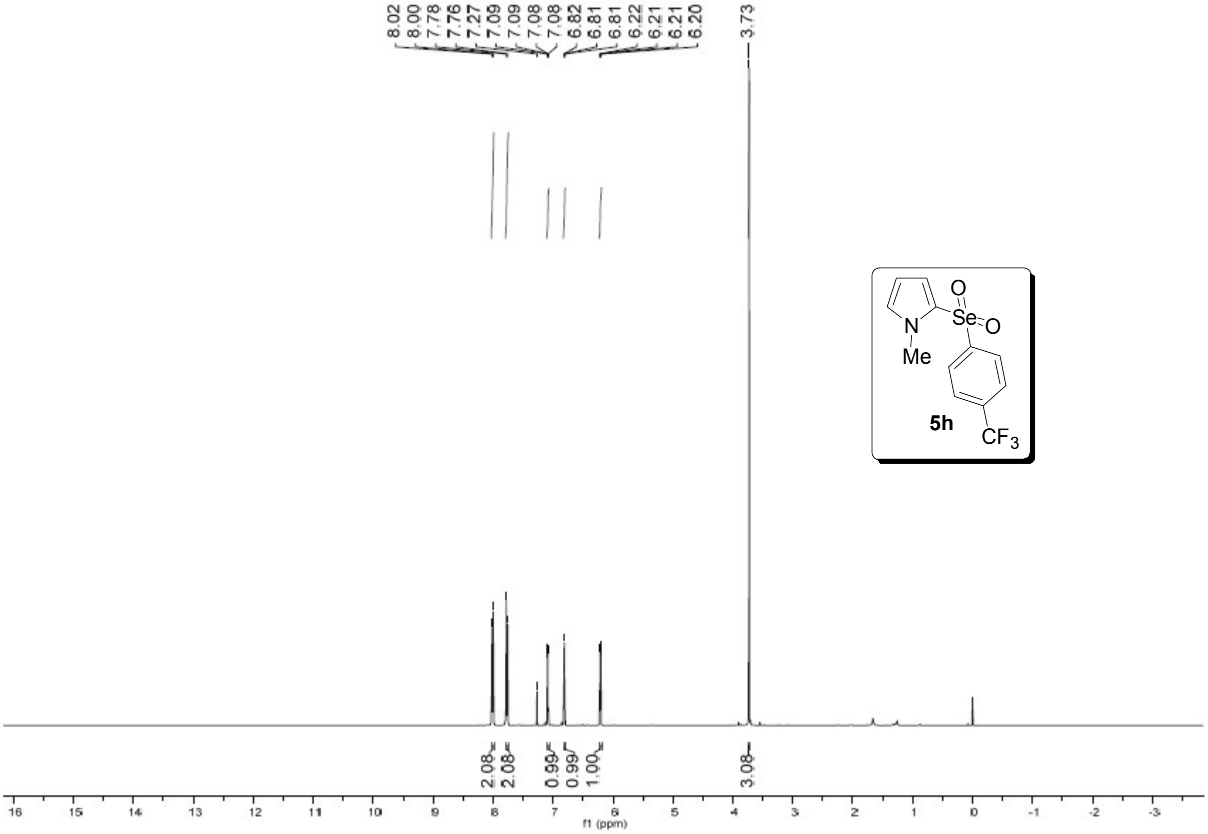

Figure 53. ^{1}H NMR **5h**

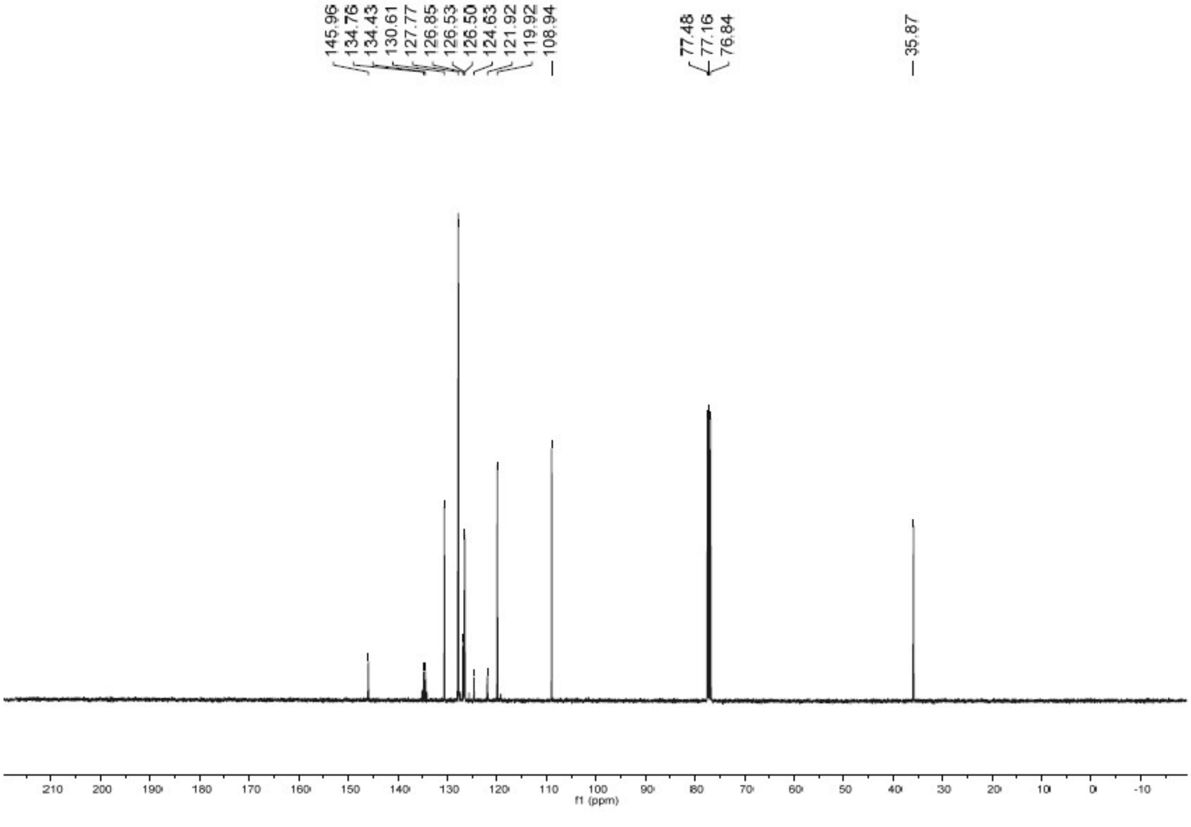

Figure 54. ^{13}C NMR **5h**

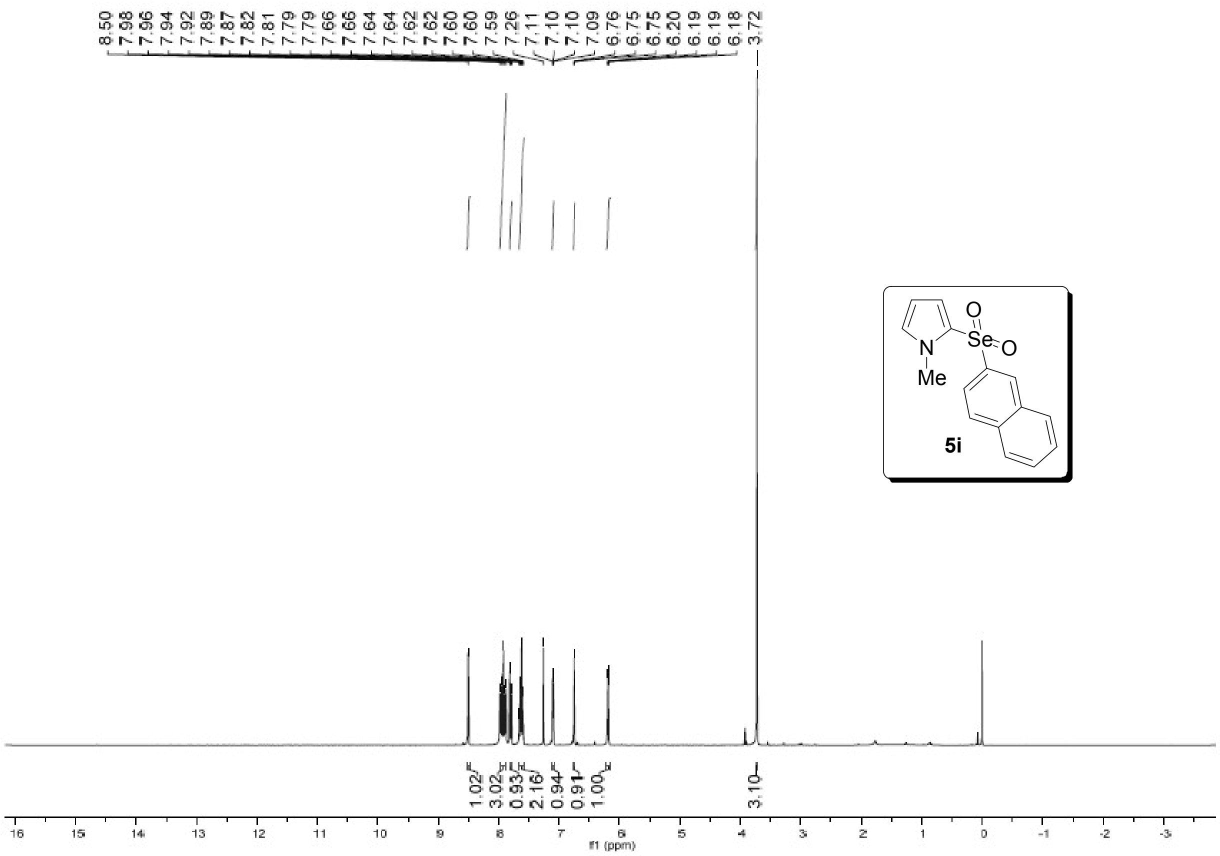

Figure 55. ^{1}H NMR **5i**

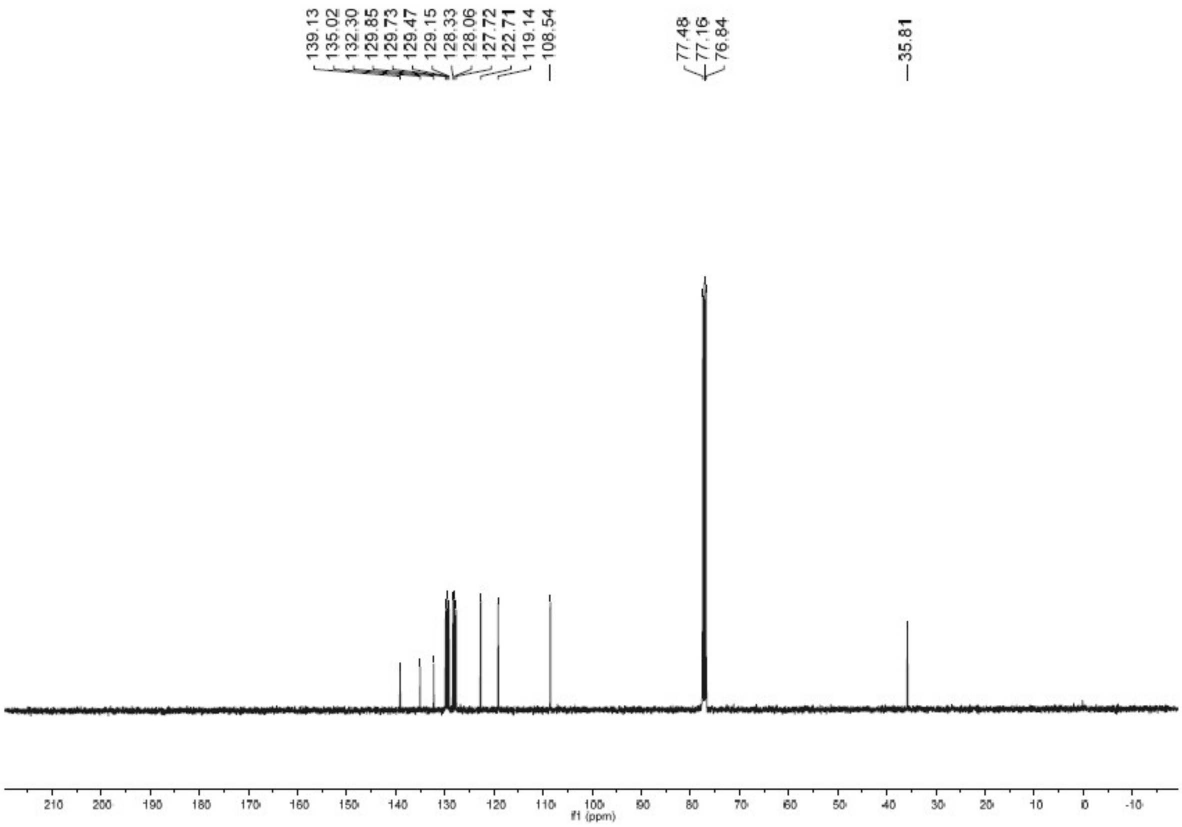

Figure 56. ^{13}C NMR **5i**

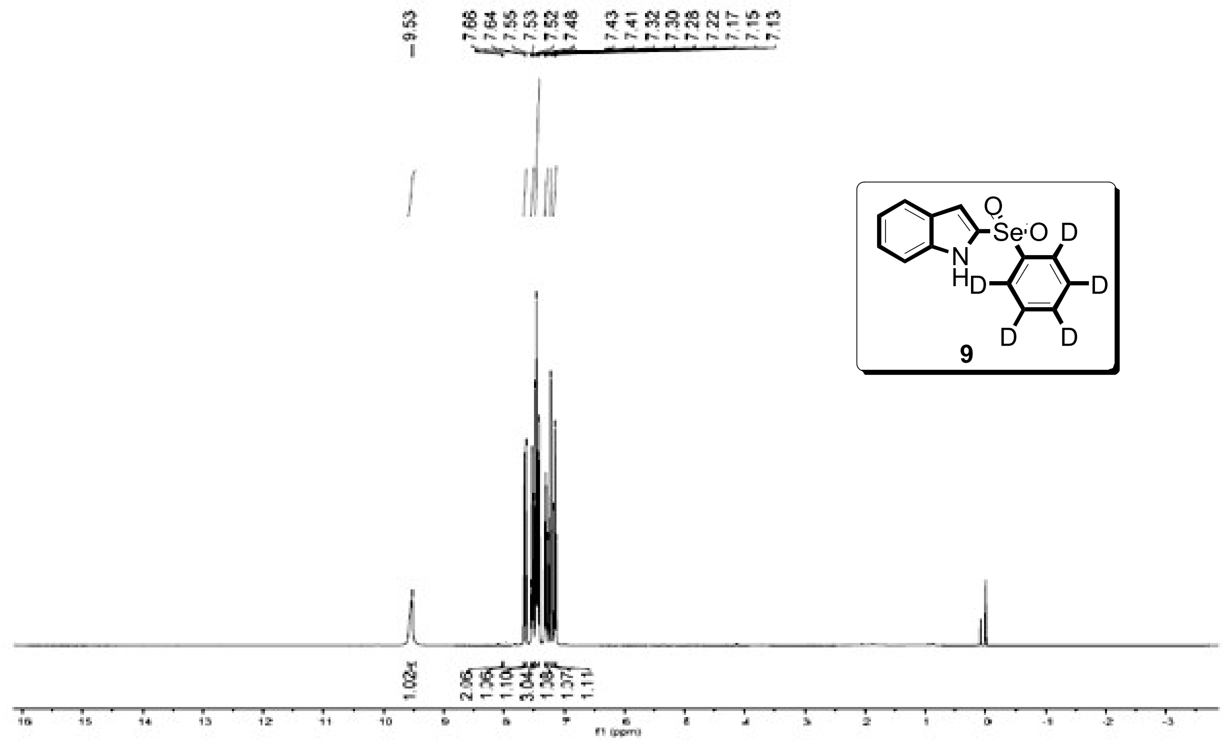

Figure 57. ^{1}H NMR **9**

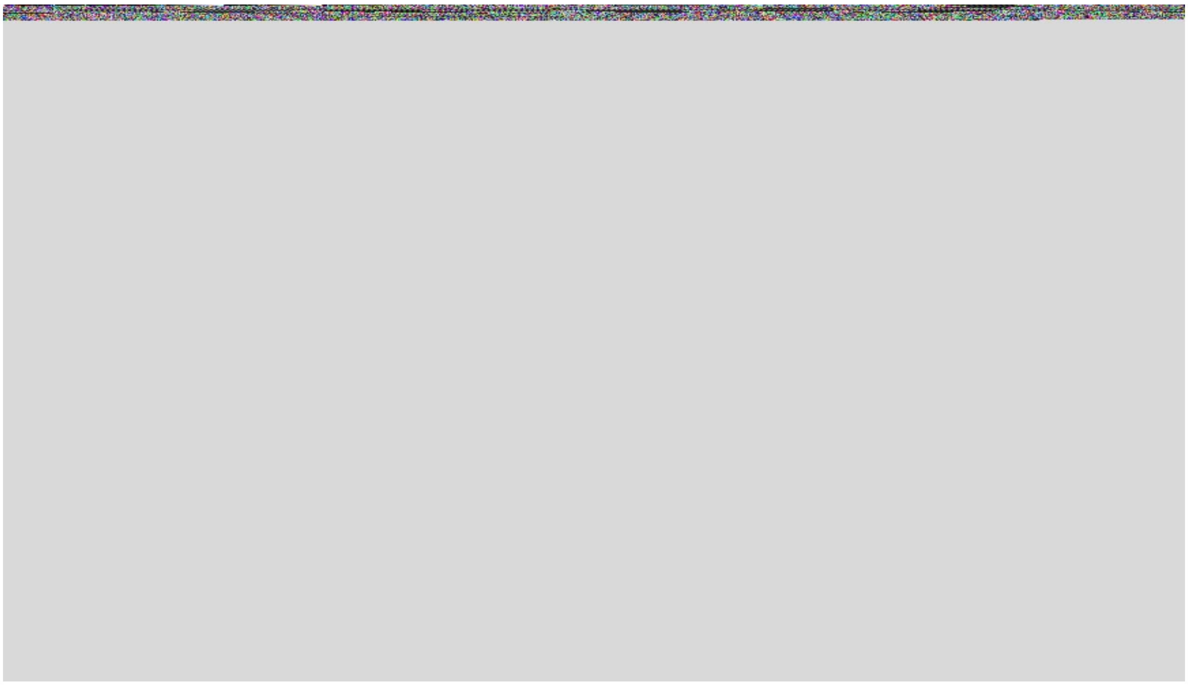

Figure 58. ^{13}C NMR **9**

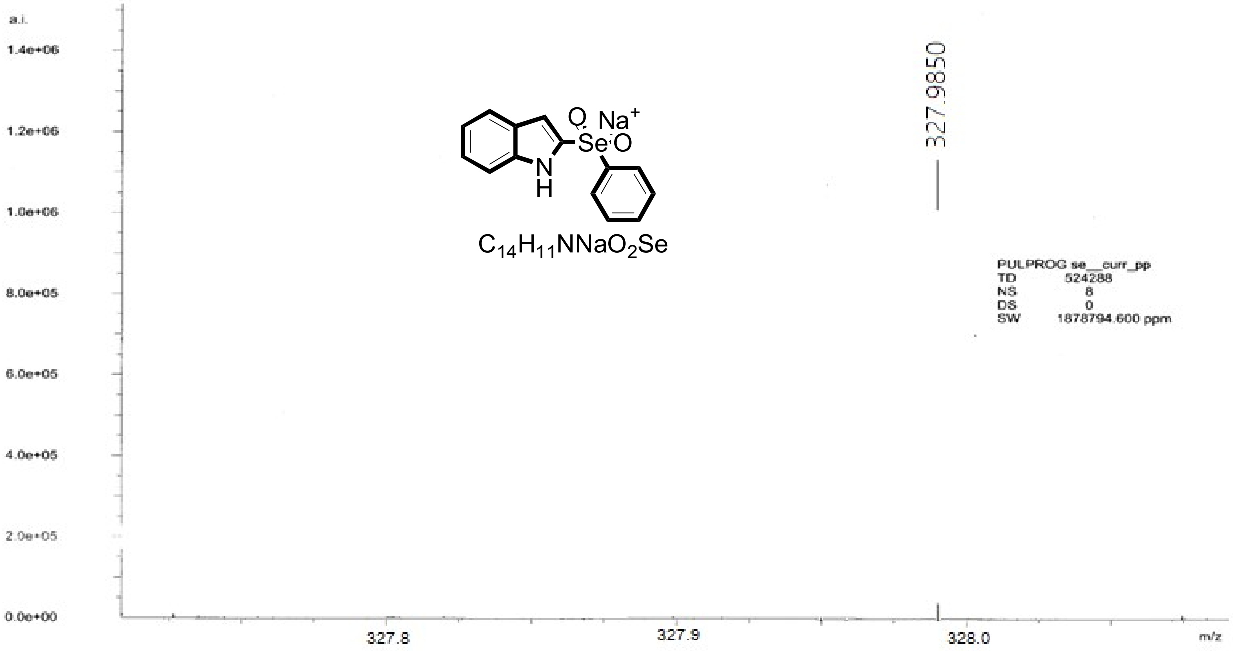

Figure 59. ESI HR-MS of **3a**

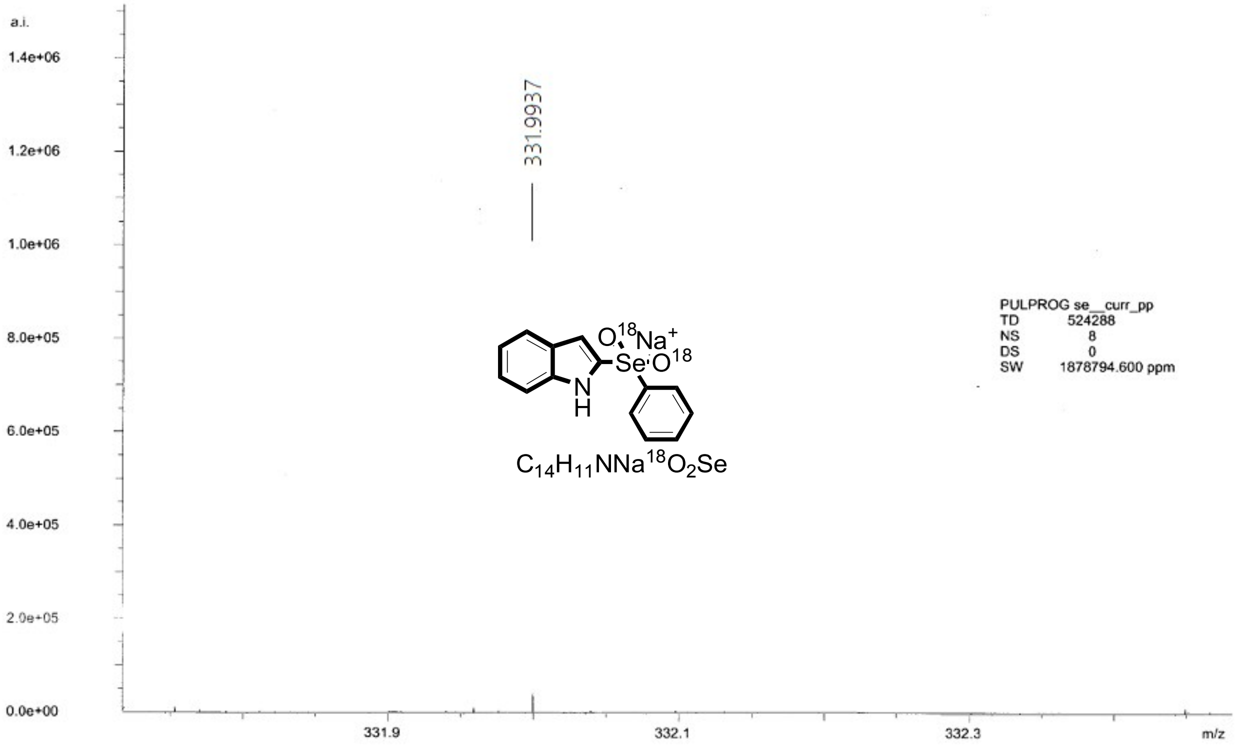

Figure 60. ESI HR-MS of $^{18}O_2$ deuterium labeling study **3a**
